# Comprehensive glycoside identification and anti-inflammatory activity screening in Fructus Gardeniae and Fructus Gardeniae Grandiflorae

**DOI:** 10.1016/j.fochx.2026.104006

**Published:** 2026-05-20

**Authors:** Xiaoyu Xie, Ruonan Zhang, Xueqin Yin, Zhangyang Shen, Chuntao Zeng, Xiuqiong Zhang, Weidong Dai

**Affiliations:** a Department of pharmacy, Jiangxi University of Chinese Medicine, Nanchang 330004, China; b Tea Research Institute, Chinese Academy of Agricultural Sciences, Hangzhou, Zhejiang 310008, China; c College of Chemistry and Chemical Engineering, Yunnan Normal University, Kunming 650500, China

**Keywords:** Glycoside identification, High-resolution mass spectrometry, Fructus Gardeniae, Fructus Gardeniae Grandiflorae, Anti-inflammatory

## Abstract

Glycosides are key bioactive constituents in foods and contribute to anti-inflammatory effects. Fructus Gardeniae (FG) and its variant Fructus Gardeniae Grandiflorae (FGG) exhibit distinct anti-inflammatory activities, yet their chemical basis remains unclear. However, the structural diversity of glycosides presents challenges for high-coverage identification. To overcome this limitation, a strategy combining large-scale glycosyl neutral-loss screening, *in silico* MS^2^ prediction, and MS^2^ database was developed for high-coverage identification. A total of 265 glycosides were identified or putatively identified, including 219 newly reported in FG and FGG. Statistical analysis revealed significant differences in 21 glycosides between FG and FGG. Molecular docking suggested that glycosides with significant differences may exhibit potential interactions with anti-inflammatory activity. Cellular assays further showed that two representative glycosides may exhibited anti-inflammatory effects under the tested conditions. Collectively, these findings provide a comprehensive characterization of the glycoside profiles of FG and FGG and highlight their potential for functional food applications.

## 1 Introduction

Glycosides are among the most widely distributed phytochemicals in food plants and are recognized as key contributors to the nutritional quality and health-promoting properties of plant-derived materials, owing to their diverse bioactivities such as anti-inflammatory, antioxidant, and metabolic regulatory effects (Joolaei Ahranjani et al., 2025; Kowsalya et al., 2025; Sirirungruang et al., 2023). Fructus Gardeniae (FG), a typical food-medicine homologous material, has long been utilized in both dietary and medicinal contexts (Qian et al., 2024). In commercial markets, two major forms are commonly distinguished, namely the smaller-sized FG and the larger-sized Fructus Gardeniae Grandiflorae (FGG) (Chen & Peng, 2018). Previous studies have reported differences in their anti-inflammatory activities (Cao et al., 2021; Liu et al., 2013). However, the chemical features associated with these functional differences remain insufficiently characterized. Considering that glycosides constitute the principal bioactive components in *Gardenia*-derived materials (Zhang et al., 2025), variations in their composition are likely to play a critical role in shaping their distinct biological functions. Therefore, comprehensive characterization of glycosides is essential for describing the chemical profiles of FG and FGG and for supporting subsequent investigations into their potential biological relevance.

Structurally, glycosides are composed of a glycosyl moiety linked to an aglycone through glycosidic bonds, with aglycones spanning diverse chemical classes such as flavonoids, terpenoids, iridoids, and organic acids (Zhang & Fernie, 2023). Meanwhile, glycosyl units consist of various monosaccharides, such as glucose, fucose, and galacturonic acid, as well as their modified forms, including acetylated, methylated, or multiply substituted sugars (Yu et al., 2022). This structural complexity poses significant analytical challenges for achieving high-coverage detection of glycosides. Liquid chromatography-mass spectrometry (LC-MS) has become the predominant analytical platform for glycoside profiling due to its high sensitivity and throughput (Ahmad et al., 2024; Dreisbach et al., 2022; Rodríguez et al., 2023). In particular, diagnostic glycosyl neutral-loss fragments generated during MS^2^ fragmentation are widely employed for targeted screening of glycosides in complex datasets (Goh et al., 2022; Zhang & Fernie, 2023). However, conventional LC-MS strategies typically rely on a limited set of predefined neutral-loss patterns, thereby restricting their ability to capture the full diversity of glycosides (Huang et al., 2025). Furthermore, the limited representation of glycosides in existing LC-MS databases further constrains comprehensive identification (Zhang et al., 2022). Collectively, these limitations hinder the development of high-coverage glycoside annotation workflows.

Analysis of reported glycoside structures indicates that most glycosides consist of monosaccharides, modified monosaccharides, or short oligosaccharides (typically two to four units) assembled through glycosidic linkages (Marinaccio et al., 2026; Rodríguez et al., 2023; Zhang et al., 2022). This structural regularity provides an opportunity for *in silico* prediction of glycosyl groups and their corresponding neutral-loss patterns. Accordingly, large-scale glycosyl neutral-loss libraries can be constructed through systematic *in silico* enumeration, enabling the expansion of detectable neutral-loss space beyond conventional empirical rules. Compared with MS^2^ databases, structural databases contain a substantially larger number of glycosides. For example, the COlleCtion of Open NatUral producTs (COCONUT) database includes more than 30,000 glycosides. Integrating these structural databases therefore enables broader identification of glycosides (Xie et al., 2022). However, structural databases only contain MS^1^ information of compounds, and such information alone often generates numerous candidate structures, thereby reducing identification confidence (Xie et al., 2024). To address this challenge, computational tools such as CFM-ID enables the *in silico* prediction of MS^2^ spectra from candidate structures and has been successfully applied in metabolite identification (Chen et al., 2022; Wang et al., 2021; Zhang et al., 2020). Nevertheless, their application in glycoside-focused workflows remains underexplored. Therefore, integrating large-scale glycosyl neutral-loss prediction with *in silico* MS^2^ generation offers a promising strategy to achieve high-coverage and more reliable glycoside identification in complex matrices.

**Fig. 1:**
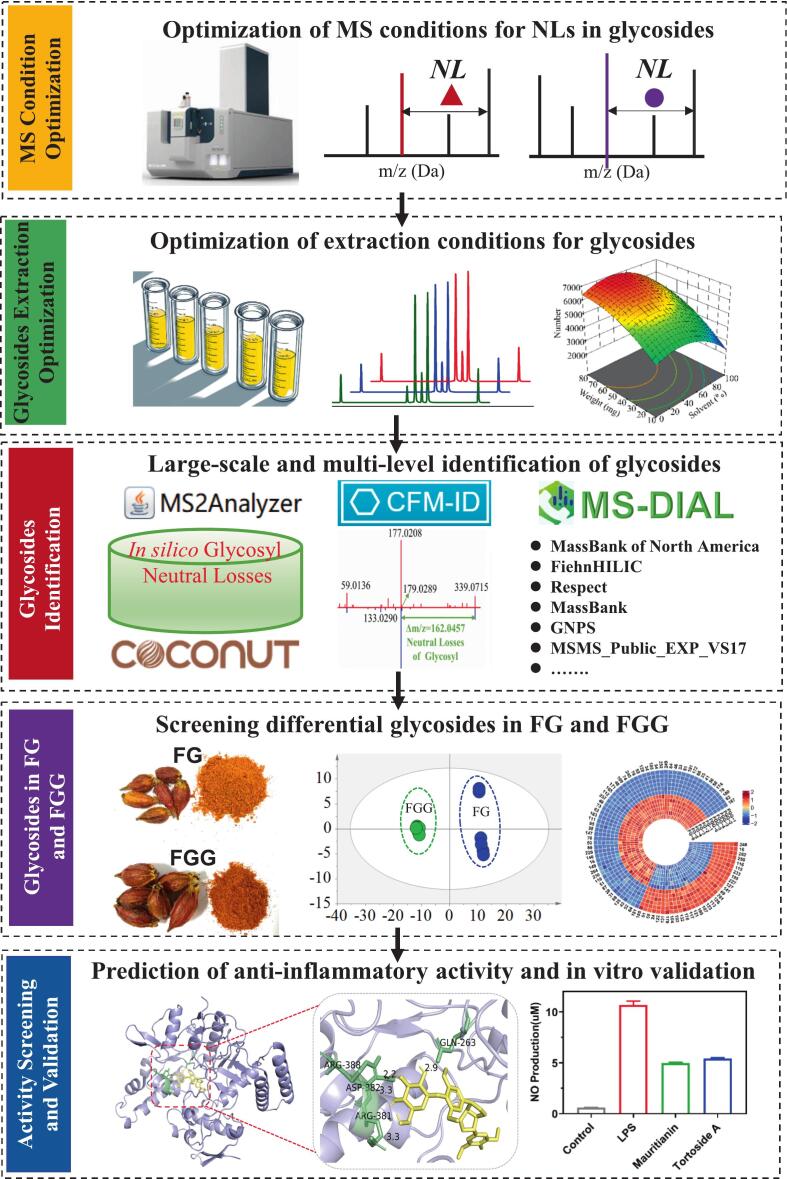
The workflow for analyzing glycosides in FG and FGG.

In this study, an integrated analytical workflow was developed to systematically characterize glycosides in FG and FGG. Specifically, MS parameters and ultrasonic extraction conditions were first optimized to enhance glycoside detection efficiency. Subsequently, a comprehensive identification strategy combining large-scale glycosyl neutral-loss screening, *in silico* MS^2^ prediction, and MS^2^ database matching was established. Based on the identified glycosides, foodomics-driven analyses were performed to uncover differential glycoside profiles between FG and FGG. Finally, molecular docking and cellular assays were conducted to evaluate the potential anti-inflammatory activity of glycosides with significant changes in FG and FGG (Fig. 1).

## 2 Materials and methods

### 2.1 Reagents and sample

LC-MS-grade methanol, acetonitrile (ACN), and formic acid were purchased from Merck (Darmstadt, Germany). Ultrapure water was prepared using a Milli-Q purification system. Ethanol and ammonium acetate were obtained from Inno-chem Science & Technology Co., Ltd. (Beijing, China). CCK-8 assay kits and lipopolysaccharide (LPS) were purchased from Beyotime (Shanghai, China). Griess reagent and ELISA kits were purchased from NeoBioscience Technology Co., Ltd. (Beijing, China). The glycoside standards were obtained from Macklin Biochemical Co., Ltd. (Shanghai, China) and dissolved in methanol-water mixtures at appropriate proportions.

FG and FGG were collected from Jiangxi Province, China, and authenticated by Professor Gong Qianfeng of Jiangxi University of Chinese Medicine. Six replicate samples were collected for both FG and FGG, each derived from six individual plants. After collection, the FG and FGG were dried under natural conditions. The dried materials were ground using a mechanical grinder and passed through a 40-mesh sieve to obtain uniform powders. The quality control (QC) sample was prepared by combining FG and FGG in equal weights. The blank sample consisted of 37% (*v*/v) ethanol. For extraction, 83 mg of the sample was accurately weighed into a 1.5 mL Eppendorf tube, followed by the addition of 1 mL of 37% ethanol. The mixture was subjected to ultrasonic extraction for 44 min and subsequently centrifuged at 13,000 rpm for 10 min. The resulting supernatant was used for ultrahigh-performance liquid chromatography coupled to high-resolution mass spectrometry (HPLC-HRMS) analysis.

### 2.2 Response surface methodology (RSM) for optimizing extraction conditions

RSM was employed to optimize ultrasonic extraction conditions for glycosides from FG and FGG. Based on previous studies (Dai et al., 2016; Marinaccio et al., 2025; Zhang et al., 2022) and practical operational considerations, ethanol concentration in the extraction solvent, ultrasonic extraction time, and sample weight were selected as variables. The ethanol concentration was varied from 0% to 100%, ultrasonic extraction time ranged from 20 to 60 min, and sample weight was adjusted between 10 and 80 mg. These parameter ranges were input into Design Expert 12 (Stat-Ease Inc., Minneapolis, USA). Five center points were included in the experimental design, which was generated using Design-Expert software. A response surface model was subsequently constructed, with ethanol concentration, ultrasonic extraction time, and sample weight as independent variables, and the number of glycoside-related neutral-loss peaks as the dependent variable.

### 2.3 HPLC-HRMS analysis

HPLC-HRMS analysis was performed using a Shimadzu 40A HPLC system coupled to a ZenoTOF 7600 high-resolution mass spectrometer. In positive ion mode, the mobile phase consisted of water containing 0.1% formic acid (A) and ACN (B). The gradient elution started at 5% B and was maintained for 1 min, increased to 80% B over 23 min, held at 80% B for 4 min, returned to 5% B within 0.1 min, and equilibrated for 2.9 min. The declustering potential (DP) and spray voltage were set to 60 V and 5500 V, respectively. In the negative ion mode, mobile phase A was a 5 mM aqueous solution, and mobile phase B was ACN. The gradient started at 5% B and was held for 1 min, increased to 80% B over 17 min, maintained at 80% B for 4 min, returned to 5% B within 0.1 min, and equilibrated for 2.9 min. The DP and spray voltage for this mode were set to 60 V and 4500 V, respectively. Chromatographic separation for both positive and negative ion analyses was performed using an ACQUITY BEH C18 column (2.1 mm × 100 mm, 1.7 μm; Waters, Milford, USA). The column temperature and sample manager temperature were set to 40 °C and 6 °C, respectively. The flow rate was set to 0.3 mL/min, and the injection volume was 2 μL. The pressures for both gas 1 and gas 2 were set at 50 psi, and the source temperature was maintained at 500 °C. The acquisition ranges for MS^1^ and MS^2^ were 150–1250 Da and 50–1250 Da, respectively. MS^2^ fragmentation was performed using collision-induced dissociation with a collision energy (CE) of 30 ± 15 eV. Information-dependent acquisition was employed as the secondary acquisition mode, monitoring 10 candidate ions per cycle. All analyses were performed in triplicate.

### 2.4 Identification of glycosides

Firstly, glycosyl modification structures frequently reported in the literature or documented in the COCONUT database were collected, and structural information on monosaccharides was compiled. Based on these data, common monosaccharide modification patterns were summarized. A glycosyl neutral-loss database was then constructed by integrating known glycosyl neutral losses, monosaccharide modification types, and reported polysaccharide glycosyl modifications.

**Fig. 2:**
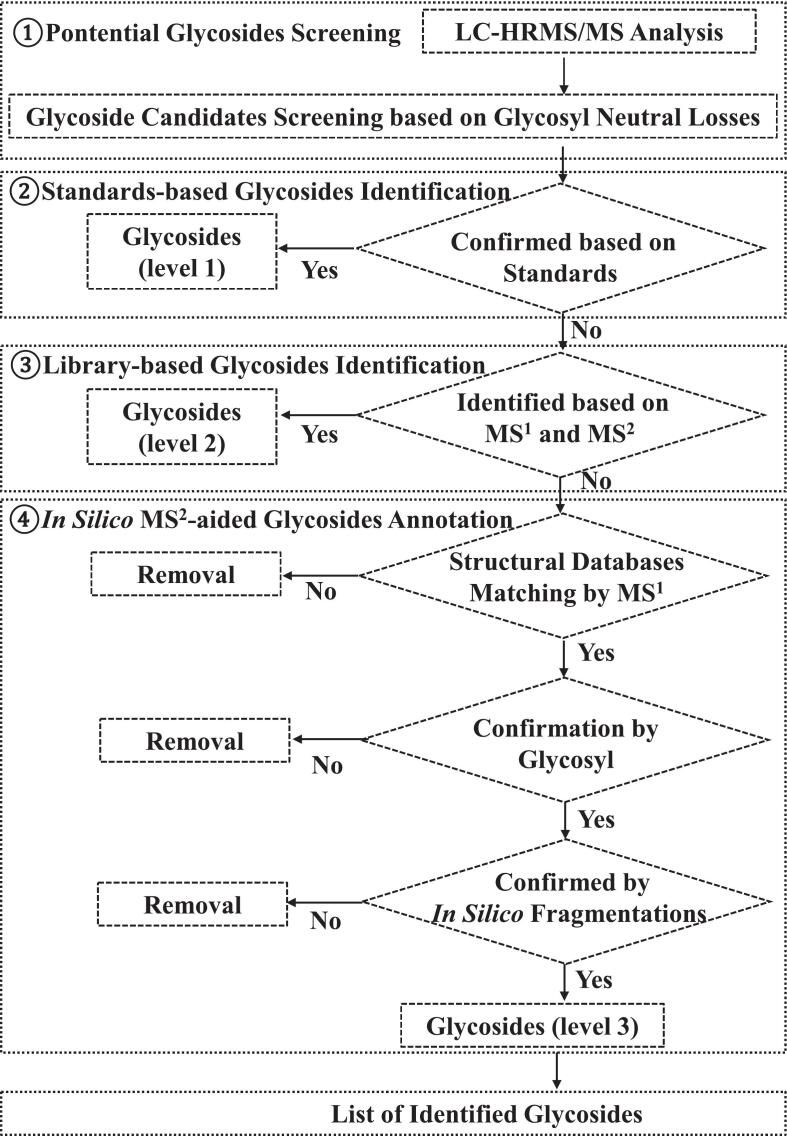
The strategy for identification of glycosides in FG and FGG.

Using this glycosyl neutral-loss database, a systematic workflow was established for glycoside identification in complex samples (Fig. 2). Briefly, glycosides were first screened using the MS2Analyzer program (Perez de Souza & Fernie, 2024) based on characteristic glycosyl neutral-loss features. Then the screened glycosides were identified using authentic standards (level 1). For glycosides that could not be verified with authentic standards, MS^2^ spectral matching was performed against multiple online databases, including the MS-DIAL authentic standards library, FiehnHILIC, GNPS, MassBank, Respect, and the Vaniya-Fiehn Natural Products Library utilizing both MS^1^ and MS^2^. Results based on putative identification with similarity scores ≥0.6 were accepted as level 2. For compounds not identification through online MS^2^ databases matching, candidate glycosides were retrieved from the structural database (COCONUT) based on MS^1^ information. Candidates inconsistent with the observed glycosyl neutral-loss features were excluded. The remaining candidates were subjected to *in silico* MS^2^ prediction using CFM-ID, and structures exhibiting similarity scores ≥0.5 between predicted and experimental MS^2^ spectra were retained (Charron-Lamoureux et al., 2025). When multiple candidates remained, the structure with the highest matching score was selected and assigned as a level 3 putative identification. It should be noted that level 2–3 identification are considered putative and may include structural ambiguities such as isomers.

### 2.5 Evaluation of anti-inflammatory activity

As previously described (Xie et al., 2025), the version of AutoDock Vina 1.2.6 was employed to perform molecular docking between inducible nitric oxide synthase (iNOS, PDB ID: 3E7G) and glycosides. iNOS was selected as the docking target due to its well-documented involvement in inflammatory responses (Speyer et al., 2003; Xie et al., 2025). In addition, previous studies have shown that glycoside compounds, including flavonoid and phenolic glycosides, can modulate inflammatory responses potentially through interactions with iNOS or related pathways (Fang et al., 2005; Liu et al., 2021). However, the anti-inflammatory effects are likely mediated through multiple interconnected inflammatory pathways rather than exclusively through direct modulation of iNOS. Accordingly, the molecular docking analysis was employed as a preliminary and exploratory computational approach to evaluate potential interaction tendencies, rather than to provide definitive mechanistic evidence of direct iNOS targeting. The 3D structure of iNOS was obtained from the Protein Data Bank (PDB, https://www.rcsb.org/), while the structures of glycosides were downloaded from PubChem database (https://pubchem.ncbi.nlm.nih.gov/). The receptor was prepared by removing water molecules, while the heme cofactor located in the catalytic site was retained during receptor preparation to preserve the structural integrity and catalytic environment of the active pocket. Polar hydrogens were subsequently added to the receptor, whereas ligands were subjected to geometry optimization and protonation prior to docking. The docking grid box was defined based on the position of the co-crystallized ligand and centered on the catalytic active site of iNOS, with dimensions sufficiently large to fully encompass the substrate-binding pocket and surrounding key residues. Docking calculations were performed using AutoDock Vina, and the resulting binding poses were analyzed using PyMOL (version 3.1.8). The binding conformation with the lowest predicted binding energy was selected for analysis. To validate the docking protocol, the co-crystallized ligand was first extracted from the protein crystal structure and re-docked into the original binding pocket using the same docking parameters as those applied for the test compounds (Trott & Olson, 2010; Meng et al., 2011). The reliability of the docking procedure was evaluated by calculating the root mean square deviation (RMSD) between the re-docked pose and the crystallographic conformation. The obtained RMSD value was 1.13 Å, which was below the generally accepted threshold of 2.0 Å, indicating satisfactory reproduction of the native binding mode and supporting the reliability of the docking protocol (Warren et al., 2006).

RAW264.7 cells in logarithmic growth phase were seeded into a 96-well plate at a density of 1 × 10^5^ cells per well. Cells were subsequently treated with mauritianin or tortoside A at concentrations of 0, 10, 20, 40, 80, 120, 160, and 200 μM for 24 h. Cell viability was then assessed using the CCK-8 assay, as previously described (Yin et al., 2022). Additionally, an LPS-stimulated RAW264.7 cells model was established to evaluate the anti-inflammatory activity of mauritianin and tortoside A. Specifically, RAW264.7 cells (1 × 10^5^ cells per well) were seeded into a 96-well plate and incubated for 24 h. After removal of culture medium, cells were treated with 1 μg/mL of LPS in combination with mauritianin and tortoside A at a concentration of 20 μM for 12 h. The supernatant was then collected, and nitric oxide (NO) levels were quantified using the Griess reagent assay. In addition, the concentrations of tumor necrosis factor-alpha (TNF-α) and interleukin-6 (IL-6) were determined using enzyme-linked immunosorbent assay (ELISA).

### 2.6 Statistical analysis and data visualization

Parameters used for peak extraction and identification in MS-DIAL were set according to previously reported methods (Yan et al., 2025). Partial least squares-discriminant analysis (PLS-DA) was performed using SIMCA 13 software (Umetrics, Umeå, Sweden). Student's *t*-test was performed using IBM SPSS Statistics 25.0 (IBM Corp., Armonk, NY, USA). To improve statistical robustness, multiple testing correction was performed using the false discovery rate (FDR) method. Volcano plots were generated using GraphPad Prism 8.0 software (GraphPad Software, La Jolla, CA, USA). Heat maps were generated using the online platform OmicStudio (https://www.omicstudio.cn).

## 3 Results and discussion

### 3.1 Optimization of the MS conditions

The neutral loss peaks of the aglycone play a crucial role in the identification of glycosides. Therefore, it is essential to select MS parameters that preserve the intensity of these neutral-loss peaks. Based on this principle, the DP was optimized using the relative intensities of aglycone neutral-loss peaks from authentic standards. The DP was evaluated at 20 V, 40 V, 60 V, 80 V, 100 V, 120 V, and 140 V. As illustrated in Fig. S1A and C, the relative intensities of aglycone neutral-loss peaks varied among different glycosides in both positive and negative ion modes. Notably, at a DP of 60 V, aglycone neutral-loss peaks for four glycosides exhibited relatively high intensities. During the optimization of the CE at 20 ± 15 eV, 30 ± 15 eV, 40 ± 15 eV, 50 ± 15 eV, and 60 ± 15 eV, a CE of 30 ± 15 eV produced relatively high aglycone neutral-loss intensities for the four glycosides (Fig. S1B and D). Given that this study employs an untargeted approach for glycoside detection, the MS conditions needed to provide robust and consistent responses across diverse glycosides. Accordingly, the optimal DP and CE were determined to be 60 V and 30 ± 15 eV, respectively.

### 3.2 Optimization of the extraction conditions of glycosides

To enhance the glycoside extraction from FG or FGG, RSM was employed to optimize extraction parameters, and statistical analysis was systematically applied to evaluate the significance and reliability of the optimization process. As summarized in Table S1, the optimized conditions increased the number of detected glycoside-related features from 2536 to 6943, demonstrating the substantial impact of extraction conditions on glycoside coverage. Table S2 presents the evaluation results of the RSM model based on analysis of variance (ANOVA). The results indicated that the model was statistically significant (*p* < 0.0001) and exhibited a non-significant lack of fit (*p* = 0.1482), indicating good model adequacy. The coefficient of determination (R^2^) of the model was 0.92, with an adjusted R^2^ of 0.98, demonstrating a high degree of correlation between predicted and experimental values. The difference between R^2^ and adjusted R^2^ was less than 0.2, suggesting strong agreement between predicted and experimental values (Xie et al., 2022). Additionally, the adequate precision value was 38, exceeding the acceptable threshold of 4 and indicating a robust signal-to-noise ratio (Xie et al., 2023). The coefficient of variation (CV) was 2.86%, well below the acceptable limit of 10%, confirming good model reproducibility (Hu et al., 2025). These statistical indicators collectively demonstrate the robustness, precision, and predictive capability of the RSM model in extraction optimization. Overall, the RSM model established in this study was reliable for predicting optimal extraction conditions.

**Fig. 3:**
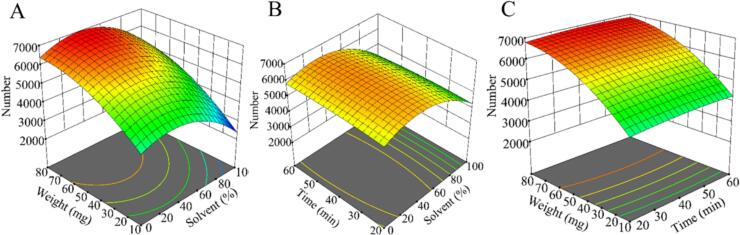
Optimization of the extraction conditions (A) the interaction effects of weight and solvent on the number of potential glycosides (B) the interaction effects of time and solvent on the number of potential glycosides (C) the interaction effects of weight and time on the number of potential glycosides.

To more clearly illustrate the influence of extraction conditions on extraction efficiency, 3D response surface plots were generated. As shown in Fig. 3, the interaction between sample weight and the extraction solvent composition exerted the strongest effect on extraction efficiency (Fig. 3A). This was followed by the interaction between ultrasonic extraction time and solvent composition (Fig. 3B), whereas the interaction between ultrasonic time and sample weight showed a relatively minor effect (Fig. 3C). Overall analysis of Fig. 3 indicates that sample weight and the solvent composition significantly influence extraction efficiency, whereas ultrasonic extraction time has a comparatively minor effect. Finally, the model was employed to predict the optimal extraction conditions. The results indicated that the highest extraction efficiency was achieved using 37% ethanol as the extraction solvent, an ultrasonic extraction time of 44 min, and a sample weight of 82 mg, with a predicted number of 6987 glycoside-related features.

### 3.3 Identification of glycosides in QC sample

#### 3.3.1 Construction of glycosyl neutral losses

A total of 63 monosaccharides were collected (Table S3) based on their occurrence frequency in natural glycosides and structural diversity. Structural analysis revealed that acetylation (one to three substitutions) and methylation (one to four substitutions) were the most frequent monosaccharide modification types. Among these, six monosaccharides namely oxane-2,3,4,5-tetrol, 5-(hydroxymethyl)oxolane-2,3,4-triol, 6-methyloxane-2,3,4,5-tetrol, 4-(hydroxymethyl)oxolane-2,3,4-triol, 6-(hydroxymethyl)oxane-2,3,4,5-tetrol, and 3,4,5,6-tetrahydroxyoxane-2-carboxylic acid were frequently observed in native, modified, and oligosaccharide (2-4 units) forms. Neutral-loss candidates were therefore systematically generated by enumerating combinations of these core monosaccharides, their typical modification patterns, and oligomerization states. This resulted in 468 predicted neutral losses from the six core structures. In addition, for the 63 known monosaccharides two cleavage modes were further considered depending on whether the glycosidic oxygen was retained or lost during fragmentation, and all corresponding elemental compositions were computed and deduplicated. By integrating these rules, a comprehensive database comprising 594 glycosyl neutral losses was constructed, and detailed information is provided in Table S4.

#### 3.3.2 Identification of glycosides in FG and FGG

Under optimized extraction and MS acquisition conditions, FG and FGG samples were analyzed, and the resulting data were collected. The acquired data were used for glycoside identification following the workflow illustrated in Fig. 2. A total of 265 glycosides were identified or putatively identified (Table S5), representing high-coverage glycoside profiling. As summarized in Table S5, these 265 glycosides comprised 74 terpene glycosides, 38 flavonoid glycosides, 30 cinnamic acid glycosides, 27 benzene glycosides, 12 steroid glycosides, 11 glycosylglycerols, 10 glycosphingolipids, 10 lignan glycosides, 9 coumarin glycosides, 6 glycerophospholipids, and 38 glycosides belonging to other structural classes. Furthermore, 12 glycosides were confirmed using authentic standards (Fig. S2). Notably, among the 12 glycosides confirmed using authentic standards, 10 have documented anti-inflammatory effects, whereas the anti-inflammatory activities of mauritianin and tortoside A have not yet been reported. Among the remaining 253 compounds, 152 were putatively identified based on combined MS^1^ and MS^2^ database searches, whereas 101 were putatively identified using MS^1^ combined with *in silico* MS^2^ prediction.

**Fig. 4:**
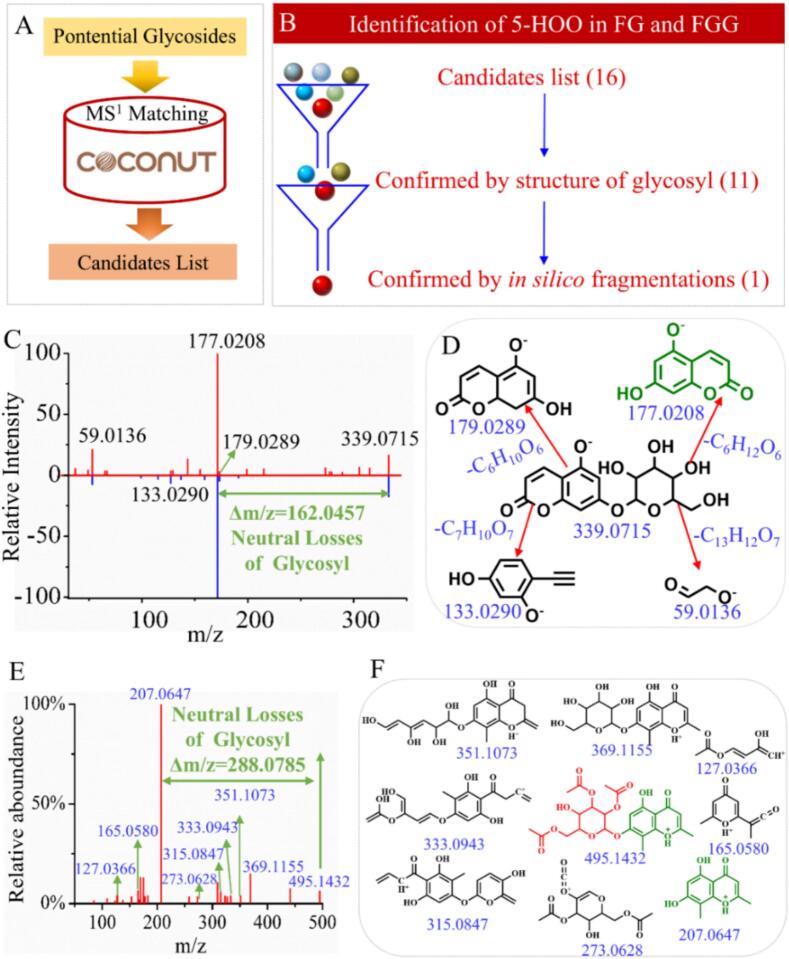
(A) The process of obtaining candidates. (B) The process of confirming the peak at t_R_ = 3.3 min and m/z = 339.0715 as 5-hydroxy-7-{[3,4,5-trihydroxy-6-(hydroxymethyl)oxan-2-yl]oxy}-2H-chromen-2-one (5-HOO). (C) The experimental MS^2^ (blue) and *in silico* MS^2^ (red) of 5-HOO. (D) Structure elucidation of MS^2^ of 5-HOO. (E) The experimental MS^2^ of [4,5-diacetyloxy-3-hydroxy-6-(5-hydroxy-2,8-dimethyl-4-oxochromen-7-yl) oxyoxan-2-yl] methyl acetate (4,5-DMA). (F) Structure elucidation of MS^2^ of 4,5-DMA. (For interpretation of the references to colour in this figure legend, the reader is referred to the web version of this article.)

To illustrate the glycoside annotation process based on MS^1^ and *in silico* MS^2^, the putative identification of 5-hydroxy-7-{[3,4,5-trihydroxy-6-(hydroxymethyl)oxan-2-yl]oxy}-2H-chromen-2-one (5-HOO) is presented as a representative example. Initially, using the established glycosyl neutral-loss database, the MS^2^ spectrum corresponding to a peak at t_R_ = 3.3 min and *m*/*z* = 339.0715 revealed a neutral loss fragment with a mass difference of 162.0457 Da (m/z = 177.0208). This observation suggested the presence of a glucosyl moiety within the molecular structure. As neither authentic standards nor online MS^2^ databases searches provided a definitive identification, a query of the COCONUT database using m/z 339.0715 was performed to retrieve potential glycoside candidates (Fig. 4A). Initially, 16 glycosides matching the MS^1^ information were retrieved from the COCONUT database. Subsequently, candidates lacking a glucose moiety were excluded, reducing the candidate list to 11 glycosides. These candidates were then subjected to MS^2^ spectrum prediction using the CFM-ID, and the similarity between the predicted and experimental MS^2^ was evaluated. The compound exhibiting the highest similarity score was selected as the most probable structure (Fig. 4B). In this study, the predicted MS^2^ spectrum of 5-HOO showed the highest similarity to the experimental MS^2^ spectrum, with a score of 0.71 (Fig. 4C). Accordingly, the peak at t_R_ = 3.3 min and *m*/*z* = 339.0715 was assigned to 5-HOO. To further support this assignment, the experimental MS^2^ spectra were structurally annotated, as shown in Fig. 4D. All major fragment ions could be rationalized as fragmentation products of 5-HOO. Overall, this approach integrates glycosyl neutral-loss information with *in silico* MS^2^ prediction to refine candidate structures, thereby reducing the complexity associated with glycoside identification.

A review of the literature on FG and FGG components revealed that, among the 265 identified or putatively identified glycosides, 219 had not been previously reported. This finding indicates that the proposed method has strong potential for identifying previously unreported glycosides. For example, the glycosyl neutral-loss database developed in this study facilitates the identification of previously unreported glycosyl modifications in FG and FGG. Notably, a distinct neutral-loss peak corresponding to tri-acetylated glucose was observed in the MS^2^ spectrum of peak at t_R_ = 11.7 min and m/z = 495.1460, which has not been reported in FG and FGG (Fig. 4E). This observation suggests that the compound eluting at this t_R_ is a glycoside modified with tri-acetylated glucose. Following an identification procedure analogous to that applied to 5-HOO, this compound was characterized as [4,5-diacetyloxy-3-hydroxy-6-(5-hydroxy-2,8-dimethyl-4-oxochromen-7-yl) oxyoxan-2-yl] methyl acetate (4,5-DMA). To validate this proposed identification, the experimental MS^2^ spectrum was structurally annotated. As shown in Fig. 4F, the majority of the fragment ions corresponded to cleavage products of 4,5-DMA.

### 3.4 Comparison of glycosides between FG and FGG

**Fig. 5:**
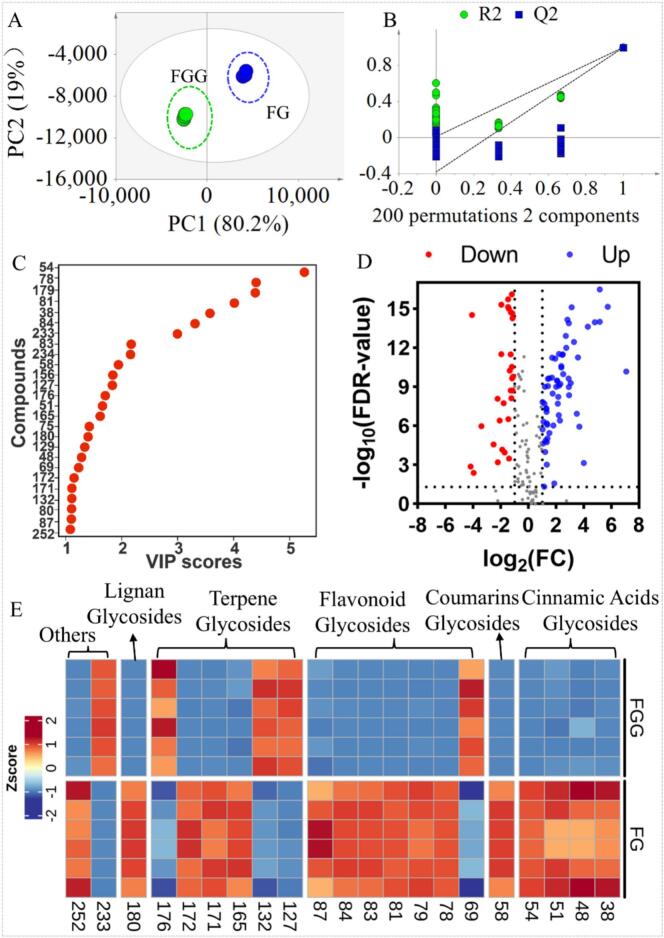
Statistical analysis results of FG and FGG. (A) PLS-DA score plots. (B) PLS-DA model permutation test. (C) The compounds with VIP values greater than 1.0. (D) volcano plot. (E) Heatmap of potential chemical markers. FG represents Fructus Gardeniae and FGG represents Fructus Gardeniae Grandiflorae. Numbers shown in the heatmap correspond to the compound numbers listed in Table S5.

To investigate the differences in glycoside profiles between FG and FGG, statistical analyses were performed. Fig. 5A illustrates the PLS-DA model constructed based on the identified glycosides following data normalization and Pareto scaling. The model showed clear separation between FG and FGG samples, underscoring substantial differences in their glycoside compositions. The R^2^X of the model was 0.992, indicating effective capture of the key information of the independent variables. In addition, the R^2^Y was 0.998, demonstrating the model's strong capacity to explain the differences between the groups. The Q^2^ was 0.996, suggesting robust predictive performance. Fig. 5B presents the results of 200 permutation tests performed on the PLS-DA model. The R^2^ and Q^2^ obtained from permutation testing were 0.011 and − 0.38, respectively, both substantially lower than those of the original model. These results indicate that the PLS-DA model did not exhibit overfitting. Fig. 5C presents the compounds with variable importance in projection (VIP) values greater than 1.0. A total of 26 compounds exhibited VIP values greater than 1.0, ranging from 1.1 to 5.3, indicating their substantial contribution to group discrimination.

Fig. 5D illustrates the distribution of fold change (FC) and FDR values for glycosides between FG and FGG. The results showed that 62 glycosides were more abundant in FG, whereas 38 exhibited lower abundances compared with FGG. Fig. 5E presents a heat map of glycosides meeting the criteria of FC > 2 or < 0.5, FDR < 0.05, and VIP > 1. Among the 21 glycosides showing significant differences, 16 were more abundant in FG, while 5 were more abundant in FGG (Fig. 5E and Table S6). These 21 differential glycosides included 4 cinnamic acid glycosides, 1 coumarin glycoside, 7 flavonoid glycosides, 6 terpene glycosides (including iridoid glycosides, *e.g.*, No. 165, 171, 172, and 176), 1 lignan glycoside, and 2 belonging to other structural classes. Notably, all four cinnamic acid glycosides were more abundant in FG. Among the flavonoid glycosides, only one was more abundant in FGG, whereas the remaining six showed higher levels in FG. Among the terpene glycosides, three were more abundant in FG, all of which were iridoid glycosides. Cinnamic acid glycosides are known to exhibit diverse biological activities, including antioxidant and anti-inflammatory effects (Ruwizhi & Aderibigbe, 2020). Iridoid glycosides constitute major pharmacologically active components of FG, contributing to anti-inflammatory, hepatoprotective, antioxidant, and immunomodulatory effects (Hassan et al., 2025). Flavonoid glycosides also exhibit biological activities, including antioxidant, anti-inflammatory, and antitumor effects (Al-Khayri et al., 2022). Most of these glycosides were present at higher levels in FG, suggesting differences in biological properties. Anti-inflammatory activity represents a key biological function of both FG and FGG (Liu et al., 2013). However, previous studies have reported differences in anti-inflammatory efficacy between FG and FGG (Fu et al., 2001), suggesting that distinct glycoside compositions may contribute to their differential anti-inflammatory effects.

### 3.5 Evaluation of potential anti-inflammatory activity of glycosides with significant changes

To investigate the anti-inflammatory activities of glycosides showing significant changes in FG and FGG, a comprehensive literature survey was first conducted to assess whether these compounds had previously been reported to exhibit anti-inflammatory effects. As summarized in Table S6, among the 21 glycosides showing significant changes, 6 have been previously reported to exhibit anti-inflammatory activity. For the remaining glycosides without reported anti-inflammatory activity, molecular docking was employed to provide preliminary insights into their potential interactions related to anti-inflammatory processes. The docking protocol was validated by re-docking the co-crystallized ligand into the active pocket of 3E7G. The best-scored re-docked pose closely overlapped with the crystallographic conformation, with an RMSD value of 1.13 Å (Fig. S3A), indicating that the docking protocol is reliable for reproducing the native binding mode. In addition, the randomly selected glycosides glycoside (tortoside A) was found to occupy the same binding pocket as the co-crystallized ligand (Fig. S3B), suggesting a comparable binding region and supporting the structural plausibility of the predicted binding modes. The lowest predicted binding energies between the remaining 15 glycosides and the iNOS protein were calculated (Table S7). As shown in Table S7, the binding energies ranged from -6.9 to -11.3 kcal/mol, with values comparable to that of the co-crystallized ligand (-8.3 kcal/mol), suggesting a generally favorable binding tendency. These results indicate a generally favorable binding tendency. All in all, combined with literature evidence, these findings suggest that the glycosides showing significant changes may be associated with potential interactions relevant to anti-inflammatory processes.

It should be noted that the docking results require cautious interpretation, particularly for large glycosylated compounds. Due to their relatively large size and structural complexity, such molecules may have limited accessibility to the deeply buried catalytic pocket of iNOS because of steric constraints. In this context, the observed docking interactions may reflect general surface interaction tendencies rather than definitive catalytic-site binding. However, this does not preclude their potential biological activity. Previous studies have reported that glycosylated natural products can exert anti-inflammatory effects by inhibiting the expression of iNOS in LPS-stimulated models (Liu et al., 2021). Accordingly, molecular docking in this study was employed primarily as a preliminary and exploratory screening tool to assess potential interaction tendencies, rather than as definitive evidence of precise binding modes or direct target engagement.

**Fig. 6:**
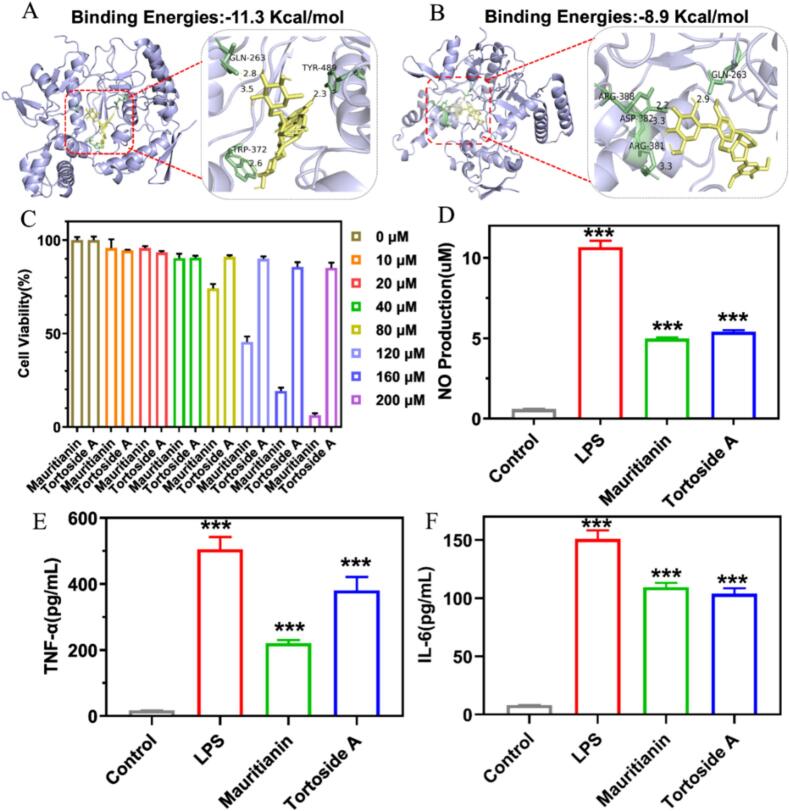
Anti-inflammatory activity of mauritianin and tortoside A. (A) molecular docking of mauritianin. (B) molecular docking of tortoside A. (C) cell viability of RAW264.7 cells. (D) inhibition of NO production. (E) inhibition of TNF-α. (F) inhibition of IL-6. *** *P* < 0.001.

Here, mauritianin and tortoside A were selected as representative compounds for preliminary experimental validation, as they were structurally confirmed using authentic standards, exhibited significant abundance differences between FG and FGG, and lacked prior reports of anti-inflammatory activity. As illustrated in Fig. 6A, mauritianin formed four hydrogen bonds with GLN-263, TYR-489, and TRP-372, with a binding energy of −11.3 kcal/mol, indicating favorable interactions with iNOS and suggesting possible biological relevance. Similarly, tortoside A exhibited a binding energy of −8.9 kcal/mol and formed four hydrogen bonds with ARG-388, GLN-263, ASP-382, and ARG-381 (Fig. 6B), also showing favorable interaction with iNOS and implying potential biological relevance. To validate the molecular docking results, RAW264.7 cells were used to evaluate the anti-inflammatory activities of mauritianin and tortoside A. As shown in Fig. 6C, RAW264.7 cells treated with 10, 20, and 40 μM of mauritianin exhibited cell viabilities comparable to those of the control group. However, at higher concentrations, RAW264.7 cell viability decreased significantly. In contrast, RAW264.7 cells treated with varying concentrations of tortoside A exhibited no significant differences in cell activity. Accordingly, a concentration of 20 μM was selected for both compounds in subsequent experiments. As shown in Fig. 6D, both mauritianin and tortoside A significantly inhibited NO production. In addition, both compounds significantly reduced the levels of TNF-α and IL-6 (Fig. 6E-F). Collectively, these results suggest that mauritianin and tortoside A may exhibit anti-inflammatory effects under the tested conditions, while the underlying mechanisms remain to be further elucidated. However, although NO inhibition was observed, direct evidence linking these effects specifically to iNOS modulation (*e.g.*, protein expression or enzyme activity) was not investigated in this study and requires further validation.

It should be noted that most compounds in this study were assigned at the level of putative identification (level 2–3) due to due to the limited availability of authentic standards. As such, structural ambiguities, including the presence of isomers and unresolved stereochemistry, cannot be fully excluded. However, these putative identifications were supported by integrated MS^1^ and MS^2^ evidence, providing a reasonable level of structural confidence. According to the Metabolomics Standards Initiative, putative identified metabolites can be used for downstream analysis when appropriately described and interpreted with caution (Summer et al., 2007; Schymanski et al., 2014). In this context, the subsequent statistical analysis and molecular docking in this study were conducted primarily as exploratory and hypothesis-generating approaches rather than definitive mechanistic validation. Molecular docking, as a structure-based computational method, operates on plausible molecular models and does not strictly require fully confirmed structures (Meng et al., 2011; Morris et al., 2009). This strategy has been widely adopted in natural product research to prioritize bioactive candidates and to infer potential interactions between putative identified compounds and biological targets (Pinzi & Rastelli, 2019; Wolfender et al., 2019). Overall, while structural uncertainties remain, the current workflow provides a practical and widely accepted framework for large-scale glycoside profiling and rational prioritization of candidate compounds for subsequent targeted isolation and structural validation.

## 4 Conclusion

In this study, a comprehensive methodology was developed for the identification of glycosides in FG and FGG. A total of 265 glycosides were identified or putatively identified, achieving high coverage of glycoside profiling. Among the 265 glycosides, 219 have not previously been reported in FG or FGG, indicating that this method holds significant potential for identifying previously unreported glycosides. Statistical analysis revealed significant differences in 21 glycosides between FG and FGG. Molecular docking analysis indicated possible interactions between these glycosides and iNOS, suggesting potential relevance to anti-inflammatory processes. Cellular assays further showed that mauritianin and tortoside A exhibited anti-inflammatory effects under the tested conditions *in vitro*. Overall, this study provides methodological support for comprehensive glycoside profiling and suggests phytochemical differences associated with the distinct anti-inflammatory properties of FG and FGG, thereby offering a foundation for their application in the food industries.

## CRediT authorship contribution statement

Xiaoyu Xie: Methodology, Resources, Formal analysis, Investigation, Funding acquisition. Ruonan Zhang: Visualization, Validation, Writing-Original Draft. Xueqin Yin: Formal analysis. Zhangyang Shen: Validation. Chuntao Zeng: Conceptualization. Xiuqiong Zhang: Writing-Review & Editing, Funding acquisition. Weidong Dai: Writing-Review & Editing, Supervision, Funding acquisition.

## Declaration of competing interest

The authors declare that they have no known competing financial interests or personal relationships that could have appeared to influence the work reported in this paper.

## Data Availability

Data will be made available on request.
