# Supplementary material for: Comprehensive glycoside identification and anti-inflammatory activity screening in Fructus Gardeniae and Fructus Gardeniae Grandiflorae
Source: Food Chem X. 2026 May 20;36:104006. doi: 10.1016/j.fochx.2026.104006 (PMC13226894; doi:10.1016/j.fochx.2026.104006)
Supplement: Supplementary material [file mmc1.docx]

Comprehensive identification and anti-inflammatory activity analysis of glycosides in Fructus Gardeniae and Fructus Gardeniae Grandiflorae

Xiaoyu Xie^a^, Ruonan Zhang^a^, Xueqin Yin^a^, Zhangyang Shen^a^, Chuntao Zeng^a^, Xiuqiong Zhang^c*^, Weidong Dai^b*^

^a^ Department of Pharmacy, Jiangxi University of Chinese Medicine, Nanchang 330004, China

^b^ Tea Research Institute, Chinese Academy of Agricultural Sciences, Hangzhou, Zhejiang 310008, China

^c^ College of Chemistry and Chemical Engineering, Yunnan Normal University, Kunming 650500, China

* Corresponding authors:

*Dr. Xiuqiong Zhang, College of Chemistry and Chemical Engineering, Yunnan Normal University, Kunming 650500, China. E-mail: zhangxiuqiong777@163.com*

*Prof. Weidong Dai, State Key Laboratory of Tea Plant Germplasm Innovation and Resource Utilization, Tea Research Institute, Chinese Academy of Agricultural Sciences, Hangzhou, Zhejiang 310008, China. E-mail: daiweidong@tricaas.com*

**The supporting information includes the following items:**

Fig. S1. Effects of MS conditions on the the intensity of these neutral loss peaks

Fig. S2. The chromatogram and mass spectrum of 12 glycosides confirmed with authentic standards

Fig. S3. Molecular docking validation

Table S1. Experimental design matrix and the responses

Table S2. ANOVA results for the response surface model

Table S3. The structural information of 63 monosaccharides

Table S4. The detailed information of glycosyl neutral losses database

Table S5. The glycoside in QC sample of FG and FGG

Table S6. VIP, FC, and FDR values of 26 glycosides with significant changes

Table S7. The binding energy between 15 glycosides and the iNOS protein


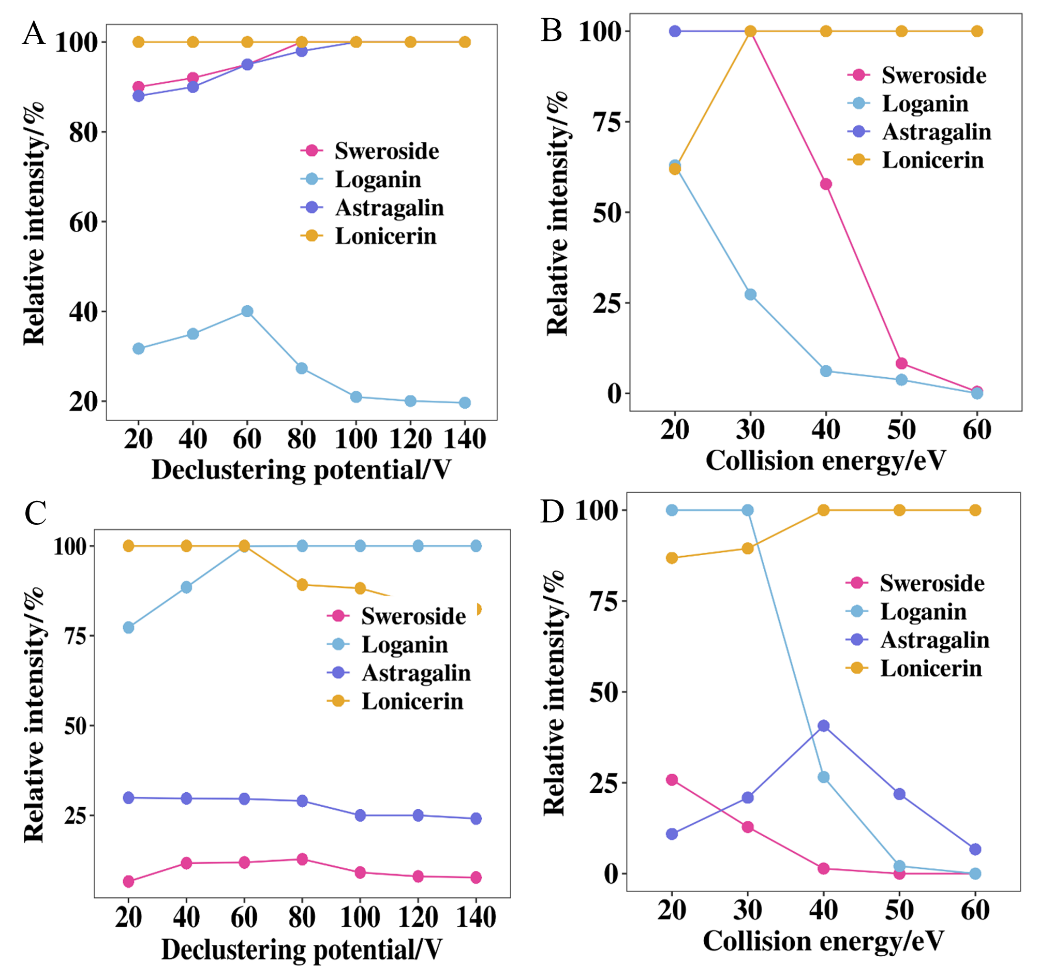


Fig. S1. Effects of MS conditions on the the intensity of these neutral loss peaks. (A) DE-Pos. (B) CE-Pos. (C) DP-Neg. (D) CE-Neg.


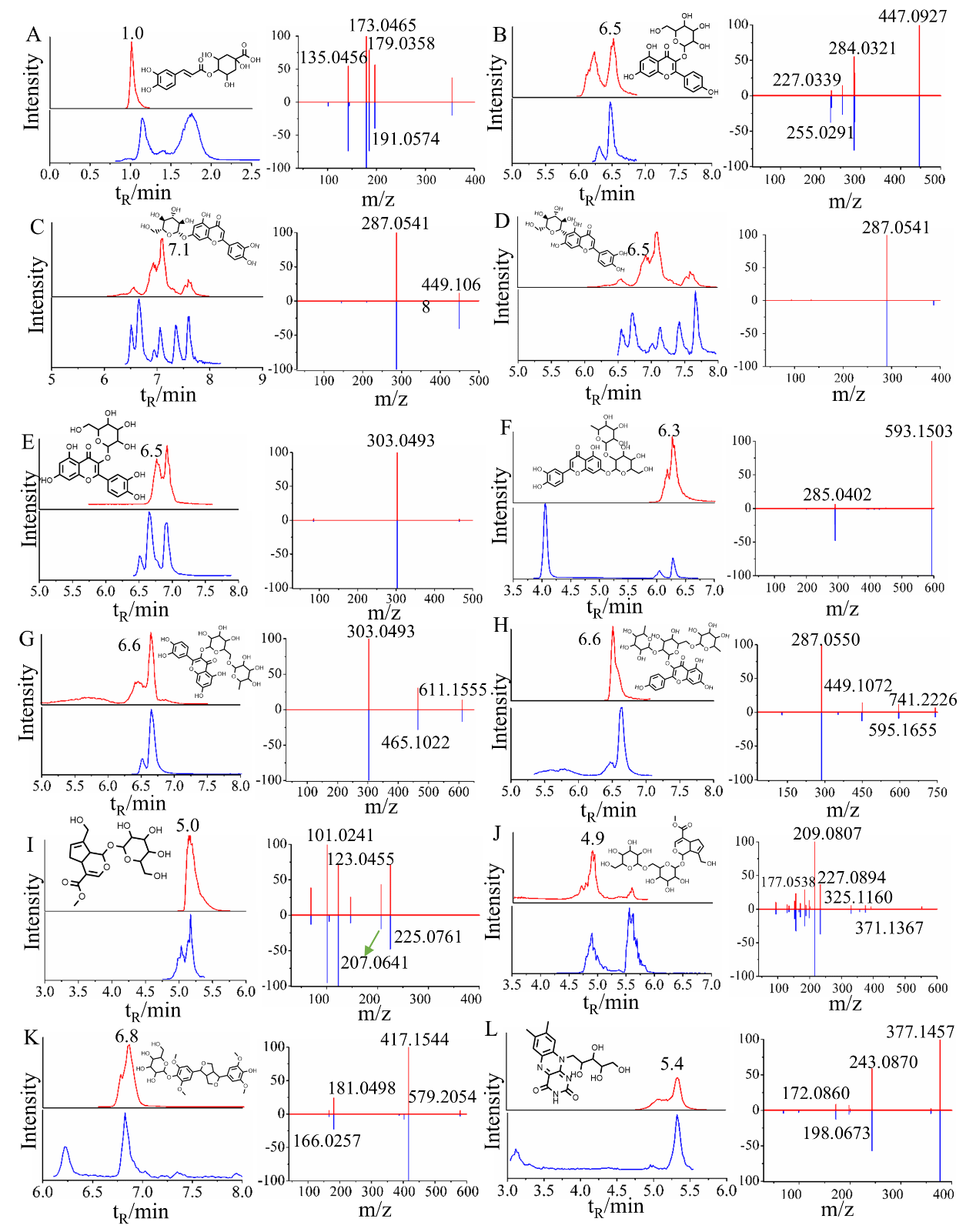


Fig. S2. The chromatogram and mass spectrum of compounds. The compounds A-L are 4-o-trans-caffeoylquinic acid, astragaline, luteoloside, isoorientin, isoquercitrin, lonicerin, vitamin P, mauritianin, geniposide, genipin 1-gentiobioside, tortoside A, and riboflavin (B2), respectively. The upper layer are the authentic standards, while the lower layer are the samples.


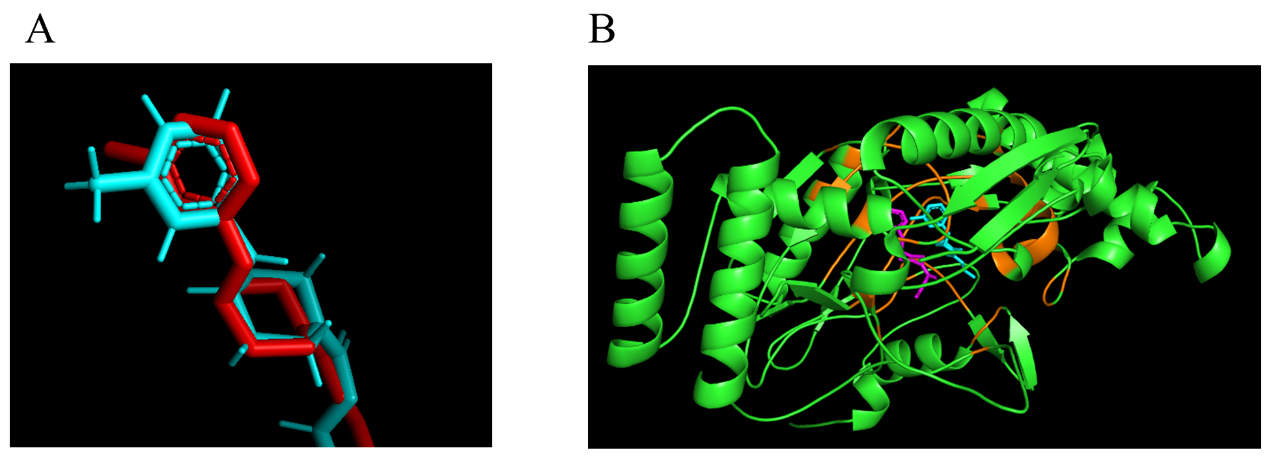


Fig. S3. Molecular docking validation. (A) re-docking the co-crystallized ligand into the active pocket of 3E7G. (B) docking poses of randomly selected glycoside (tortoside A) and the co-crystallized ligand.

Table S1. Experimental design matrix and the responses.

| No | Solvent/% | Time/min | Weight/mg | Number of identified potential glycosides |
| --- | --- | --- | --- | --- |
| 1 | 100 | 40 | 10 | 2536 |
| 2 | 50 | 40 | 45 | 6073 |
| 3 | 100 | 60 | 45 | 4261 |
| 4 | 100 | 40 | 80 | 5256 |
| 5 | 0 | 40 | 10 | 4104 |
| 6 | 50 | 40 | 45 | 6277 |
| 7 | 0 | 60 | 45 | 5884 |
| 8 | 50 | 40 | 45 | 6126 |
| 9 | 50 | 20 | 10 | 4033 |
| 10 | 0 | 40 | 80 | 6211 |
| 11 | 0 | 20 | 45 | 5673 |
| 12 | 50 | 40 | 45 | 6154 |
| 13 | 50 | 60 | 10 | 4157 |
| 14 | 100 | 20 | 45 | 4273 |
| 15 | 50 | 60 | 80 | 6943 |
| 16 | 50 | 20 | 80 | 6897 |
| 17 | 50 | 40 | 45 | 6340 |

Table S2. ANOVA results for the response surface model

| Source | F-value | *P*-value |  |
| --- | --- | --- | --- |
| Model | 115.36 | < 0.0001 | Significant |
| A-Solvent | 163.02 | < 0.0001 |  |
| B-Time | 0.72 | 0.4237 |  |
| C-Weight | 581.78 | < 0.0001 |  |
| AB | 0.53 | 0.4914 |  |
| AC | 3.98 | 0.0862 |  |
| BC | 0.06 | 0.8068 |  |
| A² | 206.70 | < 0.0001 |  |
| B² | 1.62 | 0.2438 |  |
| C² | 62.41 | < 0.0001 |  |
| Lack of Fit | 3.15 | 0.1482 | Not significant |
| Predicted R² |  |  | 0.92 |
| Adjusted R² |  |  | 0.98 |
| Adeq precision |  |  | 38 |
| C.V. % |  |  | 2.86 |

* A, B and C represent the percentage of ethanol in extraction solvent, ultrasonic time, and the weight of sample respectively.

Table S3 The detailed information of monosaccharides

| No. | Name | Formula | MW |
| --- | --- | --- | --- |
| 1 | 5-(Hydroxymethyl) Oxolan-2-Ol | C5H10O3 | 118.0630 |
| 2 | Oxane-2,4,5-Triol | C5H10O4 | 134.0579 |
| 3 | Oxane-3,4,5-Triol | C5H10O4 | 134.0579 |
| 4 | 6-Methyloxane-2,4,5-Triol | C6H12O4 | 148.0736 |
| 5 | Oxane-2,3,4,5-Tetrol | C5H10O5 | 150.0528 |
| 6 | 5-(Hydroxymethyl)Oxolane-2,3,4-Triol | C5H10O5 | 150.0528 |
| 7 | 4-(Hydroxymethyl)Oxolane-2,3,4-Triol | C5H10O5 | 150.0528 |
| 8 | 4,5,6-Trihydroxy-2-Methyloxan-3-One | C6H10O5 | 162.0528 |
| 9 | 6-Methyloxane-2,3,4,5-Tetrol | C6H12O5 | 164.0685 |
| 10 | 5-(1-Hydroxyethyl)Oxolane-2,3,4-Triol | C6H12O5 | 164.0685 |
| 11 | 6-(Hydroxymethyl)Oxane-2,4,5-Triol | C6H12O5 | 164.0685 |
| 12 | 4-Methyloxane-2,3,4,5-Tetrol | C6H12O5 | 164.0685 |
| 13 | 4-Methyltetrahydro-2H-Pyran-2,3,5,6-Tetraol | C6H12O5 | 164.0685 |
| 14 | Oxane-2,3,4,5,6-Pentol | C5H10O6 | 166.0477 |
| 15 | 2,3,5-Trihydroxy-6-(Hydroxymethyl)Oxan-4-One | C6H10O6 | 178.0477 |
| 16 | 6-(Hydroxymethyl)-5-Methyloxane-2,3,4-Triol | C7H14O5 | 178.0841 |
| 17 | 6-Ethyloxane-2,3,4,5-Tetrol | C7H14O5 | 178.0841 |
| 18 | 5-Methoxy-6-Methyloxane-2,3,4-Triol | C7H14O5 | 178.0841 |
| 19 | 4-Methoxy-6-Methyloxane-2,3,5-Triol | C7H14O5 | 178.0841 |
| 20 | 6-(Hydroxymethyl)Oxane-2,3,4,5-Tetrol | C6H12O6 | 180.0634 |
| 21 | 6-Methoxyoxane-2,3,4,5-Tetrol | C6H12O6 | 180.0634 |
| 22 | 2-(Hydroxymethyl)Oxane-2,3,4,5-Tetrol | C6H12O6 | 180.0634 |
| 23 | 5-(1,2-Dihydroxyethyl)Oxolane-2,3,4-Triol | C6H12O6 | 180.0634 |
| 24 | (4,5-Dihydroxy-2-Methyloxan-3-Yl) Acetate | C8H14O5 | 190.0841 |
| 25 | 6-Prop-1-En-2-Yloxane-2,3,4,5-Tetrol | C8H14O5 | 190.0841 |
| 26 | (3,4,5-Trihydroxyoxolan-2-Yl)Methyl Acetate | C7H12O6 | 192.0634 |
| 27 | [4,5-Dihydroxy-2-(Hydroxymethyl)Oxolan-3-Yl] Acetate | C7H12O6 | 192.0634 |
| 28 | (2,3,5-Trihydroxyoxan-4-Yl) Acetate | C7H12O6 | 192.0634 |
| 29 | 3,4,5,6-Tetrahydroxyoxane-2-Carboxamide | C6H11NO6 | 193.0586 |
| 30 | 3,4,5,6-Tetrahydroxyoxane-2-Carboxylic Acid | C6H10O7 | 194.0427 |
| 31 | 6-(Hydroxymethyl)-5-Methoxyoxane-2,3,4-Triol | C7H14O6 | 194.0790 |
| 32 | 6-(Hydroxymethyl)Oxane-2,2,3,4,5-Pentol | C6H12O7 | 196.0583 |
| 33 | 2,2,6-Trimethyl-4,6,7,7A-Tetrahydro-3Ah-[1,3]Dioxolo[4,5-C]Pyran-4,7-Diol | C9H16O5 | 204.0998 |
| 34 | (4,5,6-Trihydroxy-2-Methyloxan-3-Yl) Acetate | C8H14O6 | 206.0790 |
| 35 | (2,4,5-Trihydroxy-6-Methyloxan-3-Yl) Acetate | C8H14O6 | 206.0790 |
| 36 | 6-Butyloxane-2,3,4,5-Tetrol | C9H18O5 | 206.1154 |
| 37 | Methyl 3,4,5,6-Tetrahydroxyoxane-2-Carboxylate | C7H12O7 | 208.0583 |
| 38 | N-[2,4,5-Trihydroxy-6-(Hydroxymethyl)Oxan-3-Yl]Acetamide | C8H15NO6 | 221.0899 |
| 39 | [2,4,5-Trihydroxy-6-(Hydroxymethyl)Oxan-3-Yl] Acetate | C8H14O7 | 222.0740 |
| 40 | (3,4,5,6-Tetrahydroxyoxan-2-Yl)Methyl Acetate | C8H14O7 | 222.0740 |
| 41 | Ethyl 3,4,5,6-Tetrahydroxyoxane-2-Carboxylate | C8H14O7 | 222.0740 |
| 42 | [4,5,6-Trihydroxy-2-(Hydroxymethyl)Oxan-3-Yl] Acetate | C8H14O7 | 222.0740 |
| 43 | [2,3,5-Trihydroxy-6-(Hydroxymethyl)Oxan-4-Yl] Acetate | C8H14O7 | 222.0740 |
| 44 | (3-Acetyloxy-4,5-Dihydroxyoxolan-2-Yl)Methyl Acetate | C9H14O7 | 234.0740 |
| 45 | 5-Acetyloxy-3,4,6-Trihydroxyoxane-2-Carboxylic Acid | C8H12O8 | 236.0532 |
| 46 | 6-(Hydroxymethyl)-3,4,5,6-Tetramethyloxane-2,3,4,5-Tetrol | C10H20O6 | 236.1260 |
| 47 | (5-Acetyloxy-4,6-Dihydroxy-2-Methyloxan-3-Yl) Acetate | C10H16O7 | 248.0896 |
| 48 | (3-Acetyloxy-2,5-Dihydroxy-6-Methyloxan-4-Yl) Acetate | C10H16O7 | 248.0896 |
| 49 | (4-Acetyloxy-5,6-Dihydroxy-2-Methyloxan-3-Yl) Acetate | C10H16O7 | 248.0896 |
| 50 | Butyl 3,4,5,6-Tetrahydroxyoxane-2-Carboxylate | C10H18O7 | 250.1053 |
| 51 | (3,4,5,6-Tetrahydroxyoxan-2-Yl)Methyl 2-Methylpropanoate | C10H18O7 | 250.1053 |
| 52 | (4-Acetyloxy-3,5,6-Trihydroxyoxan-2-Yl)Methyl Acetate | C10H16O8 | 264.0845 |
| 53 | 2,4,5-Trihydroxy-6-(Hydroxymethyl)Tetrahydro-2H-Pyran-3-Yl 2-Methylbutanoate | C11H20O7 | 264.1209 |
| 54 | (3,4,5,6-Tetrahydroxyoxan-2-Yl)Methyl 3-Methylbutanoate | C11H20O7 | 264.1209 |
| 55 | 3-Oxo-3-[(3,4,5,6-Tetrahydroxyoxan-2-Yl)Methoxy]Propanoic Acid | C9H14O9 | 266.0638 |
| 56 | (4,5-Diacetyloxy-6-Hydroxyoxan-3-Yl) Acetate | C11H16O8 | 276.0845 |
| 57 | 4-Oxo-4-[(3,4,5,6-Tetrahydroxyoxan-2-Yl)Methoxy]Butanoic Acid | C10H16O9 | 280.0794 |
| 58 | 6-(1,2,3,4-Tetrahydroxypentyl)Oxane-2,3,4,5-Tetrol | C10H20O9 | 284.1107 |
| 59 | (4,5-Diacetyloxy-6-Hydroxy-2-Methyloxan-3-Yl) Acetate | C12H18O8 | 290.1002 |
| 60 | (3,4-Diacetyloxy-5,6-Dihydroxyoxan-2-Yl)Methyl Acetate | C12H18O9 | 306.0951 |
| 61 | [4,5-Diacetyloxy-6-Hydroxy-2-(Hydroxymethyl)Oxan-3-Yl] Acetate | C12H18O9 | 306.0951 |
| 62 | 3-Hydroxy-3-Methyl-5-Oxo-5-[(3,4,5,6-Tetrahydroxyoxan-2-Yl)Methoxy]Pentanoic Acid | C12H20O10 | 324.1056 |
| 63 | (3,4,5-Triacetyloxy-6-Hydroxyoxan-2-Yl)Methyl Acetate | C14H20O10 | 348.1056 |

Table S4 The detailed information of glycosyl neutral losses database

| Monosaccharide | Name1 | Name 2 | Name 3 | Name 4 | Modification | Neutral-loss 1 | Neutral-loss 2 |
| --- | --- | --- | --- | --- | --- | --- | --- |
|  | 5-(Hydroxymethyl)Oxolan-2-Ol |  |  |  | No | 118.0630 | 100.0474 |
|  | Oxane-2,4,5-Triol |  |  |  | No | 134.0579 | 116.0423 |
|  | Oxane-3,4,5-Triol |  |  |  | No | 134.0579 | 116.0423 |
|  | 6-Methyloxane-2,4,5-Triol |  |  |  | No | 148.0736 | 130.0580 |
|  | Oxane-2,3,4,5-Tetrol |  |  |  | No | 150.0528 | 132.0372 |
|  | 5-(Hydroxymethyl)Oxolane-2,3,4-Triol |  |  |  | No | 150.0528 | 132.0372 |
|  | 4-(Hydroxymethyl)Oxolane-2,3,4-Triol |  |  |  | No | 150.0528 | 132.0372 |
|  | 4,5,6-Trihydroxy-2-Methyloxan-3-One |  |  |  | No | 162.0528 | 144.0372 |
|  | 6-Methyloxane-2,3,4,5-Tetrol |  |  |  | No | 164.0685 | 146.0529 |
|  | 5-(1-Hydroxyethyl)Oxolane-2,3,4-Triol |  |  |  | No | 164.0685 | 146.0529 |
|  | 6-(Hydroxymethyl)Oxane-2,4,5-Triol |  |  |  | No | 164.0685 | 146.0529 |
|  | 4-Methyloxane-2,3,4,5-Tetrol |  |  |  | No | 164.0685 | 146.0529 |
|  | 4-Methyltetrahydro-2H-Pyran-2,3,5,6-Tetraol |  |  |  | No | 164.0685 | 146.0529 |
|  | Oxane-2,3,4,5,6-Pentol |  |  |  | No | 166.0477 | 148.0321 |
|  | 2,3,5-Trihydroxy-6-(Hydroxymethyl)Oxan-4-One |  |  |  | No | 178.0477 | 160.0321 |
|  | 6-(Hydroxymethyl)-5-Methyloxane-2,3,4-Triol |  |  |  | No | 178.0841 | 160.0685 |
|  | 6-Ethyloxane-2,3,4,5-Tetrol |  |  |  | No | 178.0841 | 160.0685 |
|  | 5-Methoxy-6-Methyloxane-2,3,4-Triol |  |  |  | No | 178.0841 | 160.0685 |
|  | 4-Methoxy-6-Methyloxane-2,3,5-Triol |  |  |  | No | 178.0841 | 160.0685 |
|  | 6-(Hydroxymethyl)Oxane-2,3,4,5-Tetrol |  |  |  | No | 180.0634 | 162.0478 |
|  | 6-Methoxyoxane-2,3,4,5-Tetrol |  |  |  | No | 180.0634 | 162.0478 |
|  | 2-(Hydroxymethyl)Oxane-2,3,4,5-Tetrol |  |  |  | No | 180.0634 | 162.0478 |
|  | 5-(1,2-Dihydroxyethyl)Oxolane-2,3,4-Triol |  |  |  | No | 180.0634 | 162.0478 |
|  | (4,5-Dihydroxy-2-Methyloxan-3-Yl) Acetate |  |  |  | No | 190.0841 | 172.0685 |
|  | 6-Prop-1-En-2-Yloxane-2,3,4,5-Tetrol |  |  |  | No | 190.0841 | 172.0685 |
|  | (3,4,5-Trihydroxyoxolan-2-Yl)Methyl Acetate |  |  |  | No | 192.0634 | 174.0478 |
|  | [4,5-Dihydroxy-2-(Hydroxymethyl)Oxolan-3-Yl] Acetate |  |  |  | No | 192.0634 | 174.0478 |
|  | (2,3,5-Trihydroxyoxan-4-Yl) Acetate |  |  |  | No | 192.0634 | 174.0478 |
|  | 3,4,5,6-Tetrahydroxyoxane-2-Carboxamide |  |  |  | No | 193.0586 | 175.0430 |
|  | 3,4,5,6-Tetrahydroxyoxane-2-Carboxylic Acid |  |  |  | No | 194.0427 | 176.0271 |
|  | 6-(Hydroxymethyl)-5-Methoxyoxane-2,3,4-Triol |  |  |  | No | 194.0790 | 176.0634 |
|  | 6-(Hydroxymethyl)Oxane-2,2,3,4,5-Pentol |  |  |  | No | 196.0583 | 178.0427 |
|  | 2,2,6-Trimethyl-4,6,7,7A-Tetrahydro-3Ah-[1,3]Dioxolo[4,5-C]Pyran-4,7-Diol |  |  |  | No | 204.0998 | 186.0842 |
|  | (4,5,6-Trihydroxy-2-Methyloxan-3-Yl) Acetate |  |  |  | No | 206.0790 | 188.0634 |
|  | (2,4,5-Trihydroxy-6-Methyloxan-3-Yl) Acetate |  |  |  | No | 206.0790 | 188.0634 |
|  | 6-Butyloxane-2,3,4,5-Tetrol |  |  |  | No | 206.1154 | 188.0998 |
|  | Methyl 3,4,5,6-Tetrahydroxyoxane-2-Carboxylate |  |  |  | No | 208.0583 | 190.0427 |
|  | N-[2,4,5-Trihydroxy-6-(Hydroxymethyl)Oxan-3-Yl]Acetamide |  |  |  | No | 221.0899 | 203.0743 |
|  | [2,4,5-Trihydroxy-6-(Hydroxymethyl)Oxan-3-Yl] Acetate |  |  |  | No | 222.0740 | 204.0584 |
|  | (3,4,5,6-Tetrahydroxyoxan-2-Yl)Methyl Acetate |  |  |  | No | 222.0740 | 204.0584 |
|  | Ethyl 3,4,5,6-Tetrahydroxyoxane-2-Carboxylate |  |  |  | No | 222.0740 | 204.0584 |
|  | [4,5,6-Trihydroxy-2-(Hydroxymethyl)Oxan-3-Yl] Acetate |  |  |  | No | 222.0740 | 204.0584 |
|  | [2,3,5-Trihydroxy-6-(Hydroxymethyl)Oxan-4-Yl] Acetate |  |  |  | No | 222.0740 | 204.0584 |
|  | (3-Acetyloxy-4,5-Dihydroxyoxolan-2-Yl)Methyl Acetate |  |  |  | No | 234.0740 | 216.0584 |
|  | 5-Acetyloxy-3,4,6-Trihydroxyoxane-2-Carboxylic Acid |  |  |  | No | 236.0532 | 218.0376 |
|  | 6-(Hydroxymethyl)-3,4,5,6-Tetramethyloxane-2,3,4,5-Tetrol |  |  |  | No | 236.1260 | 218.1104 |
|  | (5-Acetyloxy-4,6-Dihydroxy-2-Methyloxan-3-Yl) Acetate |  |  |  | No | 248.0896 | 230.0740 |
|  | (3-Acetyloxy-2,5-Dihydroxy-6-Methyloxan-4-Yl) Acetate |  |  |  | No | 248.0896 | 230.0740 |
|  | (4-Acetyloxy-5,6-Dihydroxy-2-Methyloxan-3-Yl) Acetate |  |  |  | No | 248.0896 | 230.0740 |
|  | Butyl 3,4,5,6-Tetrahydroxyoxane-2-Carboxylate |  |  |  | No | 250.1053 | 232.0897 |
|  | (3,4,5,6-Tetrahydroxyoxan-2-Yl)Methyl 2-Methylpropanoate |  |  |  | No | 250.1053 | 232.0897 |
|  | (4-Acetyloxy-3,5,6-Trihydroxyoxan-2-Yl)Methyl Acetate |  |  |  | No | 264.0845 | 246.0689 |
|  | 2,4,5-Trihydroxy-6-(Hydroxymethyl)Tetrahydro-2H-Pyran-3-Yl 2-Methylbutanoate |  |  |  | No | 264.1209 | 246.1053 |
|  | (3,4,5,6-Tetrahydroxyoxan-2-Yl)Methyl 3-Methylbutanoate |  |  |  | No | 264.1209 | 246.1053 |
|  | 3-Oxo-3-[(3,4,5,6-Tetrahydroxyoxan-2-Yl)Methoxy]Propanoic Acid |  |  |  | No | 266.0638 | 248.0482 |
|  | (4,5-Diacetyloxy-6-Hydroxyoxan-3-Yl) Acetate |  |  |  | No | 276.0845 | 258.0689 |
|  | 4-Oxo-4-[(3,4,5,6-Tetrahydroxyoxan-2-Yl)Methoxy]Butanoic Acid |  |  |  | No | 280.0794 | 262.0638 |
|  | 6-(1,2,3,4-Tetrahydroxypentyl)Oxane-2,3,4,5-Tetrol |  |  |  | No | 284.1107 | 266.0951 |
|  | (4,5-Diacetyloxy-6-Hydroxy-2-Methyloxan-3-Yl) Acetate |  |  |  | No | 290.1002 | 272.0846 |
|  | (3,4-Diacetyloxy-5,6-Dihydroxyoxan-2-Yl)Methyl Acetate |  |  |  | No | 306.0951 | 288.0795 |
|  | [4,5-Diacetyloxy-6-Hydroxy-2-(Hydroxymethyl)Oxan-3-Yl] Acetate |  |  |  | No | 306.0951 | 288.0795 |
|  | 3-Hydroxy-3-Methyl-5-Oxo-5-[(3,4,5,6-Tetrahydroxyoxan-2-Yl)Methoxy]Pentanoic Acid |  |  |  | No | 324.1056 | 306.0900 |
|  | (3,4,5-Triacetyloxy-6-Hydroxyoxan-2-Yl)Methyl Acetate |  |  |  | No | 348.1056 | 330.0900 |
|  | 6-Methyloxane-2,3,4,5-Tetrol |  |  |  | One Acetylation | 206.0786 | 188.0630 |
|  | 6-Methyloxane-2,3,4,5-Tetrol |  |  |  | Two Acetylation | 248.0888 | 230.0732 |
|  | 6-Methyloxane-2,3,4,5-Tetrol |  |  |  | Three Acetylation | 290.0989 | 272.0833 |
|  | 6-Methyloxane-2,3,4,5-Tetrol |  |  |  | One Methylation | 178.0841 | 160.0685 |
|  | 6-Methyloxane-2,3,4,5-Tetrol |  |  |  | Two Methylation | 192.0997 | 174.0841 |
|  | 6-Methyloxane-2,3,4,5-Tetrol |  |  |  | Three Methylation | 206.1153 | 188.0997 |
|  | 6-Methyloxane-2,3,4,5-Tetrol |  |  |  | Four Methylation | 220.1309 | 202.1153 |
|  | Oxane-2,3,4,5-Tetrol |  |  |  | Two Acetylation | 232.0731 | 214.0575 |
|  | Oxane-2,3,4,5-Tetrol |  |  |  | Two Methylation | 178.0840 | 160.0684 |
|  | Oxane-2,3,4,5-Tetrol |  |  |  | Three Methylation | 192.0996 | 174.0840 |
|  | Oxane-2,3,4,5-Tetrol |  |  |  | Four Methylation | 206.1152 | 188.0996 |
|  | 6-(Hydroxymethyl)Oxane-2,3,4,5-Tetrol |  |  |  | Two Methylation | 208.0946 | 190.0790 |
|  | 6-(Hydroxymethyl)Oxane-2,3,4,5-Tetrol |  |  |  | Three Methylation | 222.1102 | 204.0946 |
|  | 5-(Hydroxymethyl)Oxolane-2,3,4-Triol |  |  |  | One Acetylation | 191.0630 | 173.0474 |
|  | 5-(Hydroxymethyl)Oxolane-2,3,4-Triol |  |  |  | Two Acetylation | 232.0731 | 214.0575 |
|  | 5-(Hydroxymethyl)Oxolane-2,3,4-Triol |  |  |  | Three Acetylation | 273.0833 | 255.0677 |
|  | 5-(Hydroxymethyl)Oxolane-2,3,4-Triol |  |  |  | One Methylation | 164.0684 | 146.0528 |
|  | 5-(Hydroxymethyl)Oxolane-2,3,4-Triol |  |  |  | Two Methylation | 178.0840 | 160.0684 |
|  | 5-(Hydroxymethyl)Oxolane-2,3,4-Triol |  |  |  | Three Methylation | 192.0996 | 174.0840 |
|  | Oxane-2,3,4,5,6-Pentol |  |  |  | One Acetylation | 207.0579 | 189.0423 |
|  | Oxane-2,3,4,5,6-Pentol |  |  |  | Two Acetylation | 248.0680 | 230.0524 |
|  | Oxane-2,3,4,5,6-Pentol |  |  |  | Three Acetylation | 289.0782 | 271.0626 |
|  | Oxane-2,3,4,5,6-Pentol |  |  |  | Three Methylation | 208.0945 | 190.0789 |
|  | Oxane-2,3,4,5,6-Pentol |  |  |  | Four Methylation | 222.1101 | 204.0945 |
|  | 3,4,5,6-Tetrahydroxyoxane-2-Carboxylic Acid |  |  |  | Two Acetylation | 276.0630 | 258.0474 |
|  | 3,4,5,6-Tetrahydroxyoxane-2-Carboxylic Acid |  |  |  | Three Acetylation | 317.0731 | 299.0575 |
|  | 3,4,5,6-Tetrahydroxyoxane-2-Carboxylic Acid |  |  |  | Two Methylation | 222.0739 | 204.0583 |
|  | 3,4,5,6-Tetrahydroxyoxane-2-Carboxylic Acid |  |  |  | Three Methylation | 236.0895 | 218.0739 |
|  | 3,4,5,6-Tetrahydroxyoxane-2-Carboxylic Acid |  |  |  | Four Methylation | 250.1051 | 232.0895 |
| Disaccharide | 6-Methyloxane-2,3,4,5-Tetrol | 6-Methyloxane-2,3,4,5-Tetrol |  |  | Polymerization | 310.1213 | 292.1057 |
|  | 6-Methyloxane-2,3,4,5-Tetrol | Oxane-2,3,4,5-Tetrol |  |  | Polymerization | 296.1057 | 278.0901 |
|  | 6-Methyloxane-2,3,4,5-Tetrol | 6-(Hydroxymethyl)Oxane-2,3,4,5-Tetrol |  |  | Polymerization | 326.1163 | 308.1007 |
|  | 6-Methyloxane-2,3,4,5-Tetrol | 5-(Hydroxymethyl)Oxolane-2,3,4-Triol |  |  | Polymerization | 296.1057 | 278.0901 |
|  | 6-Methyloxane-2,3,4,5-Tetrol | Oxane-2,3,4,5,6-Pentol |  |  | Polymerization | 312.1006 | 294.0850 |
|  | 6-Methyloxane-2,3,4,5-Tetrol | 3,4,5,6-Tetrahydroxyoxane-2-Carboxylic Acid |  |  | Polymerization | 340.0955 | 322.0799 |
|  | Oxane-2,3,4,5-Tetrol | Oxane-2,3,4,5-Tetrol |  |  | Polymerization | 282.0900 | 264.0744 |
|  | Oxane-2,3,4,5-Tetrol | 6-(Hydroxymethyl)Oxane-2,3,4,5-Tetrol |  |  | Polymerization | 312.1006 | 294.0850 |
|  | Oxane-2,3,4,5-Tetrol | 5-(Hydroxymethyl)Oxolane-2,3,4-Triol |  |  | Polymerization | 282.0900 | 264.0744 |
|  | Oxane-2,3,4,5-Tetrol | Oxane-2,3,4,5,6-Pentol |  |  | Polymerization | 298.0850 | 280.0694 |
|  | Oxane-2,3,4,5-Tetrol | 3,4,5,6-Tetrahydroxyoxane-2-Carboxylic Acid |  |  | Polymerization | 326.0799 | 308.0643 |
|  | 6-(Hydroxymethyl)Oxane-2,3,4,5-Tetrol | 6-(Hydroxymethyl)Oxane-2,3,4,5-Tetrol |  |  | Polymerization | 342.1112 | 324.0956 |
|  | 6-(Hydroxymethyl)Oxane-2,3,4,5-Tetrol | 5-(Hydroxymethyl)Oxolane-2,3,4-Triol |  |  | Polymerization | 312.1006 | 294.0850 |
|  | 6-(Hydroxymethyl)Oxane-2,3,4,5-Tetrol | Oxane-2,3,4,5,6-Pentol |  |  | Polymerization | 328.0955 | 310.0799 |
|  | 6-(Hydroxymethyl)Oxane-2,3,4,5-Tetrol | 3,4,5,6-Tetrahydroxyoxane-2-Carboxylic Acid |  |  | Polymerization | 356.0904 | 338.0748 |
|  | 5-(Hydroxymethyl)Oxolane-2,3,4-Triol | 5-(Hydroxymethyl)Oxolane-2,3,4-Triol |  |  | Polymerization | 282.0900 | 264.0744 |
|  | 5-(Hydroxymethyl)Oxolane-2,3,4-Triol | Oxane-2,3,4,5,6-Pentol |  |  | Polymerization | 298.0850 | 280.0694 |
|  | 5-(Hydroxymethyl)Oxolane-2,3,4-Triol | 3,4,5,6-Tetrahydroxyoxane-2-Carboxylic Acid |  |  | Polymerization | 326.0799 | 308.0643 |
|  | Oxane-2,3,4,5,6-Pentol | Oxane-2,3,4,5,6-Pentol |  |  | Polymerization | 314.0799 | 296.0643 |
|  | Oxane-2,3,4,5,6-Pentol | 3,4,5,6-Tetrahydroxyoxane-2-Carboxylic Acid |  |  | Polymerization | 342.0748 | 324.0592 |
|  | 3,4,5,6-Tetrahydroxyoxane-2-Carboxylic Acid | 3,4,5,6-Tetrahydroxyoxane-2-Carboxylic Acid |  |  | Polymerization | 370.0697 | 352.0541 |
| Trisaccharide | 6-Methyloxane-2,3,4,5-Tetrol | 6-Methyloxane-2,3,4,5-Tetrol | 6-Methyloxane-2,3,4,5-Tetrol |  | Polymerization | 456.1742 | 438.1586 |
|  | 6-Methyloxane-2,3,4,5-Tetrol | 6-Methyloxane-2,3,4,5-Tetrol | Oxane-2,3,4,5-Tetrol |  | Polymerization | 442.1586 | 424.1430 |
|  | 6-Methyloxane-2,3,4,5-Tetrol | 6-Methyloxane-2,3,4,5-Tetrol | 6-(Hydroxymethyl)Oxane-2,3,4,5-Tetrol |  | Polymerization | 472.1691 | 454.1535 |
|  | 6-Methyloxane-2,3,4,5-Tetrol | 6-Methyloxane-2,3,4,5-Tetrol | 5-(Hydroxymethyl)Oxolane-2,3,4-Triol |  | Polymerization | 442.1586 | 424.1430 |
|  | 6-Methyloxane-2,3,4,5-Tetrol | 6-Methyloxane-2,3,4,5-Tetrol | Oxane-2,3,4,5,6-Pentol |  | Polymerization | 458.1535 | 440.1379 |
|  | 6-Methyloxane-2,3,4,5-Tetrol | 6-Methyloxane-2,3,4,5-Tetrol | 3,4,5,6-Tetrahydroxyoxane-2-Carboxylic Acid |  | Polymerization | 486.1484 | 468.1328 |
|  | 6-Methyloxane-2,3,4,5-Tetrol | Oxane-2,3,4,6-Tetrol | Oxane-2,3,4,5-Tetrol |  | Polymerization | 414.1273 | 396.1117 |
|  | 6-Methyloxane-2,3,4,5-Tetrol | Oxane-2,3,4,6-Tetrol | 6-(Hydroxymethyl)Oxane-2,3,4,5-Tetrol |  | Polymerization | 444.1378 | 426.1222 |
|  | 6-Methyloxane-2,3,4,5-Tetrol | Oxane-2,3,4,6-Tetrol | 5-(Hydroxymethyl)Oxolane-2,3,4-Triol |  | Polymerization | 414.1273 | 396.1117 |
|  | 6-Methyloxane-2,3,4,5-Tetrol | Oxane-2,3,4,6-Tetrol | Oxane-2,3,4,5,6-Pentol |  | Polymerization | 430.1222 | 412.1066 |
|  | 6-Methyloxane-2,3,4,5-Tetrol | Oxane-2,3,4,6-Tetrol | 3,4,5,6-Tetrahydroxyoxane-2-Carboxylic Acid |  | Polymerization | 458.1171 | 440.1015 |
|  | 6-Methyloxane-2,3,4,5-Tetrol | 6-(Hydroxymethyl)Oxane-2,3,4,5-Tetrol | 6-(Hydroxymethyl)Oxane-2,3,4,5-Tetrol |  | Polymerization | 504.1590 | 486.1434 |
|  | 6-Methyloxane-2,3,4,5-Tetrol | 6-(Hydroxymethyl)Oxane-2,3,4,5-Tetrol | 5-(Hydroxymethyl)Oxolane-2,3,4-Triol |  | Polymerization | 474.1484 | 456.1328 |
|  | 6-Methyloxane-2,3,4,5-Tetrol | 6-(Hydroxymethyl)Oxane-2,3,4,5-Tetrol | Oxane-2,3,4,5,6-Pentol |  | Polymerization | 490.1433 | 472.1277 |
|  | 6-Methyloxane-2,3,4,5-Tetrol | 6-(Hydroxymethyl)Oxane-2,3,4,5-Tetrol | 3,4,5,6-Tetrahydroxyoxane-2-Carboxylic Acid |  | Polymerization | 518.1382 | 500.1226 |
|  | 6-Methyloxane-2,3,4,5-Tetrol | 5-(Hydroxymethyl)Oxolane-2,3,4-Triol | 5-(Hydroxymethyl)Oxolane-2,3,4-Triol |  | Polymerization | 414.1273 | 396.1117 |
|  | 6-Methyloxane-2,3,4,5-Tetrol | 5-(Hydroxymethyl)Oxolane-2,3,4-Triol | Oxane-2,3,4,5,6-Pentol |  | Polymerization | 430.1222 | 412.1066 |
|  | 6-Methyloxane-2,3,4,5-Tetrol | 5-(Hydroxymethyl)Oxolane-2,3,4-Triol | 3,4,5,6-Tetrahydroxyoxane-2-Carboxylic Acid |  | Polymerization | 458.1171 | 440.1015 |
|  | 6-Methyloxane-2,3,4,5-Tetrol | Oxane-2,3,4,5,6-Pentol | Oxane-2,3,4,5,6-Pentol |  | Polymerization | 462.1120 | 444.0964 |
|  | 6-Methyloxane-2,3,4,5-Tetrol | Oxane-2,3,4,5,6-Pentol | 3,4,5,6-Tetrahydroxyoxane-2-Carboxylic Acid |  | Polymerization | 490.1069 | 472.0913 |
|  | 6-Methyloxane-2,3,4,5-Tetrol | 3,4,5,6-Tetrahydroxyoxane-2-Carboxylic Acid | 3,4,5,6-Tetrahydroxyoxane-2-Carboxylic Acid |  | Polymerization | 546.0968 | 528.0812 |
|  | Oxane-2,3,4,5-Tetrol | Oxane-2,3,4,5-Tetrol | Oxane-2,3,4,5-Tetrol |  | Polymerization | 414.1273 | 396.1117 |
|  | Oxane-2,3,4,5-Tetrol | Oxane-2,3,4,5-Tetrol | 6-(Hydroxymethyl)Oxane-2,3,4,5-Tetrol |  | Polymerization | 444.1378 | 426.1222 |
|  | Oxane-2,3,4,6-Tetrol | Oxane-2,3,4,6-Tetrol | 5-(Hydroxymethyl)Oxolane-2,3,4-Triol |  | Polymerization | 414.1273 | 396.1117 |
|  | Oxane-2,3,4,7-Tetrol | Oxane-2,3,4,7-Tetrol | Oxane-2,3,4,5,6-Pentol |  | Polymerization | 430.1222 | 412.1066 |
|  | Oxane-2,3,4,8-Tetrol | Oxane-2,3,4,8-Tetrol | 3,4,5,6-Tetrahydroxyoxane-2-Carboxylic Acid |  | Polymerization | 458.1171 | 440.1015 |
|  | Oxane-2,3,4,9-Tetrol | 6-(Hydroxymethyl)Oxane-2,3,4,5-Tetrol | 6-(Hydroxymethyl)Oxane-2,3,4,5-Tetrol |  | Polymerization | 474.1484 | 456.1328 |
|  | Oxane-2,3,4,10-Tetrol | 6-(Hydroxymethyl)Oxane-2,3,4,6-Tetrol | 5-(Hydroxymethyl)Oxolane-2,3,4-Triol |  | Polymerization | 444.1378 | 426.1222 |
|  | Oxane-2,3,4,11-Tetrol | 6-(Hydroxymethyl)Oxane-2,3,4,7-Tetrol | Oxane-2,3,4,5,6-Pentol |  | Polymerization | 460.1327 | 442.1171 |
|  | Oxane-2,3,4,12-Tetrol | 6-(Hydroxymethyl)Oxane-2,3,4,8-Tetrol | 3,4,5,6-Tetrahydroxyoxane-2-Carboxylic Acid |  | Polymerization | 488.1277 | 470.1121 |
|  | Oxane-2,3,4,13-Tetrol | 5-(Hydroxymethyl)Oxolane-2,3,4-Triol | 5-(Hydroxymethyl)Oxolane-2,3,4-Triol |  | Polymerization | 414.1273 | 396.1117 |
|  | Oxane-2,3,4,14-Tetrol | 5-(Hydroxymethyl)Oxolane-2,3,5-Triol | Oxane-2,3,4,5,6-Pentol |  | Polymerization | 430.1222 | 412.1066 |
|  | Oxane-2,3,4,15-Tetrol | 5-(Hydroxymethyl)Oxolane-2,3,6-Triol | 3,4,5,6-Tetrahydroxyoxane-2-Carboxylic Acid |  | Polymerization | 458.1171 | 440.1015 |
|  | Oxane-2,3,4,16-Tetrol | Oxane-2,3,4,5,6-Pentol | Oxane-2,3,4,5,6-Pentol |  | Polymerization | 446.1171 | 428.1015 |
|  | Oxane-2,3,4,17-Tetrol | Oxane-2,3,4,5,7-Pentol | 3,4,5,6-Tetrahydroxyoxane-2-Carboxylic Acid |  | Polymerization | 474.1120 | 456.0964 |
|  | Oxane-2,3,4,18-Tetrol | 3,4,5,6-Tetrahydroxyoxane-2-Carboxylic Acid | 3,4,5,6-Tetrahydroxyoxane-2-Carboxylic Acid |  | Polymerization | 502.1069 | 484.0913 |
|  | 6-(Hydroxymethyl)Oxane-2,3,4,5-Tetrol | 6-(Hydroxymethyl)Oxane-2,3,4,5-Tetrol | 6-(Hydroxymethyl)Oxane-2,3,4,5-Tetrol |  | Polymerization | 504.1590 | 486.1434 |
|  | 6-(Hydroxymethyl)Oxane-2,3,4,5-Tetrol | 6-(Hydroxymethyl)Oxane-2,3,4,5-Tetrol | 5-(Hydroxymethyl)Oxolane-2,3,4-Triol |  | Polymerization | 474.1484 | 456.1328 |
|  | 6-(Hydroxymethyl)Oxane-2,3,4,5-Tetrol | 6-(Hydroxymethyl)Oxane-2,3,4,5-Tetrol | Oxane-2,3,4,5,6-Pentol |  | Polymerization | 490.1433 | 472.1277 |
|  | 6-(Hydroxymethyl)Oxane-2,3,4,5-Tetrol | 6-(Hydroxymethyl)Oxane-2,3,4,5-Tetrol | 3,4,5,6-Tetrahydroxyoxane-2-Carboxylic Acid |  | Polymerization | 518.1382 | 500.1226 |
|  | 6-(Hydroxymethyl)Oxane-2,3,4,5-Tetrol | 5-(Hydroxymethyl)Oxolane-2,3,4-Triol | 5-(Hydroxymethyl)Oxolane-2,3,4-Triol |  | Polymerization | 444.1378 | 426.1222 |
|  | 6-(Hydroxymethyl)Oxane-2,3,4,5-Tetrol | 5-(Hydroxymethyl)Oxolane-2,3,4-Triol | Oxane-2,3,4,5,6-Pentol |  | Polymerization | 460.1327 | 442.1171 |
|  | 6-(Hydroxymethyl)Oxane-2,3,4,5-Tetrol | 5-(Hydroxymethyl)Oxolane-2,3,4-Triol | 3,4,5,6-Tetrahydroxyoxane-2-Carboxylic Acid |  | Polymerization | 488.1277 | 470.1121 |
|  | 6-(Hydroxymethyl)Oxane-2,3,4,5-Tetrol | Oxane-2,3,4,5,6-Pentol | Oxane-2,3,4,5,6-Pentol |  | Polymerization | 476.1277 | 458.1121 |
|  | 6-(Hydroxymethyl)Oxane-2,3,4,5-Tetrol | Oxane-2,3,4,5,7-Pentol | 3,4,5,6-Tetrahydroxyoxane-2-Carboxylic Acid |  | Polymerization | 504.1226 | 486.1070 |
|  | 6-(Hydroxymethyl)Oxane-2,3,4,5-Tetrol | 3,4,5,6-Tetrahydroxyoxane-2-Carboxylic Acid | 3,4,5,6-Tetrahydroxyoxane-2-Carboxylic Acid |  | Polymerization | 532.1175 | 514.1019 |
|  | 5-(Hydroxymethyl)Oxolane-2,3,4-Triol | 5-(Hydroxymethyl)Oxolane-2,3,4-Triol | 5-(Hydroxymethyl)Oxolane-2,3,4-Triol |  | Polymerization | 414.1273 | 396.1117 |
|  | 5-(Hydroxymethyl)Oxolane-2,3,4-Triol | 5-(Hydroxymethyl)Oxolane-2,3,4-Triol | Oxane-2,3,4,5,6-Pentol |  | Polymerization | 430.1222 | 412.1066 |
|  | 5-(Hydroxymethyl)Oxolane-2,3,4-Triol | 5-(Hydroxymethyl)Oxolane-2,3,4-Triol | 3,4,5,6-Tetrahydroxyoxane-2-Carboxylic Acid |  | Polymerization | 458.1171 | 440.1015 |
|  | 5-(Hydroxymethyl)Oxolane-2,3,4-Triol | Oxane-2,3,4,5,6-Pentol | Oxane-2,3,4,5,6-Pentol |  | Polymerization | 446.1171 | 428.1015 |
|  | 5-(Hydroxymethyl)Oxolane-2,3,4-Triol | Oxane-2,3,4,5,7-Pentol | 3,4,5,6-Tetrahydroxyoxane-2-Carboxylic Acid |  | Polymerization | 474.1120 | 456.0964 |
|  | 5-(Hydroxymethyl)Oxolane-2,3,4-Triol | 3,4,5,6-Tetrahydroxyoxane-2-Carboxylic Acid | 3,4,5,6-Tetrahydroxyoxane-2-Carboxylic Acid |  | Polymerization | 502.1069 | 484.0913 |
|  | Oxane-2,3,4,5,6-Pentol | Oxane-2,3,4,5,6-Pentol | Oxane-2,3,4,5,6-Pentol |  | Polymerization | 462.1120 | 444.0964 |
|  | Oxane-2,3,4,5,6-Pentol | Oxane-2,3,4,5,6-Pentol | 3,4,5,6-Tetrahydroxyoxane-2-Carboxylic Acid |  | Polymerization | 490.1069 | 472.0913 |
|  | Oxane-2,3,4,5,6-Pentol | 3,4,5,6-Tetrahydroxyoxane-2-Carboxylic Acid | Oxane-2,3,4,5,6-Pentol |  | Polymerization | 490.1069 | 472.0913 |
|  | Oxane-2,3,4,5,6-Pentol | 3,4,5,6-Tetrahydroxyoxane-2-Carboxylic Acid | 3,4,5,6-Tetrahydroxyoxane-2-Carboxylic Acid |  | Polymerization | 518.1018 | 500.0862 |
|  | 3,4,5,6-Tetrahydroxyoxane-2-Carboxylic Acid | 3,4,5,6-Tetrahydroxyoxane-2-Carboxylic Acid | 3,4,5,6-Tetrahydroxyoxane-2-Carboxylic Acid |  | Polymerization | 546.0968 | 528.0812 |
| Tetrasaccharide | 6-Methyloxane-2,3,4,5-Tetrol | 6-Methyloxane-2,3,4,5-Tetrol | 6-Methyloxane-2,3,4,5-Tetrol | 6-Methyloxane-2,3,4,5-Tetrol | Polymerization | 602.2271 | 584.2115 |
|  | 6-Methyloxane-2,3,4,5-Tetrol | 6-Methyloxane-2,3,4,5-Tetrol | 6-Methyloxane-2,3,4,5-Tetrol | Oxane-2,3,4,5-Tetrol | Polymerization | 588.2114 | 570.1958 |
|  | 6-Methyloxane-2,3,4,5-Tetrol | 6-Methyloxane-2,3,4,5-Tetrol | 6-Methyloxane-2,3,4,5-Tetrol | 6-(Hydroxymethyl)Oxane-2,3,4,5-Tetrol | Polymerization | 618.2220 | 600.2064 |
|  | 6-Methyloxane-2,3,4,5-Tetrol | 6-Methyloxane-2,3,4,5-Tetrol | 6-Methyloxane-2,3,4,5-Tetrol | 5-(Hydroxymethyl)Oxolane-2,3,4-Triol | Polymerization | 588.2114 | 570.1958 |
|  | 6-Methyloxane-2,3,4,5-Tetrol | 6-Methyloxane-2,3,4,5-Tetrol | 6-Methyloxane-2,3,4,5-Tetrol | Oxane-2,3,4,5,6-Pentol | Polymerization | 604.2064 | 586.1908 |
|  | 6-Methyloxane-2,3,4,5-Tetrol | 6-Methyloxane-2,3,4,5-Tetrol | 6-Methyloxane-2,3,4,5-Tetrol | 3,4,5,6-Tetrahydroxyoxane-2-Carboxylic Acid | Polymerization | 632.2013 | 614.1857 |
|  | 6-Methyloxane-2,3,4,5-Tetrol | 6-Methyloxane-2,3,4,5-Tetrol | Oxane-2,3,4,5-Tetrol | Oxane-2,3,4,5-Tetrol | Polymerization | 574.1958 | 556.1802 |
|  | 6-Methyloxane-2,3,4,5-Tetrol | 6-Methyloxane-2,3,4,5-Tetrol | Oxane-2,3,4,5-Tetrol | 6-(Hydroxymethyl)Oxane-2,3,4,5-Tetrol | Polymerization | 604.2064 | 586.1908 |
|  | 6-Methyloxane-2,3,4,5-Tetrol | 6-Methyloxane-2,3,4,5-Tetrol | Oxane-2,3,4,5-Tetrol | 5-(Hydroxymethyl)Oxolane-2,3,4-Triol | Polymerization | 574.1958 | 556.1802 |
|  | 6-Methyloxane-2,3,4,5-Tetrol | 6-Methyloxane-2,3,4,5-Tetrol | Oxane-2,3,4,5-Tetrol | Oxane-2,3,4,5,6-Pentol | Polymerization | 590.1907 | 572.1751 |
|  | 6-Methyloxane-2,3,4,5-Tetrol | 6-Methyloxane-2,3,4,5-Tetrol | Oxane-2,3,4,5-Tetrol | 3,4,5,6-Tetrahydroxyoxane-2-Carboxylic Acid | Polymerization | 618.1856 | 600.1700 |
|  | 6-Methyloxane-2,3,4,5-Tetrol | 6-Methyloxane-2,3,4,5-Tetrol | 6-(Hydroxymethyl)Oxane-2,3,4,5-Tetrol | 6-(Hydroxymethyl)Oxane-2,3,4,5-Tetrol | Polymerization | 634.2169 | 616.2013 |
|  | 6-Methyloxane-2,3,4,5-Tetrol | 6-Methyloxane-2,3,4,5-Tetrol | 6-(Hydroxymethyl)Oxane-2,3,4,5-Tetrol | 5-(Hydroxymethyl)Oxolane-2,3,4-Triol | Polymerization | 604.2064 | 586.1908 |
|  | 6-Methyloxane-2,3,4,5-Tetrol | 6-Methyloxane-2,3,4,5-Tetrol | 6-(Hydroxymethyl)Oxane-2,3,4,5-Tetrol | Oxane-2,3,4,5,6-Pentol | Polymerization | 620.2013 | 602.1857 |
|  | 6-Methyloxane-2,3,4,5-Tetrol | 6-Methyloxane-2,3,4,5-Tetrol | 6-(Hydroxymethyl)Oxane-2,3,4,5-Tetrol | 3,4,5,6-Tetrahydroxyoxane-2-Carboxylic Acid | Polymerization | 648.1962 | 630.1806 |
|  | 6-Methyloxane-2,3,4,5-Tetrol | 6-Methyloxane-2,3,4,5-Tetrol | 5-(Hydroxymethyl)Oxolane-2,3,4-Triol | 5-(Hydroxymethyl)Oxolane-2,3,4-Triol | Polymerization | 574.1958 | 556.1802 |
|  | 6-Methyloxane-2,3,4,5-Tetrol | 6-Methyloxane-2,3,4,5-Tetrol | 5-(Hydroxymethyl)Oxolane-2,3,4-Triol | Oxane-2,3,4,5,6-Pentol | Polymerization | 590.1907 | 572.1751 |
|  | 6-Methyloxane-2,3,4,5-Tetrol | 6-Methyloxane-2,3,4,5-Tetrol | 5-(Hydroxymethyl)Oxolane-2,3,4-Triol | 3,4,5,6-Tetrahydroxyoxane-2-Carboxylic Acid | Polymerization | 618.1856 | 600.1700 |
|  | 6-Methyloxane-2,3,4,5-Tetrol | 6-Methyloxane-2,3,4,5-Tetrol | Oxane-2,3,4,5,6-Pentol | Oxane-2,3,4,5,6-Pentol | Polymerization | 606.1856 | 588.1700 |
|  | 6-Methyloxane-2,3,4,5-Tetrol | 6-Methyloxane-2,3,4,5-Tetrol | Oxane-2,3,4,5,6-Pentol | 3,4,5,6-Tetrahydroxyoxane-2-Carboxylic Acid | Polymerization | 634.1805 | 616.1649 |
|  | 6-Methyloxane-2,3,4,5-Tetrol | 6-Methyloxane-2,3,4,5-Tetrol | 3,4,5,6-Tetrahydroxyoxane-2-Carboxylic Acid | 3,4,5,6-Tetrahydroxyoxane-2-Carboxylic Acid | Polymerization | 662.1755 | 644.1599 |
|  | 6-Methyloxane-2,3,4,5-Tetrol | Oxane-2,3,4,5-Tetrol | Oxane-2,3,4,5-Tetrol | Oxane-2,3,4,5-Tetrol | Polymerization | 546.1645 | 528.1489 |
|  | 6-Methyloxane-2,3,4,5-Tetrol | Oxane-2,3,4,5-Tetrol | Oxane-2,3,4,5-Tetrol | 6-(Hydroxymethyl)Oxane-2,3,4,5-Tetrol | Polymerization | 576.1751 | 558.1595 |
|  | 6-Methyloxane-2,3,4,5-Tetrol | Oxane-2,3,4,5-Tetrol | Oxane-2,3,4,5-Tetrol | 5-(Hydroxymethyl)Oxolane-2,3,4-Triol | Polymerization | 546.1645 | 528.1489 |
|  | 6-Methyloxane-2,3,4,5-Tetrol | Oxane-2,3,4,5-Tetrol | Oxane-2,3,4,5-Tetrol | Oxane-2,3,4,5,6-Pentol | Polymerization | 562.1594 | 544.1438 |
|  | 6-Methyloxane-2,3,4,5-Tetrol | Oxane-2,3,4,5-Tetrol | Oxane-2,3,4,5-Tetrol | 3,4,5,6-Tetrahydroxyoxane-2-Carboxylic Acid | Polymerization | 590.1543 | 572.1387 |
|  | 6-Methyloxane-2,3,4,5-Tetrol | Oxane-2,3,4,5-Tetrol | 6-(Hydroxymethyl)Oxane-2,3,4,5-Tetrol | 6-(Hydroxymethyl)Oxane-2,3,4,5-Tetrol | Polymerization | 606.1856 | 588.1700 |
|  | 6-Methyloxane-2,3,4,5-Tetrol | Oxane-2,3,4,5-Tetrol | 6-(Hydroxymethyl)Oxane-2,3,4,5-Tetrol | 5-(Hydroxymethyl)Oxolane-2,3,4-Triol | Polymerization | 576.1751 | 558.1595 |
|  | 6-Methyloxane-2,3,4,5-Tetrol | Oxane-2,3,4,5-Tetrol | 6-(Hydroxymethyl)Oxane-2,3,4,5-Tetrol | Oxane-2,3,4,5,6-Pentol | Polymerization | 592.1700 | 574.1544 |
|  | 6-Methyloxane-2,3,4,5-Tetrol | Oxane-2,3,4,5-Tetrol | 6-(Hydroxymethyl)Oxane-2,3,4,5-Tetrol | 3,4,5,6-Tetrahydroxyoxane-2-Carboxylic Acid | Polymerization | 620.1649 | 602.1493 |
|  | 6-Methyloxane-2,3,4,5-Tetrol | Oxane-2,3,4,5-Tetrol | 5-(Hydroxymethyl)Oxolane-2,3,4-Triol | 5-(Hydroxymethyl)Oxolane-2,3,4-Triol | Polymerization | 546.1645 | 528.1489 |
|  | 6-Methyloxane-2,3,4,5-Tetrol | Oxane-2,3,4,5-Tetrol | 5-(Hydroxymethyl)Oxolane-2,3,4-Triol | Oxane-2,3,4,5,6-Pentol | Polymerization | 562.1594 | 544.1438 |
|  | 6-Methyloxane-2,3,4,5-Tetrol | Oxane-2,3,4,5-Tetrol | 5-(Hydroxymethyl)Oxolane-2,3,4-Triol | 3,4,5,6-Tetrahydroxyoxane-2-Carboxylic Acid | Polymerization | 590.1543 | 572.1387 |
|  | 6-Methyloxane-2,3,4,5-Tetrol | Oxane-2,3,4,5-Tetrol | Oxane-2,3,4,5,6-Pentol | Oxane-2,3,4,5,6-Pentol | Polymerization | 578.1543 | 560.1387 |
|  | 6-Methyloxane-2,3,4,5-Tetrol | Oxane-2,3,4,5-Tetrol | Oxane-2,3,4,5,6-Pentol | 3,4,5,6-Tetrahydroxyoxane-2-Carboxylic Acid | Polymerization | 606.1492 | 588.1336 |
|  | 6-Methyloxane-2,3,4,5-Tetrol | Oxane-2,3,4,5-Tetrol | 3,4,5,6-Tetrahydroxyoxane-2-Carboxylic Acid | 3,4,5,6-Tetrahydroxyoxane-2-Carboxylic Acid | Polymerization | 634.1442 | 616.1286 |
|  | 6-Methyloxane-2,3,4,5-Tetrol | 6-(Hydroxymethyl)Oxane-2,3,4,5-Tetrol | 6-(Hydroxymethyl)Oxane-2,3,4,5-Tetrol | 6-(Hydroxymethyl)Oxane-2,3,4,5-Tetrol | Polymerization | 666.2068 | 648.1912 |
|  | 6-Methyloxane-2,3,4,5-Tetrol | 6-(Hydroxymethyl)Oxane-2,3,4,5-Tetrol | 6-(Hydroxymethyl)Oxane-2,3,4,5-Tetrol | 5-(Hydroxymethyl)Oxolane-2,3,4-Triol | Polymerization | 636.1962 | 618.1806 |
|  | 6-Methyloxane-2,3,4,5-Tetrol | 6-(Hydroxymethyl)Oxane-2,3,4,5-Tetrol | 6-(Hydroxymethyl)Oxane-2,3,4,5-Tetrol | Oxane-2,3,4,5,6-Pentol | Polymerization | 652.1911 | 634.1755 |
|  | 6-Methyloxane-2,3,4,5-Tetrol | 6-(Hydroxymethyl)Oxane-2,3,4,5-Tetrol | 6-(Hydroxymethyl)Oxane-2,3,4,5-Tetrol | 3,4,5,6-Tetrahydroxyoxane-2-Carboxylic Acid | Polymerization | 680.1860 | 662.1704 |
|  | 6-Methyloxane-2,3,4,5-Tetrol | 6-(Hydroxymethyl)Oxane-2,3,4,5-Tetrol | 5-(Hydroxymethyl)Oxolane-2,3,4-Triol | 5-(Hydroxymethyl)Oxolane-2,3,4-Triol | Polymerization | 606.1856 | 588.1700 |
|  | 6-Methyloxane-2,3,4,5-Tetrol | 6-(Hydroxymethyl)Oxane-2,3,4,5-Tetrol | 5-(Hydroxymethyl)Oxolane-2,3,4-Triol | Oxane-2,3,4,5,6-Pentol | Polymerization | 622.1805 | 604.1649 |
|  | 6-Methyloxane-2,3,4,5-Tetrol | 6-(Hydroxymethyl)Oxane-2,3,4,5-Tetrol | 5-(Hydroxymethyl)Oxolane-2,3,4-Triol | 3,4,5,6-Tetrahydroxyoxane-2-Carboxylic Acid | Polymerization | 650.1755 | 632.1599 |
|  | 6-Methyloxane-2,3,4,5-Tetrol | 6-(Hydroxymethyl)Oxane-2,3,4,5-Tetrol | Oxane-2,3,4,5,6-Pentol | Oxane-2,3,4,5,6-Pentol | Polymerization | 638.1755 | 620.1599 |
|  | 6-Methyloxane-2,3,4,5-Tetrol | 6-(Hydroxymethyl)Oxane-2,3,4,5-Tetrol | Oxane-2,3,4,5,6-Pentol | 3,4,5,6-Tetrahydroxyoxane-2-Carboxylic Acid | Polymerization | 666.1704 | 648.1548 |
|  | 6-Methyloxane-2,3,4,5-Tetrol | 6-(Hydroxymethyl)Oxane-2,3,4,5-Tetrol | 3,4,5,6-Tetrahydroxyoxane-2-Carboxylic Acid | 3,4,5,6-Tetrahydroxyoxane-2-Carboxylic Acid | Polymerization | 666.1704 | 648.1548 |
|  | 6-Methyloxane-2,3,4,5-Tetrol | 5-(Hydroxymethyl)Oxolane-2,3,4-Triol | 5-(Hydroxymethyl)Oxolane-2,3,4-Triol | 5-(Hydroxymethyl)Oxolane-2,3,4-Triol | Polymerization | 546.1645 | 528.1489 |
|  | 6-Methyloxane-2,3,4,5-Tetrol | 5-(Hydroxymethyl)Oxolane-2,3,4-Triol | 5-(Hydroxymethyl)Oxolane-2,3,4-Triol | Oxane-2,3,4,5,6-Pentol | Polymerization | 562.1594 | 544.1438 |
|  | 6-Methyloxane-2,3,4,5-Tetrol | 5-(Hydroxymethyl)Oxolane-2,3,4-Triol | 5-(Hydroxymethyl)Oxolane-2,3,4-Triol | 3,4,5,6-Tetrahydroxyoxane-2-Carboxylic Acid | Polymerization | 590.1543 | 572.1387 |
|  | 6-Methyloxane-2,3,4,5-Tetrol | 5-(Hydroxymethyl)Oxolane-2,3,4-Triol | Oxane-2,3,4,5,6-Pentol | Oxane-2,3,4,5,6-Pentol | Polymerization | 578.1543 | 560.1387 |
|  | 6-Methyloxane-2,3,4,5-Tetrol | 5-(Hydroxymethyl)Oxolane-2,3,4-Triol | Oxane-2,3,4,5,6-Pentol | 3,4,5,6-Tetrahydroxyoxane-2-Carboxylic Acid | Polymerization | 606.1492 | 588.1336 |
|  | 6-Methyloxane-2,3,4,5-Tetrol | 5-(Hydroxymethyl)Oxolane-2,3,4-Triol | 3,4,5,6-Tetrahydroxyoxane-2-Carboxylic Acid | 3,4,5,6-Tetrahydroxyoxane-2-Carboxylic Acid | Polymerization | 634.1442 | 616.1286 |
|  | 6-Methyloxane-2,3,4,5-Tetrol | Oxane-2,3,4,5,6-Pentol | Oxane-2,3,4,5,6-Pentol | Oxane-2,3,4,5,6-Pentol | Polymerization | 610.1442 | 592.1286 |
|  | 6-Methyloxane-2,3,4,5-Tetrol | Oxane-2,3,4,5,6-Pentol | Oxane-2,3,4,5,6-Pentol | 3,4,5,6-Tetrahydroxyoxane-2-Carboxylic Acid | Polymerization | 638.1391 | 620.1235 |
|  | 6-Methyloxane-2,3,4,5-Tetrol | Oxane-2,3,4,5,6-Pentol | 3,4,5,6-Tetrahydroxyoxane-2-Carboxylic Acid | 3,4,5,6-Tetrahydroxyoxane-2-Carboxylic Acid | Polymerization | 666.1340 | 648.1184 |
|  | Oxane-2,3,4,5-Tetrol | Oxane-2,3,4,5-Tetrol | Oxane-2,3,4,5-Tetrol | Oxane-2,3,4,5-Tetrol | Polymerization | 546.1645 | 528.1489 |
|  | Oxane-2,3,4,5-Tetrol | Oxane-2,3,4,5-Tetrol | Oxane-2,3,4,5-Tetrol | 6-(Hydroxymethyl)Oxane-2,3,4,5-Tetrol | Polymerization | 576.1751 | 558.1595 |
|  | Oxane-2,3,4,5-Tetrol | Oxane-2,3,4,5-Tetrol | Oxane-2,3,4,5-Tetrol | 5-(Hydroxymethyl)Oxolane-2,3,4-Triol | Polymerization | 546.1645 | 528.1489 |
|  | Oxane-2,3,4,5-Tetrol | Oxane-2,3,4,5-Tetrol | Oxane-2,3,4,5-Tetrol | Oxane-2,3,4,5,6-Pentol | Polymerization | 562.1594 | 544.1438 |
|  | Oxane-2,3,4,5-Tetrol | Oxane-2,3,4,5-Tetrol | Oxane-2,3,4,5-Tetrol | 3,4,5,6-Tetrahydroxyoxane-2-Carboxylic Acid | Polymerization | 590.1543 | 572.1387 |
|  | Oxane-2,3,4,5-Tetrol | Oxane-2,3,4,5-Tetrol | 6-(Hydroxymethyl)Oxane-2,3,4,5-Tetrol | 6-(Hydroxymethyl)Oxane-2,3,4,5-Tetrol | Polymerization | 636.1962 | 618.1806 |
|  | Oxane-2,3,4,5-Tetrol | Oxane-2,3,4,5-Tetrol | 6-(Hydroxymethyl)Oxane-2,3,4,5-Tetrol | 5-(Hydroxymethyl)Oxolane-2,3,4-Triol | Polymerization | 606.1856 | 588.1700 |
|  | Oxane-2,3,4,5-Tetrol | Oxane-2,3,4,5-Tetrol | 6-(Hydroxymethyl)Oxane-2,3,4,5-Tetrol | Oxane-2,3,4,5,6-Pentol | Polymerization | 622.1805 | 604.1649 |
|  | Oxane-2,3,4,5-Tetrol | Oxane-2,3,4,5-Tetrol | 6-(Hydroxymethyl)Oxane-2,3,4,5-Tetrol | 3,4,5,6-Tetrahydroxyoxane-2-Carboxylic Acid | Polymerization | 650.1755 | 632.1599 |
|  | Oxane-2,3,4,5-Tetrol | Oxane-2,3,4,5-Tetrol | 5-(Hydroxymethyl)Oxolane-2,3,4-Triol | 5-(Hydroxymethyl)Oxolane-2,3,4-Triol | Polymerization | 546.1645 | 528.1489 |
|  | Oxane-2,3,4,5-Tetrol | Oxane-2,3,4,5-Tetrol | 5-(Hydroxymethyl)Oxolane-2,3,4-Triol | Oxane-2,3,4,5,6-Pentol | Polymerization | 562.1594 | 544.1438 |
|  | Oxane-2,3,4,5-Tetrol | Oxane-2,3,4,5-Tetrol | 5-(Hydroxymethyl)Oxolane-2,3,4-Triol | 3,4,5,6-Tetrahydroxyoxane-2-Carboxylic Acid | Polymerization | 590.1543 | 572.1387 |
|  | Oxane-2,3,4,5-Tetrol | Oxane-2,3,4,5-Tetrol | Oxane-2,3,4,5,6-Pentol | Oxane-2,3,4,5,6-Pentol | Polymerization | 578.1543 | 560.1387 |
|  | Oxane-2,3,4,5-Tetrol | Oxane-2,3,4,5-Tetrol | Oxane-2,3,4,5,6-Pentol | 3,4,5,6-Tetrahydroxyoxane-2-Carboxylic Acid | Polymerization | 606.1492 | 588.1336 |
|  | Oxane-2,3,4,5-Tetrol | Oxane-2,3,4,5-Tetrol | 3,4,5,6-Tetrahydroxyoxane-2-Carboxylic Acid | 3,4,5,6-Tetrahydroxyoxane-2-Carboxylic Acid | Polymerization | 634.1442 | 616.1286 |
|  | Oxane-2,3,4,5-Tetrol | 6-(Hydroxymethyl)Oxane-2,3,4,5-Tetrol | 6-(Hydroxymethyl)Oxane-2,3,4,5-Tetrol | 6-(Hydroxymethyl)Oxane-2,3,4,5-Tetrol | Polymerization | 636.1962 | 618.1806 |
|  | Oxane-2,3,4,5-Tetrol | 6-(Hydroxymethyl)Oxane-2,3,4,5-Tetrol | 6-(Hydroxymethyl)Oxane-2,3,4,5-Tetrol | 5-(Hydroxymethyl)Oxolane-2,3,4-Triol | Polymerization | 606.1856 | 588.1700 |
|  | Oxane-2,3,4,5-Tetrol | 6-(Hydroxymethyl)Oxane-2,3,4,5-Tetrol | 6-(Hydroxymethyl)Oxane-2,3,4,5-Tetrol | Oxane-2,3,4,5,6-Pentol | Polymerization | 622.1805 | 604.1649 |
|  | Oxane-2,3,4,5-Tetrol | 6-(Hydroxymethyl)Oxane-2,3,4,5-Tetrol | 6-(Hydroxymethyl)Oxane-2,3,4,5-Tetrol | 3,4,5,6-Tetrahydroxyoxane-2-Carboxylic Acid | Polymerization | 650.1755 | 632.1599 |
|  | Oxane-2,3,4,5-Tetrol | 6-(Hydroxymethyl)Oxane-2,3,4,5-Tetrol | 5-(Hydroxymethyl)Oxolane-2,3,4-Triol | 5-(Hydroxymethyl)Oxolane-2,3,4-Triol | Polymerization | 576.1751 | 558.1595 |
|  | Oxane-2,3,4,5-Tetrol | 6-(Hydroxymethyl)Oxane-2,3,4,5-Tetrol | 5-(Hydroxymethyl)Oxolane-2,3,4-Triol | Oxane-2,3,4,5,6-Pentol | Polymerization | 592.1700 | 574.1544 |
|  | Oxane-2,3,4,5-Tetrol | 6-(Hydroxymethyl)Oxane-2,3,4,5-Tetrol | 5-(Hydroxymethyl)Oxolane-2,3,4-Triol | 3,4,5,6-Tetrahydroxyoxane-2-Carboxylic Acid | Polymerization | 620.1649 | 602.1493 |
|  | Oxane-2,3,4,5-Tetrol | 6-(Hydroxymethyl)Oxane-2,3,4,5-Tetrol | Oxane-2,3,4,5,6-Pentol | Oxane-2,3,4,5,6-Pentol | Polymerization | 608.1649 | 590.1493 |
|  | Oxane-2,3,4,5-Tetrol | 6-(Hydroxymethyl)Oxane-2,3,4,5-Tetrol | Oxane-2,3,4,5,6-Pentol | 3,4,5,6-Tetrahydroxyoxane-2-Carboxylic Acid | Polymerization | 636.1598 | 618.1442 |
|  | Oxane-2,3,4,5-Tetrol | 6-(Hydroxymethyl)Oxane-2,3,4,5-Tetrol | 3,4,5,6-Tetrahydroxyoxane-2-Carboxylic Acid | 3,4,5,6-Tetrahydroxyoxane-2-Carboxylic Acid | Polymerization | 664.1547 | 646.1391 |
|  | Oxane-2,3,4,5-Tetrol | 5-(Hydroxymethyl)Oxolane-2,3,4-Triol | 5-(Hydroxymethyl)Oxolane-2,3,4-Triol | 5-(Hydroxymethyl)Oxolane-2,3,4-Triol | Polymerization | 546.1645 | 528.1489 |
|  | Oxane-2,3,4,5-Tetrol | 5-(Hydroxymethyl)Oxolane-2,3,4-Triol | 5-(Hydroxymethyl)Oxolane-2,3,4-Triol | Oxane-2,3,4,5,6-Pentol | Polymerization | 562.1594 | 544.1438 |
|  | Oxane-2,3,4,5-Tetrol | 5-(Hydroxymethyl)Oxolane-2,3,4-Triol | 5-(Hydroxymethyl)Oxolane-2,3,4-Triol | 3,4,5,6-Tetrahydroxyoxane-2-Carboxylic Acid | Polymerization | 590.1543 | 572.1387 |
|  | Oxane-2,3,4,5-Tetrol | 5-(Hydroxymethyl)Oxolane-2,3,4-Triol | Oxane-2,3,4,5,6-Pentol | Oxane-2,3,4,5,6-Pentol | Polymerization | 578.1543 | 560.1387 |
|  | Oxane-2,3,4,5-Tetrol | 5-(Hydroxymethyl)Oxolane-2,3,4-Triol | Oxane-2,3,4,5,6-Pentol | 3,4,5,6-Tetrahydroxyoxane-2-Carboxylic Acid | Polymerization | 606.1492 | 588.1336 |
|  | Oxane-2,3,4,5-Tetrol | 5-(Hydroxymethyl)Oxolane-2,3,4-Triol | 3,4,5,6-Tetrahydroxyoxane-2-Carboxylic Acid | 3,4,5,6-Tetrahydroxyoxane-2-Carboxylic Acid | Polymerization | 634.1442 | 616.1286 |
|  | Oxane-2,3,4,5-Tetrol | Oxane-2,3,4,5,6-Pentol | Oxane-2,3,4,5,6-Pentol | Oxane-2,3,4,5,6-Pentol | Polymerization | 594.1492 | 576.1336 |
|  | Oxane-2,3,4,5-Tetrol | Oxane-2,3,4,5,6-Pentol | Oxane-2,3,4,5,6-Pentol | 3,4,5,6-Tetrahydroxyoxane-2-Carboxylic Acid | Polymerization | 622.1442 | 604.1286 |
|  | Oxane-2,3,4,5-Tetrol | Oxane-2,3,4,5,6-Pentol | 3,4,5,6-Tetrahydroxyoxane-2-Carboxylic Acid | 3,4,5,6-Tetrahydroxyoxane-2-Carboxylic Acid | Polymerization | 650.1391 | 632.1235 |
|  | Oxane-2,3,4,5-Tetrol | 3,4,5,6-Tetrahydroxyoxane-2-Carboxylic Acid | 3,4,5,6-Tetrahydroxyoxane-2-Carboxylic Acid | 3,4,5,6-Tetrahydroxyoxane-2-Carboxylic Acid | Polymerization | 678.1340 | 660.1184 |
|  | 6-(Hydroxymethyl)Oxane-2,3,4,5-Tetrol | 6-(Hydroxymethyl)Oxane-2,3,4,5-Tetrol | 6-(Hydroxymethyl)Oxane-2,3,4,5-Tetrol | 6-(Hydroxymethyl)Oxane-2,3,4,5-Tetrol | Polymerization | 666.2068 | 648.1912 |
|  | 6-(Hydroxymethyl)Oxane-2,3,4,5-Tetrol | 6-(Hydroxymethyl)Oxane-2,3,4,5-Tetrol | 6-(Hydroxymethyl)Oxane-2,3,4,5-Tetrol | 5-(Hydroxymethyl)Oxolane-2,3,4-Triol | Polymerization | 636.1962 | 618.1806 |
|  | 6-(Hydroxymethyl)Oxane-2,3,4,5-Tetrol | 6-(Hydroxymethyl)Oxane-2,3,4,5-Tetrol | 6-(Hydroxymethyl)Oxane-2,3,4,5-Tetrol | Oxane-2,3,4,5,6-Pentol | Polymerization | 652.1911 | 634.1755 |
|  | 6-(Hydroxymethyl)Oxane-2,3,4,5-Tetrol | 6-(Hydroxymethyl)Oxane-2,3,4,5-Tetrol | 6-(Hydroxymethyl)Oxane-2,3,4,5-Tetrol | 3,4,5,6-Tetrahydroxyoxane-2-Carboxylic Acid | Polymerization | 680.1860 | 662.1704 |
|  | 6-(Hydroxymethyl)Oxane-2,3,4,5-Tetrol | 6-(Hydroxymethyl)Oxane-2,3,4,5-Tetrol | 5-(Hydroxymethyl)Oxolane-2,3,4-Triol | 5-(Hydroxymethyl)Oxolane-2,3,4-Triol | Polymerization | 606.1856 | 588.1700 |
|  | 6-(Hydroxymethyl)Oxane-2,3,4,5-Tetrol | 6-(Hydroxymethyl)Oxane-2,3,4,5-Tetrol | 5-(Hydroxymethyl)Oxolane-2,3,4-Triol | Oxane-2,3,4,5,6-Pentol | Polymerization | 622.1805 | 604.1649 |
|  | 6-(Hydroxymethyl)Oxane-2,3,4,5-Tetrol | 6-(Hydroxymethyl)Oxane-2,3,4,5-Tetrol | 5-(Hydroxymethyl)Oxolane-2,3,4-Triol | 3,4,5,6-Tetrahydroxyoxane-2-Carboxylic Acid | Polymerization | 650.1755 | 632.1599 |
|  | 6-(Hydroxymethyl)Oxane-2,3,4,5-Tetrol | 6-(Hydroxymethyl)Oxane-2,3,4,5-Tetrol | Oxane-2,3,4,5,6-Pentol | Oxane-2,3,4,5,6-Pentol | Polymerization | 638.1755 | 620.1599 |
|  | 6-(Hydroxymethyl)Oxane-2,3,4,5-Tetrol | 6-(Hydroxymethyl)Oxane-2,3,4,5-Tetrol | Oxane-2,3,4,5,6-Pentol | 3,4,5,6-Tetrahydroxyoxane-2-Carboxylic Acid | Polymerization | 666.1704 | 648.1548 |
|  | 6-(Hydroxymethyl)Oxane-2,3,4,5-Tetrol | 6-(Hydroxymethyl)Oxane-2,3,4,5-Tetrol | 3,4,5,6-Tetrahydroxyoxane-2-Carboxylic Acid | 3,4,5,6-Tetrahydroxyoxane-2-Carboxylic Acid | Polymerization | 694.1653 | 676.1497 |
|  | 6-(Hydroxymethyl)Oxane-2,3,4,5-Tetrol | 5-(Hydroxymethyl)Oxolane-2,3,4-Triol | 5-(Hydroxymethyl)Oxolane-2,3,4-Triol | 5-(Hydroxymethyl)Oxolane-2,3,4-Triol | Polymerization | 576.1751 | 558.1595 |
|  | 6-(Hydroxymethyl)Oxane-2,3,4,5-Tetrol | 5-(Hydroxymethyl)Oxolane-2,3,4-Triol | 5-(Hydroxymethyl)Oxolane-2,3,4-Triol | Oxane-2,3,4,5,6-Pentol | Polymerization | 592.1700 | 574.1544 |
|  | 6-(Hydroxymethyl)Oxane-2,3,4,5-Tetrol | 5-(Hydroxymethyl)Oxolane-2,3,4-Triol | 5-(Hydroxymethyl)Oxolane-2,3,4-Triol | 3,4,5,6-Tetrahydroxyoxane-2-Carboxylic Acid | Polymerization | 620.1649 | 602.1493 |
|  | 6-(Hydroxymethyl)Oxane-2,3,4,5-Tetrol | 5-(Hydroxymethyl)Oxolane-2,3,4-Triol | Oxane-2,3,4,5,6-Pentol | Oxane-2,3,4,5,6-Pentol | Polymerization | 608.1649 | 590.1493 |
|  | 6-(Hydroxymethyl)Oxane-2,3,4,5-Tetrol | 5-(Hydroxymethyl)Oxolane-2,3,4-Triol | Oxane-2,3,4,5,6-Pentol | 3,4,5,6-Tetrahydroxyoxane-2-Carboxylic Acid | Polymerization | 636.1598 | 618.1442 |
|  | 6-(Hydroxymethyl)Oxane-2,3,4,5-Tetrol | 5-(Hydroxymethyl)Oxolane-2,3,4-Triol | 3,4,5,6-Tetrahydroxyoxane-2-Carboxylic Acid | 3,4,5,6-Tetrahydroxyoxane-2-Carboxylic Acid | Polymerization | 664.1547 | 646.1391 |
|  | 6-(Hydroxymethyl)Oxane-2,3,4,5-Tetrol | Oxane-2,3,4,5,6-Pentol | Oxane-2,3,4,5,6-Pentol | Oxane-2,3,4,5,6-Pentol | Polymerization | 624.1598 | 606.1442 |
|  | 6-(Hydroxymethyl)Oxane-2,3,4,5-Tetrol | Oxane-2,3,4,5,6-Pentol | Oxane-2,3,4,5,6-Pentol | 3,4,5,6-Tetrahydroxyoxane-2-Carboxylic Acid | Polymerization | 652.1547 | 634.1391 |
|  | 6-(Hydroxymethyl)Oxane-2,3,4,5-Tetrol | Oxane-2,3,4,5,6-Pentol | 3,4,5,6-Tetrahydroxyoxane-2-Carboxylic Acid | 3,4,5,6-Tetrahydroxyoxane-2-Carboxylic Acid | Polymerization | 680.1496 | 662.1340 |
|  | 6-(Hydroxymethyl)Oxane-2,3,4,5-Tetrol | 3,4,5,6-Tetrahydroxyoxane-2-Carboxylic Acid | 3,4,5,6-Tetrahydroxyoxane-2-Carboxylic Acid | 3,4,5,6-Tetrahydroxyoxane-2-Carboxylic Acid | Polymerization | 708.1445 | 690.1289 |
|  | 5-(Hydroxymethyl)Oxolane-2,3,4-Triol | 5-(Hydroxymethyl)Oxolane-2,3,4-Triol | 5-(Hydroxymethyl)Oxolane-2,3,4-Triol | 5-(Hydroxymethyl)Oxolane-2,3,4-Triol | Polymerization | 546.1645 | 528.1489 |
|  | 5-(Hydroxymethyl)Oxolane-2,3,4-Triol | 5-(Hydroxymethyl)Oxolane-2,3,4-Triol | 5-(Hydroxymethyl)Oxolane-2,3,4-Triol | Oxane-2,3,4,5,6-Pentol | Polymerization | 562.1594 | 544.1438 |
|  | 5-(Hydroxymethyl)Oxolane-2,3,4-Triol | 5-(Hydroxymethyl)Oxolane-2,3,4-Triol | 5-(Hydroxymethyl)Oxolane-2,3,4-Triol | 3,4,5,6-Tetrahydroxyoxane-2-Carboxylic Acid | Polymerization | 590.1543 | 572.1387 |
|  | 5-(Hydroxymethyl)Oxolane-2,3,4-Triol | 5-(Hydroxymethyl)Oxolane-2,3,4-Triol | Oxane-2,3,4,5,6-Pentol | Oxane-2,3,4,5,6-Pentol | Polymerization | 578.1543 | 560.1387 |
|  | 5-(Hydroxymethyl)Oxolane-2,3,4-Triol | 5-(Hydroxymethyl)Oxolane-2,3,4-Triol | Oxane-2,3,4,5,6-Pentol | 3,4,5,6-Tetrahydroxyoxane-2-Carboxylic Acid | Polymerization | 606.1492 | 588.1336 |
|  | 5-(Hydroxymethyl)Oxolane-2,3,4-Triol | 5-(Hydroxymethyl)Oxolane-2,3,4-Triol | 3,4,5,6-Tetrahydroxyoxane-2-Carboxylic Acid | 3,4,5,6-Tetrahydroxyoxane-2-Carboxylic Acid | Polymerization | 634.1442 | 616.1286 |
|  | 5-(Hydroxymethyl)Oxolane-2,3,4-Triol | Oxane-2,3,4,5,6-Pentol | 5-(Hydroxymethyl)Oxolane-2,3,4-Triol | Oxane-2,3,4,5,6-Pentol | Polymerization | 594.1492 | 576.1336 |
|  | 5-(Hydroxymethyl)Oxolane-2,3,4-Triol | Oxane-2,3,4,5,6-Pentol | 5-(Hydroxymethyl)Oxolane-2,3,4-Triol | 3,4,5,6-Tetrahydroxyoxane-2-Carboxylic Acid | Polymerization | 622.1442 | 604.1286 |
|  | 5-(Hydroxymethyl)Oxolane-2,3,4-Triol | Oxane-2,3,4,5,6-Pentol | 3,4,5,6-Tetrahydroxyoxane-2-Carboxylic Acid | 3,4,5,6-Tetrahydroxyoxane-2-Carboxylic Acid | Polymerization | 650.1391 | 632.1235 |
|  | 5-(Hydroxymethyl)Oxolane-2,3,4-Triol | 3,4,5,6-Tetrahydroxyoxane-2-Carboxylic Acid | 3,4,5,6-Tetrahydroxyoxane-2-Carboxylic Acid | 3,4,5,6-Tetrahydroxyoxane-2-Carboxylic Acid | Polymerization | 678.1340 | 660.1184 |
|  | Oxane-2,3,4,5,6-Pentol | Oxane-2,3,4,5,6-Pentol | 5-(Hydroxymethyl)Oxolane-2,3,4-Triol | Oxane-2,3,4,5,6-Pentol | Polymerization | 610.1442 | 592.1286 |
|  | Oxane-2,3,4,5,6-Pentol | Oxane-2,3,4,5,6-Pentol | 5-(Hydroxymethyl)Oxolane-2,3,4-Triol | 3,4,5,6-Tetrahydroxyoxane-2-Carboxylic Acid | Polymerization | 638.1391 | 620.1235 |
|  | Oxane-2,3,4,5,6-Pentol | Oxane-2,3,4,5,6-Pentol | 3,4,5,6-Tetrahydroxyoxane-2-Carboxylic Acid | 3,4,5,6-Tetrahydroxyoxane-2-Carboxylic Acid | Polymerization | 666.1340 | 648.1184 |
|  | Oxane-2,3,4,5,6-Pentol | 3,4,5,6-Tetrahydroxyoxane-2-Carboxylic Acid | 5-(Hydroxymethyl)Oxolane-2,3,4-Triol | Oxane-2,3,4,5,6-Pentol | Polymerization | 638.1391 | 620.1235 |
|  | Oxane-2,3,4,5,6-Pentol | 3,4,5,6-Tetrahydroxyoxane-2-Carboxylic Acid | 5-(Hydroxymethyl)Oxolane-2,3,4-Triol | 3,4,5,6-Tetrahydroxyoxane-2-Carboxylic Acid | Polymerization | 666.1340 | 648.1184 |
|  | Oxane-2,3,4,5,6-Pentol | 3,4,5,6-Tetrahydroxyoxane-2-Carboxylic Acid | 3,4,5,6-Tetrahydroxyoxane-2-Carboxylic Acid | 3,4,5,6-Tetrahydroxyoxane-2-Carboxylic Acid | Polymerization | 694.1289 | 676.1133 |
|  | 3,4,5,6-Tetrahydroxyoxane-2-Carboxylic Acid | 3,4,5,6-Tetrahydroxyoxane-2-Carboxylic Acid | 3,4,5,6-Tetrahydroxyoxane-2-Carboxylic Acid | 3,4,5,6-Tetrahydroxyoxane-2-Carboxylic Acid | Polymerization | 723.1238 | 705.1082 |

Table S5 Identification of glycosides in QC of FG and FGG

| No | Database | t_R_/min | m/z | Adduct type | Formula | MW* | Name | Class | Degree | Total score | Reported in the literature |
| --- | --- | --- | --- | --- | --- | --- | --- | --- | --- | --- | --- |
| 1 | Massbank | 3.3 | 467.0927 | [M-H]- | C20H20O13 | 468.0904 | [3,4-Dihydroxy-5-(3,4,5-Trihydroxybenzoyl)Oxyoxan-2-Yl]Methyl 3,4,5-Trihydroxybenzoate | Benzene glycosides | Level 2 | 0.64 | No |
| 2 | MONA | 3.0 | 383.1363 | [M+Na]+ | C16H24O9 | 360.1420 | 2-[3-Hydroxy-2-(4-Hydroxy-3-Methoxyphenyl)Propoxy]-6-(Hydroxymethyl)Oxane-3,4,5-Triol | Benzene glycosides | Level 2 | 0.80 | Yes |
| 3 | Authentic standards of MS-DIAL | 4.2 | 371.0985 | [M-H]- | C16H20O10 | 372.1056 | Deacetylasperuloside | Benzene glycosides | Level 2 | 0.87 | No |
| 4 | Authentic standards of MS-DIAL | 6.6 | 434.2003 | [M+NH4]+ | C19H28O10 | 416.1682 | 2-[4,5-Dihydroxy-6-(Hydroxymethyl)-2-(2-Phenylethoxy)Oxan-3-Yl]Oxyoxane-3,4,5-Triol | Benzene glycosides | Level 2 | 0.80 | No |
| 5 | Authentic standards of MS-DIAL | 5.7 | 475.1788 | [M+CH3COO]- | C19H28O10 | 416.1682 | 2-[[3,4-Dihydroxy-4-(Hydroxymethyl)Oxolan-2-Yl]Oxymethyl]-6-(2-Phenylethoxy)Oxane-3,4,5-Triol | Benzene glycosides | Level 2 | 0.81 | No |
| 6 | Vaniya- Fiehn_Natural_  Products_Library | 4.2 | 431.1543 | [M-H]- | C19H28O11 | 432.1632 | 2-[[3,4-Dihydroxy-4-(Hydroxymethyl)Oxolan-2-Yl]Oxymethyl]-6-[2-(4-Hydroxyphenyl)Ethoxy]Oxane-3,4,5-Triol | Benzene glycosides | Level 2 | 0.82 | Yes |
| 7 | Authentic standards of MS-DIAL | 5.4 | 473.1623 | [M-H]- | C21H30O12 | 474.1737 | 2-(Hydroxymethyl)-6-[[3,4,5-Trihydroxy-6-(2-Hydroxy-4-Prop-2-Enylphenoxy)Oxan-2-Yl]Methoxy]Oxane-3,4,5-Triol | Benzene glycosides | Level 2 | 0.70 | No |
| 8 | COCONUT | 6.4 | 477.1935 | [M+H]+ | C21H32O12 | 476.1894 | 2-[[3,4-Dihydroxy-4-(Hydroxymethyl)Oxolan-2-Yl]Oxymethyl]-6-[(3,4,5-Trimethoxyphenyl)Methyl]Oxane-3,4,5-Triol | Benzene glycosides | Level 3 | 0.59 | Yes |
| 9 | MONA | 3.8 | 299.1116 | [M+H]+ | C14H18O7 | 298.1053 | 1-[4-[3,4,5-Trihydroxy-6-(Hydroxymethyl)Oxan-2-Yl]Oxyphenyl]Ethanone | Benzene glycosides | Level 2 | 0.60 | No |
| 10 | MONA | 3.5 | 351.1063 | [M+Na]+ | C15H20O8 | 328.1158 | 1-[2-Hydroxy-6-Methyl-4-[3,4,5-Trihydroxy-6-(Hydroxymethyl)Oxan-2-Yl]Oxyphenyl]Ethanone | Benzene glycosides | Level 2 | 0.76 | Yes |
| 11 | Authentic standards of MS-DIAL | 5.2 | 327.1441 | [M-H]- | C16H24O7 | 328.1522 | Jasminoside J | Benzene glycosides | Level 2 | 0.86 | Yes |
| 12 | MONA | 9.7 | 351.1401 | [M+Na]+ | C16H24O7 | 328.1522 | Jasminoside C | Benzene glycosides | Level 2 | 0.78 | No |
| 13 | Authentic standards of MS-DIAL | 4.1 | 401.1432 | [M+CH3COO]- | C16H22O8 | 342.1315 | 2-(Hydroxymethyl)-6-[4-(3-Hydroxyprop-1-Enyl)-2-Methoxyphenoxy]Oxane-3,4,5-Triol | Benzene glycosides | Level 2 | 0.75 | Yes |
| 14 | MONA | 7.9 | 417.1171 | [M+Na]+ | C19H22O9 | 394.1264 | 7-Hydroxy-5-Methyl-2-(2-Oxopropyl)-8-[3,4,5-Trihydroxy-6-(Hydroxymethyl)Oxan-2-Yl]Chromen-4-One | Benzene glycosides | Level 2 | 0.63 | No |
| 15 | MONA | 12.0 | 429.1167 | [M+Na]+ | C20H22O9 | 406.1264 | [3,4,5-Trihydroxy-6-[4-Hydroxy-2-(Hydroxymethyl)Phenoxy]Oxan-2-Yl]Methyl Benzoate | Benzene glycosides | Level 2 | 0.70 | No |
| 16 | Authentic standards of MS-DIAL | 4.9 | 175.0382 | [M+Na]+ | C19H26O12 | 446.1424 | Methyl 2-[3,4,5-Trihydroxy-6-[(3,4,5-Trihydroxyoxan-2-Yl)Oxymethyl]Oxan-2-Yl]Oxybenzoate | Benzene glycosides | Level 2 | 0.84 | No |
| 17 | COCONUT | 1.2 | 447.1114 | [M-H]- | C18H24O13 | 448.1217 | 5-[3-[3,4-Dihydroxy-4-(Hydroxymethyl)Oxolan-2-Yl]Oxy-4,5-Dihydroxy-6-(Hydroxymethyl)Oxan-2-Yl]Oxy-2-Hydroxybenzoic Acid | Benzene glycosides | Level 3 | 0.65 | No |
| 18 | COCONUT | 5.6 | 473.1644 | [M-H]- | C21H30O12 | 474.1737 | 2-[2-Hydroxy-3-Methoxy-5-(Prop-2-En-1-Yl)Phenoxy]-6-{[(3,4,5-Trihydroxyoxan-2-Yl)Oxy]Methyl}Oxane-3,4,5-Triol | Benzene glycosides | Level 3 | 0.57 | No |
| 19 | Vaniya- Fiehn_Natural_Products_Library | 4.6 | 477.1614 | [M-H]- | C20H30O13 | 478.1686 | 2-[[3,4-Dihydroxy-4-(Hydroxymethyl)Oxolan-2-Yl]Oxymethyl]-6-(3,4,5-Trimethoxyphenoxy)Oxane-3,4,5-Triol | Benzene glycosides | Level 2 | 0.74 | No |
| 20 | Authentic standards of MS-DIAL | 5.1 | 496.2030 | [M+NH4]+ | C20H30O13 | 478.1686 | 2-[(3,4,5-Trihydroxyoxan-2-Yl)Oxymethyl]-6-(3,4,5-Trimethoxyphenoxy)Oxane-3,4,5-Triol | Benzene glycosides | Level 2 | 0.81 | No |
| 21 | COCONUT | 4.4 | 533.1860 | [M-H]- | C23H34O14 | 534.1949 | 2-(Hydroxymethyl)-6-[[3,4,5-Trihydroxy-6-[4-(3-Hydroxyprop-1-Enyl)-2,6-Dimethoxyphenoxy]Oxan-2-Yl]Methoxy]Oxane-3,4,5-Triol | Benzene glycosides | Level 3 | 0.53 | No |
| 22 | Authentic standards of MS-DIAL | 3.0 | 327.1072 | [M-H]- | C15H20O8 | 328.1158 | 1-(4-Hydroxyphenyl)-3-[3,4,5-Trihydroxy-6-(Hydroxymethyl)Oxan-2-Yl]Oxypropan-1-One | Benzene glycosides | Level 2 | 0.78 | No |
| 23 | Authentic standards of MS-DIAL | 7.3 | 459.1863 | [M-H]- | C21H32O11 | 460.1945 | 2-[[3,4-Dihydroxy-5-(Hydroxymethyl)Oxolan-2-Yl]Oxymethyl]-6-[4-(4-Hydroxyphenyl)Butan-2-Yloxy]Oxane-3,4,5-Triol | Benzene glycosides | Level 2 | 0.74 | No |
| 24 | MONA | 8.8 | 483.1866 | [M+Na]+ | C21H32O11 | 460.1945 | 2-[[3,4-Dihydroxy-4-(Hydroxymethyl)Oxolan-2-Yl]Oxymethyl]-6-[4-(4-Hydroxyphenyl)Butan-2-Yloxy]Oxane-3,4,5-Triol | Benzene glycosides | Level 2 | 0.69 | No |
| 25 | MONA | 10.7 | 485.2049 | [M+Na]+ | C25H34O8 | 462.2254 | 2-[1,7-Bis(4-Hydroxyphenyl)Heptan-3-Yloxy]-6-(Hydroxymethyl)Oxane-3,4,5-Triol | Benzene glycosides | Level 2 | 0.69 | No |
| 26 | MONA | 11.9 | 617.2578 | [M+Na]+ | C30H42O12 | 594.2676 | 6-[1,7-Bis(4-Hydroxyphenyl)Heptan-3-Yloxy]-5-[3,4-Dihydroxy-4-(Hydroxymethyl)Oxolan-2-Yl]Oxy-2-(Hydroxymethyl)Oxane-3,4-Diol | Benzene glycosides | Level 2 | 0.62 | No |
| 27 | MONA | 3.3 | 413.1223 | [M+Na]+ | C20H22O8 | 390.1315 | 2-[3-Hydroxy-5-[2-(4-Hydroxyphenyl)Ethenyl]Phenoxy]-6-(Hydroxymethyl)Oxane-3,4,5-Triol | Benzene glycosides | Level 2 | 0.65 | No |
| 28 | COCONUT | 4.2 | 741.2227 | [M-H]- | C33H42O19 | 742.2320 | [4-[3,4-Dihydroxy-4-(Hydroxymethyl)Oxolan-2-Yl]Oxy-2-[[3,4-Dihydroxy-4-(Hydroxymethyl)Oxolan-2-Yl]Oxymethyl]-6-[2-(3,4-Dihydroxyphenyl)Ethoxy]-5-Hydroxyoxan-3-Yl] 3-(3,4-Dihydroxyphenyl)Prop-2-Enoate | Cinnamic acids glycosides | Level 3 | 0.55 | No |
| 29 | Authentic standards of MS-DIAL | 9.1 | 887.2930 | [M-H]- | C39H52O23 | 888.2899 | [2-[[3,4-Dihydroxy-4-(Hydroxymethyl)Oxolan-2-Yl]Oxymethyl]-4-[4,5-Dihydroxy-6-Methyl-3-(3,4,5-Trihydroxyoxan-2-Yl)Oxyoxan-2-Yl]Oxy-6-[2-(3,4-Dihydroxyphenyl)Ethoxy]-5-Hydroxyoxan-3-Yl] 3-(3,4-Dihydroxyphenyl)Prop-2-Enoate | Cinnamic acids glycosides | Level 2 | 0.74 | No |
| 30 | Authentic standards of MS-DIAL | 10.5 | 293.1016 | [M-H2O+H]+ | C15H18O7 | 310.1053 | [3,4,5-Trihydroxy-6-(Hydroxymethyl)Oxan-2-Yl] 3-Phenylprop-2-Enoate | Cinnamic acids glycosides | Level 2 | 0.79 | No |
| 31 | GNPS | 5.3 | 551.2128 | [M-H]- | C27H36O12 | 552.2207 | 2,6,6-Trimethyl-4-[3,4,5-Trihydroxy-6-[3-(4-Hydroxy-3,5-Dimethoxyphenyl)Prop-2-Enoyloxymethyl]Oxan-2-Yl]Oxycyclohexene-1-Carboxylic Acid | Cinnamic acids glycosides | Level 2 | 0.85 | No |
| 32 | Authentic standards of MS-DIAL | 4.9 | 517.1552 | [M-H]- | C22H30O14 | 518.1636 | [6-[3,4-Dihydroxy-2,5-Bis(Hydroxymethyl)Oxolan-2-Yl]Oxy-3,4,5-Trihydroxyoxan-2-Yl]Methyl 3-(4-Hydroxy-3-Methoxyphenyl)Prop-2-Enoate | Cinnamic acids glycosides | Level 2 | 0.71 | No |
| 33 | MONA | 7.4 | 561.1580 | [M+Na]+ | C25H30O13 | 538.1686 | [2-(Hydroxymethyl)-10-[3,4,5-Trihydroxy-6-(Hydroxymethyl)Oxan-2-Yl]Oxy-3,9-Dioxatricyclo[4.4.0.02,4]Dec-7-En-5-Yl] 3-(4-Hydroxy-3-Methoxyphenyl)Prop-2-Enoate | Cinnamic acids glycosides | Level 2 | 0.63 | No |
| 34 | Authentic standards of MS-DIAL | 5.4 | 566.2047 | [M+NH4]+ | C23H32O15 | 548.1741 | [6-[3,4-Dihydroxy-2,5-Bis(Hydroxymethyl)Oxolan-2-Yl]Oxy-3,4,5-Trihydroxyoxan-2-Yl]Methyl 3-(4-Hydroxy-3,5-Dimethoxyphenyl)Prop-2-Enoate | Cinnamic acids glycosides | Level 2 | 0.81 | Yes |
| 35 | Authentic standards of MS-DIAL | 7.5 | 581.1856 | [M+H]+ | C27H32O14 | 580.1792 | 6-Hydroxy-7-Methylidene-1-[3,4,5-Trihydroxy-6-[3-(4-Hydroxy-3,5-Dimethoxyphenyl)Prop-2-Enoyloxymethyl]Oxan-2-Yl]Oxy-4A,5,6,7A-Tetrahydro-1H-Cyclopenta[C]Pyran-4-Carboxylic Acid | Cinnamic acids glycosides | Level 2 | 0.87 | No |
| 36 | MONA | 8.4 | 721.2316 | [M+Na]+ | C32H42O17 | 698.2422 | [3,5-Dihydroxy-2-[[2-(Hydroxymethyl)-10-[3,4,5-Trihydroxy-6-(Hydroxymethyl)Oxan-2-Yl]Oxy-3,9-Dioxatricyclo[4.4.0.02,4]Dec-7-En-5-Yl]Oxy]-6-Methyloxan-4-Yl] 3-(3,4-Dimethoxyphenyl)Prop-2-Enoate | Cinnamic acids glycosides | Level 2 | 0.62 | No |
| 37 | Authentic standards of MS-DIAL | 6.2 | 309.0961 | [M-H2O+H]+ | C15H18O8 | 326.1002 | [3,4,5-Trihydroxy-6-(Hydroxymethyl)Oxan-2-Yl] 3-(2-Hydroxyphenyl)Prop-2-Enoate | Cinnamic acids glycosides | Level 2 | 0.79 | Yes |
| 38 | MassBank | 4.7 | 385.1132 | [M-H]- | C17H22O10 | 386.1213 | 1-O-Sinapoyl-Beta-D-Glucose | Cinnamic acids glycosides | Level 2 | 0.94 | No |
| 39 | Authentic standards of MS-DIAL | 7.7 | 471.1290 | [M-H]- | C24H24O10 | 472.1369 | [3,4,5-Trihydroxy-6-[3-(4-Hydroxyphenyl)Prop-2-Enoyloxy]Oxan-2-Yl]Methyl 3-(4-Hydroxyphenyl)Prop-2-Enoate | Cinnamic acids glycosides | Level 2 | 0.88 | Yes |
| 40 | Authentic standards of MS-DIAL | 8.4 | 489.1592 | [M+H]+ | C21H28O13 | 488.1530 | [3,4,6-Trihydroxy-5-[3,4,5-Trihydroxy-6-(Hydroxymethyl)Oxan-2-Yl]Oxyoxan-2-Yl]Methyl 3-(4-Hydroxyphenyl)Prop-2-Enoate | Cinnamic acids glycosides | Level 2 | 0.83 | No |
| 41 | Authentic standards of MS-DIAL | 7.2 | 591.1654 | [M-H]- | C28H32O14 | 592.1792 | [4,5-Dihydroxy-2-[3-(4-Hydroxy-3,5-Dimethoxyphenyl)Prop-2-Enoyloxy]-6-(Hydroxymethyl)Oxan-3-Yl] 3-(4-Hydroxy-3,5-Dimethoxyphenyl)Prop-2-Enoate | Cinnamic acids glycosides | Level 2 | 0.72 | Yes |
| 42 | Authentic standards of MS-DIAL | 4.4 | 659.1597 | [M-H]- | C31H32O16 | 660.1690 | Methyl 3,5-Di-O-Caffeoyl-4-O-(3-Hydroxy-3-Methyl)Glutaroylquinate | Cinnamic acids glycosides | Level 2 | 0.85 | No |
| 43 | Authentic standards of MS-DIAL | 4.4 | 325.0898 | [M-H]- | C15H18O8 | 326.1002 | 3-[4-[3,4,5-Trihydroxy-6-(Hydroxymethyl)Oxan-2-Yl]Oxyphenyl]Prop-2-Enoic Acid | Cinnamic acids glycosides | Level 2 | 0.81 | No |
| 44 | MONA | 2.9 | 357.1173 | [M+H]+ | C16H20O9 | 356.1107 | 3-[4-Methoxy-2-[3,4,5-Trihydroxy-6-(Hydroxymethyl)Oxan-2-Yl]Oxyphenyl]Prop-2-Enoic Acid | Cinnamic acids glycosides | Level 2 | 0.63 | No |
| 45 | Authentic standards of MS-DIAL | 2.9 | 337.0885 | [M-H]- | C16H18O8 | 338.1002 | 1,3,5-Trihydroxy-4-[3-(4-Hydroxyphenyl)Prop-2-Enoyloxy]Cyclohexane-1-Carboxylic Acid | Cinnamic acids glycosides | Level 2 | 0.78 | No |
| 46 | Authentic standards of MS-DIAL | 3.7 | 337.0924 | [M-H]- | C16H18O8 | 338.1002 | 3-O-P-Coumaroylquinic Acid | Cinnamic acids glycosides | Level 2 | 0.70 | Yes |
| 47 | Vaniya- Fiehn_Natural_Products_Library | 1.0 | 353.0881 | [M-H]- | C16H18O9 | 354.0951 | 4-O-Trans-Caffeoylquinic Acid | Cinnamic acids glycosides | Level 2 | 0.90 | Yes |
| 48 | Authentic standards of MS-DIAL | 4.5 | 355.1015 | [M+H]+ | C16H18O9 | 354.0951 | Chlorogenic Acid | Cinnamic acids glycosides | Level 2 | 0.86 | No |
| 49 | Vaniya- Fiehn_Natural_Products_Library | 5.9 | 367.1014 | [M-H]- | C17H20O9 | 368.1107 | Methyl 3-[3-(3,4-Dihydroxyphenyl)Prop-2-Enoyloxy]-1,4,5-Trihydroxycyclohexane-1-Carboxylate | Cinnamic acids glycosides | Level 2 | 0.92 | No |
| 50 | Massbank | 4.2 | 367.1018 | [M-H]- | C17H20O9 | 368.1107 | 1,3,5-Trihydroxy-4-[3-(3-Hydroxy-4-Methoxyphenyl)Prop-2-Enoyloxy]Cyclohexane-1-Carboxylic Acid | Cinnamic acids glycosides | Level 2 | 0.67 | Yes |
| 51 | Vaniya- Fiehn_Natural_  Products_Library | 5.2 | 515.1180 | [M-H]- | C25H24O12 | 516.1268 | Isochlorogenic Acid B | Cinnamic acids glycosides | Level 2 | 0.95 | Yes |
| 52 | GNPS | 6.6 | 529.1339 | [M-H]- | C26H26O12 | 530.1424 | 3,5-Di-O-Caffeoylquinic Acid Methyl Ester | Cinnamic acids glycosides | Level 2 | 0.88 | No |
| 53 | COCONUT | 7.5 | 545.1640 | [M+H]+ | C27H28O12 | 544.1581 | 1,3-Bis[4-(3,4-Dihydroxyphenyl)-2-Oxobut-3-Enoxy]-4,5-Dihydroxycyclohexane-1-Carboxylic Acid | Cinnamic acids glycosides | Level 3 | 0.54 | Yes |
| 54 | Authentic standards of MS-DIAL | 5.8 | 559.1454 | [M-H]- | C27H28O13 | 560.1530 | 3-O-Caffeoyl-4-O-Sinapoylquinic Acid | Cinnamic acids glycosides | Level 2 | 0.84 | No |
| 55 | Vaniya- Fiehn_Natural_  Products_Library | 4.9 | 355.1014 | [M+H]+ | C16H18O9 | 354.0951 | Cryptochlorogenic Acid | Cinnamic acids glycosides | Level 1 | 0.83 | No |
| 56 | COCONUT | 3.2 | 659.1779 | [M-H]- | C28H36O18 | 660.1902 | [4-[3,5-Dihydroxy-6-(Hydroxymethyl)-4-[3,4,5-Trihydroxy-6-(Hydroxymethyl)Oxan-2-Yl]Oxyoxan-2-Yl]Oxy-3,5-Dimethoxyphenyl]Methyl 3,4,5-Trihydroxybenzoate | Cinnamic acids glycosides | Level 3 | 0.52 | No |
| 57 | COCONUT | 3.4 | 476.1955 | [M+H]+ | C24H29NO9 | 475.1842 | N-Trans-Feruloyl Tyramine-4'-O-Beta-D-Glucopyranoside | Cinnamic acids glycosides | Level 3 | 0.75 | No |
| 58 | COCONUT | 8.4 | 369.1173 | [M+H]+ | C17H20O9 | 368.1107 | 7-Hydroxy-6-[2-[3,4,5-Trihydroxy-6-(Hydroxymethyl)Oxan-2-Yl]Oxyethyl]Chromen-2-One | Coumarins glycosides | Level 3 | 0.50 | No |
| 59 | MONA | 6.2 | 325.0912 | [M+H]+ | C15H16O8 | 324.0845 | 7-[3,4,5-Trihydroxy-6-(Hydroxymethyl)Oxan-2-Yl]Oxychromen-2-One | Coumarins glycosides | Level 2 | 0.61 | No |
| 60 | MONA | 7.1 | 339.1064 | [M+H]+ | C16H18O8 | 338.1002 | 5-Methyl-4-[3,4,5-Trihydroxy-6-(Hydroxymethyl)Oxan-2-Yl]Oxychromen-2-One | Coumarins glycosides | Level 2 | 0.68 | No |
| 61 | GNPS | 4.3 | 369.0814 | [M-H]- | C16H18O10 | 370.0900 | 7-Hydroxy-6-Methoxy-8-[3,4,5-Trihydroxy-6-(Hydroxymethyl)Oxan-2-Yl]Oxychromen-2-One | Coumarins glycosides | Level 2 | 0.92 | No |
| 62 | Vaniya- Fiehn_Natural_  Products_Library | 7.7 | 449.1058 | [M+H]+ | C21H20O11 | 448.1006 | 4-(3,4-Dihydroxyphenyl)-7-Hydroxy-5-[3,4,5-Trihydroxy-6-(Hydroxymethyl)Oxan-2-Yl]Oxychromen-2-One | Coumarins glycosides | Level 2 | 0.88 | No |
| 63 | Authentic standards of MS-DIAL | 4.9 | 487.1435 | [M+H]+ | C21H26O13 | 486.1373 | 7-[6-[[3,4-Dihydroxy-4-(Hydroxymethyl)Oxolan-2-Yl]Oxymethyl]-3,4,5-Trihydroxyoxan-2-Yl]Oxy-6-Methoxychromen-2-One | Coumarins glycosides | Level 2 | 0.81 | No |
| 64 | GNPS | 4.3 | 545.1494 | [M+FA-H]- | C22H28O13 | 500.1530 | 6-Methoxy-7-[3,4,5-Trihydroxy-6-[(3,4,5-Trihydroxy-6-Methyloxan-2-Yl)Oxymethyl]Oxan-2-Yl]Oxychromen-2-One | Coumarins glycosides | Level 2 | 0.76 | No |
| 65 | COCONUT | 6.8 | 339.1061 | [M+H]+ | C16H18O8 | 338.1002 | 5-Methyl-4-[(3,4,5,6-Tetrahydroxyoxan-2-Yl)Methoxy]Chromen-2-One | Coumarins glycosides | Level 3 | 0.72 | No |
| 66 | COCONUT | 3.3 | 339.0715 | [M-H]- | C15H16O9 | 340.0794 | 5-Hydroxy-7-{[3,4,5-Trihydroxy-6-(Hydroxymethyl)Oxan-2-Yl]Oxy}-2H-Chromen-2-One | Coumarins glycosides | Level 3 | 0.71 | No |
| 67 | COCONUT | 12.6 | 707.1947 | [M+H]+ | C36H34O15 | 706.1898 | 11,22,27-Trihydroxy-7,9,24,26-Tetramethyl-20-[3,4,5-Trihydroxy-6-(Hydroxymethyl)Oxan-2-Yl]Oxy-8,16,25,30-Tetraoxaoctacyclo[15.11.1.14,27.02,15.03,12.05,10.021,29.023,28]Triaconta-1,3,5(10),11,14,17(29),19,21,23(28)-Nonaene-13,18-Dione | Flavonoid glycosides | Level 3 | 0.60 | No |
| 68 | COCONUT | 11.1 | 825.2620 | [M+H]+ | C41H44O18 | 824.2528 | 2-[3-Hydroxy-5-[2-[3-(4-Hydroxy-3-Methoxyphenyl)-2-[3-Hydroxy-5-[3,4,5-Trihydroxy-6-(Hydroxymethyl)Oxan-2-Yl]Oxyphenyl]-2,3-Dihydro-1,4-Benzodioxin-6-Yl]Ethenyl]Phenoxy]-6-(Hydroxymethyl)Oxane-3,4,5-Triol | Flavonoid glycosides | Level 3 | 0.56 | No |
| 69 | Authentic standards of MS-DIAL | 1.2 | 431.0952 | [M+H]+ | C21H18O10 | 430.0900 | 3,4,5-Trihydroxy-6-(5-Hydroxy-4-Oxo-2-Phenylchromen-7-Yl)Oxyoxane-2-Carboxylic Acid | Flavonoid glycosides | Level 2 | 0.80 | Yes |
| 70 | Authentic standards of MS-DIAL | 6.5 | 447.0889 | [M-H]- | C21H20O11 | 448.1006 | Astragalin | Flavonoid glycosides | Level 1 | 0.83 | No |
| 71 | Authentic standards of MS-DIAL | 6.7 | 449.1060 | [M+H]+ | C21H20O11 | 448.1006 | 2-(3,4-Dihydroxyphenyl)-5,7-Dihydroxy-3-(3,4,5-Trihydroxy-6-Methyloxan-2-Yl)Oxychromen-4-One | Flavonoid glycosides | Level 2 | 0.83 | No |
| 72 | MONA | 7.1 | 449.1072 | [M+H]+ | C21H20O11 | 448.1006 | Luteoloside | Flavonoid glycosides | Level 1 | 0.81 | Yes |
| 73 | Vaniya- Fiehn_Natural_Products_Library | 7.4 | 449.1079 | [M+H]+ | C21H20O11 | 448.1006 | Luteolin 7-O-Glucoside | Flavonoid glycosides | Level 2 | 0.87 | No |
| 74 | COCONUT | 6.5 | 449.1074 | [M+H]+ | C21H20O11 | 448.1006 | Isoorientin | Flavonoid glycosides | Level 1 | 0.60 | Yes |
| 75 | Vaniya- Fiehn_Natural_Products_Library | 6.5 | 465.1012 | [M+H]+ | C21H20O12 | 464.0955 | Hyperoside | Flavonoid glycosides | Level 1 | 0.92 | No |
| 76 | Authentic standards of MS-DIAL | 6.7 | 465.1015 | [M]+ | C21H21O12+ | 465.1033 | 2-[5,7-Dihydroxy-2-(3,4,5-Trihydroxyphenyl)Chromenylium-3-Yl]Oxy-6-(Hydroxymethyl)Oxane-3,4,5-Triol | Flavonoid glycosides | Level 2 | 0.93 | No |
| 77 | Vaniya- Fiehn_Natural_Products_Library | 7.4 | 551.1091 | [M+H]+ | C24H22O15 | 550.0959 | 3-[[6-[2-(3,4-Dihydroxyphenyl)-5,7-Dihydroxy-4-Oxochromen-3-Yl]Oxy-3,4,5-Trihydroxyoxan-2-Yl]Methoxy]-3-Oxopropanoic Acid | Flavonoid glycosides | Level 2 | 0.83 | No |
| 78 | Massbank | 5.4 | 579.1720 | [M-H]- | C27H32O14 | 580.1792 | 7-[4,5-Dihydroxy-6-(Hydroxymethyl)-3-(3,4,5-Trihydroxy-6-Methyloxan-2-Yl)Oxyoxan-2-Yl]Oxy-5-Hydroxy-2-(4-Hydroxyphenyl)-2,3-Dihydrochromen-4-One | Flavonoid glycosides | Level 2 | 0.66 | No |
| 79 | Vaniya- Fiehn_Natural_Products_Library | 6.3 | 593.1494 | [M-H]- | C27H30O15 | 594.1585 | Lonicerin | Flavonoid glycosides | Level 1 | 0.94 | Yes |
| 80 | Authentic standards of MS-DIAL | 7.4 | 595.1641 | [M+H]+ | C27H30O15 | 594.1585 | Biorobin | Flavonoid glycosides | Level 2 | 0.92 | No |
| 81 | Massbank | 7.9 | 593.1847 | [M-H]- | C28H34O14 | 594.1949 | 1-[4-[4,5-Dihydroxy-6-(Hydroxymethyl)-3-(3,4,5-Trihydroxy-6-Methyloxan-2-Yl)Oxyoxan-2-Yl]Oxy-2,6-Dihydroxyphenyl]-3-(4-Methoxyphenyl)Prop-2-En-1-One | Flavonoid glycosides | Level 2 | 0.65 | No |
| 82 | Respect | 7.1 | 595.1641 | [M]+ | C27H31O15+ | 595.1663 | 2-[[6-[2-(3,4-Dihydroxyphenyl)-5,7-Dihydroxychromenylium-3-Yl]Oxy-3,4,5-Trihydroxyoxan-2-Yl]Methoxy]-6-Methyloxane-3,4,5-Triol | Flavonoid glycosides | Level 2 | 0.87 | No |
| 83 | Massbank | 5.3 | 595.1657 | [M-H]- | C27H32O15 | 596.1741 | 2,6-Dihydroxy-2-[(4-Hydroxyphenyl)Methyl]-4-[3,4,5-Trihydroxy-6-[(3,4,5-Trihydroxy-6-Methyloxan-2-Yl)Oxymethyl]Oxan-2-Yl]Oxy-1-Benzofuran-3-One | Flavonoid glycosides | Level 2 | 0.64 | Yes |
| 84 | Vaniya- Fiehn_Natural_Products_Library | 6.6 | 611.1599 | [M+H]+ | C27H30O16 | 610.1534 | Rutin | Flavonoid glycosides | Level 1 | 0.91 | No |
| 85 | Authentic standards of MS-DIAL | 7.6 | 625.1719 | [M+H]+ | C28H32O16 | 624.1690 | 5,7-Dihydroxy-2-(4-Hydroxy-3-Methoxyphenyl)-3-[3,4,5-Trihydroxy-6-[(3,4,5-Trihydroxy-6-Methyloxan-2-Yl)Oxymethyl]Oxan-2-Yl]Oxychromen-4-One | Flavonoid glycosides | Level 2 | 0.90 | No |
| 86 | COCONUT | 8.6 | 731.2876 | [M-H]- | C37H48O15 | 732.2993 | 5-Hydroxy-2-[4-Hydroxy-3-(3-Methylbut-2-Enyl)Phenyl]-8-(3-Methylbut-2-Enyl)-7-[3,4,5-Trihydroxy-6-(Hydroxymethyl)Oxan-2-Yl]Oxy-3-(3,4,5-Trihydroxy-6-Methyloxan-2-Yl)Oxy-2,3-Dihydrochromen-4-One | Flavonoid glycosides | Level 3 | 0.61 | No |
| 87 | Vaniya- Fiehn_Natural_Products_Library | 6.6 | 741.2209 | [M+H]+ | C33H40O19 | 740.2164 | Mauritianin | Flavonoid glycosides | Level 1 | 0.96 | No |
| 88 | COCONUT | 7.6 | 745.2677 | [M-H]- | C37H46O16 | 746.2786 | 2-[3-(3,7-Dimethylocta-2,6-Dienyl)-4-Hydroxyphenyl]-5,7-Dihydroxy-8-[3,4,5-Trihydroxy-6-(Hydroxymethyl)Oxan-2-Yl]-3-[3,4,5-Trihydroxy-6-(Hydroxymethyl)Oxan-2-Yl]Oxychromen-4-One | Flavonoid glycosides | Level 3 | 0.54 | No |
| 89 | COCONUT | 7.5 | 747.2834 | [M-H]- | C37H48O16 | 748.2942 | 2-[3,4-Dihydroxy-5-(3-Methylbut-2-Enyl)Phenyl]-5-Hydroxy-8-(3-Methylbut-2-Enyl)-7-[3,4,5-Trihydroxy-6-(Hydroxymethyl)Oxan-2-Yl]Oxy-3-(3,4,5-Trihydroxy-6-Methyloxan-2-Yl)Oxy-2,3-Dihydrochromen-4-One | Flavonoid glycosides | Level 3 | 0.63 | No |
| 90 | COCONUT | 4.8 | 755.2007 | [M-H]- | C33H40O20 | 756.2113 | 7-[3,4-Dihydroxy-6-(Hydroxymethyl)-5-[3,4,5-Trihydroxy-6-(Hydroxymethyl)Oxan-2-Yl]Oxyoxan-2-Yl]Oxy-5-Hydroxy-2-[4-[3,4,5-Trihydroxy-6-(Hydroxymethyl)Oxan-2-Yl]Oxyphenyl]Chromen-4-One | Flavonoid glycosides | Level 3 | 0.52 | No |
| 91 | MONA | 4.4 | 773.2116 | [M+H]+ | C33H40O21 | 772.2062 | 3-[4,5-Dihydroxy-6-(Hydroxymethyl)-3-[3,4,5-Trihydroxy-6-(Hydroxymethyl)Oxan-2-Yl]Oxyoxan-2-Yl]Oxy-2-(3,4-Dihydroxyphenyl)-5-Hydroxy-7-(3,4,5-Trihydroxy-6-Methyloxan-2-Yl)Oxychromen-4-One | Flavonoid glycosides | Level 2 | 0.63 | No |
| 92 | COCONUT | 4.6 | 773.2111 | [M+H]+ | C33H40O21 | 772.2062 | Quercetin-O-Hexosyl-O-Hexosyl-Deoxyhexoside | Flavonoid glycosides | Level 3 | 0.58 | No |
| 93 | COCONUT | 7.7 | 805.2899 | [M-H]- | C39H50O18 | 806.2997 | 3-[4,5-Dihydroxy-6-Methyl-3-(3,4,5-Trihydroxy-6-Methyloxan-2-Yl)Oxyoxan-2-Yl]Oxy-2-(4-Methoxyphenyl)-8-(3-Methylbut-2-Enyl)-7-[3,4,5-Trihydroxy-6-(Hydroxymethyl)Oxan-2-Yl]Oxychromen-4-One | Flavonoid glycosides | Level 3 | 0.51 | No |
| 94 | COCONUT | 12.7 | 837.3123 | [M+H]+ | C40H52O19 | 836.3103 | 3-[3,4-Dihydroxy-5-Methyl-2-[3,4,5-Trihydroxy-6-(Hydroxymethyl)Oxan-2-Yl]Oxycyclohexyl]Oxy-5-Hydroxy-2-(4-Methoxyphenyl)-8-(3-Methylbut-2-Enyl)-7-[3,4,5-Trihydroxy-6-(Hydroxymethyl)Oxan-2-Yl]Oxychromen-4-One | Flavonoid glycosides | Level 3 | 0.78 | No |
| 95 | COCONUT | 6.7 | 921.3016 | [M-H]- | C43H54O22 | 922.3107 | 2-[(3-{[4,5-Dihydroxy-6-(Hydroxymethyl)-3-{[3,4,5-Trihydroxy-6-(Hydroxymethyl)Oxan-2-Yl]Oxy}Oxan-2-Yl]Oxy}-5-Hydroxy-2-(4-Hydroxyphenyl)-4-Oxo-4H-Chromen-7-Yl)Oxy]-4,5-Dihydroxy-6-Methyloxan-3-Yl 6-Hydroxy-2,6-Dimethylocta-2,7-Dienoate | Flavonoid glycosides | Level 3 | 0.54 | No |
| 96 | MONA | 7.0 | 461.1428 | [M+H]+ | C23H24O10 | 460.1369 | 6-Methoxy-3-(4-Methoxyphenyl)-7-[3,4,5-Trihydroxy-6-(Hydroxymethyl)Oxan-2-Yl]Oxychromen-4-One | Flavonoid glycosides | Level 2 | 0.65 | No |
| 97 | Vaniya- Fiehn_Natural_  Products_Library | 5.4 | 739.2041 | [M-H]- | C33H40O19 | 740.2164 | 7-[6-[[3,4-Dihydroxy-6-Methyl-5-[3,4,5-Trihydroxy-6-(Hydroxymethyl)Oxan-2-Yl]Oxyoxan-2-Yl]Oxymethyl]-3,4,5-Trihydroxyoxan-2-Yl]Oxy-5-Hydroxy-3-(4-Hydroxyphenyl)Chromen-4-One | Flavonoid glycosides | Level 2 | 0.91 | No |
| 98 | GNPS | 8.1 | 503.1896 | [M-H2O+H]+ | C26H32O11 | 520.1945 | 2-(Hydroxymethyl)-6-[4-[3-(Hydroxymethyl)-5-(3-Hydroxyprop-1-Enyl)-7-Methoxy-2,3-Dihydro-1-Benzofuran-2-Yl]-2-Methoxyphenoxy]Oxane-3,4,5-Triol | Flavonoid glycosides | Level 2 | 0.77 | Yes |
| 99 | Authentic standards of MS-DIAL | 7.3 | 540.2434 | [M+NH4]+ | C26H34O11 | 522.2101 | Isolariciresinol 9'-O-Beta-D-Glucoside | Flavonoid glycosides | Level 2 | 0.90 | No |
| 100 | COCONUT | 7.0 | 885.2452 | [M+H]+ | C42H44O21 | 884.2375 | 2-(3,4-Dihydroxyphenyl)-8-[2-(3,4-Dihydroxyphenyl)-7-Hydroxy-5-[3,4,5-Trihydroxy-6-(Hydroxymethyl)Oxan-2-Yl]Oxy-3,4-Dihydro-2H-Chromen-4-Yl]-7-Hydroxy-5-[3,4,5-Trihydroxy-6-(Hydroxymethyl)Oxan-2-Yl]Oxy-2,3-Dihydrochromen-4-One | Flavonoid glycosides | Level 3 | 0.63 | No |
| 101 | Massbank | 6.1 | 551.2098 | [M-H]- | C27H36O12 | 552.2207 | 2-[[7-Hydroxy-1-(4-Hydroxy-3,5-Dimethoxyphenyl)-3-(Hydroxymethyl)-6,8-Dimethoxy-1,2,3,4-Tetrahydronaphthalen-2-Yl]Methoxy]Oxane-3,4,5-Triol | Flavonoid glycosides | Level 2 | 0.62 | Yes |
| 102 | Authentic standards of MS-DIAL | 6.7 | 600.2655 | [M+NH4]+ | C28H38O13 | 582.2312 | Lyoniresinol 9'-O-Glucoside | Flavonoid glycosides | Level 2 | 0.80 | No |
| 103 | Massbank | 6.1 | 685.2703 | [M-H]- | C32H46O16 | 686.2786 | 2-[2,3-Bis[(4-Hydroxy-3-Methoxyphenyl)Methyl]-4-[3,4,5-Trihydroxy-6-(Hydroxymethyl)Oxan-2-Yl]Oxybutoxy]-6-(Hydroxymethyl)Oxane-3,4,5-Triol | Flavonoid glycosides | Level 2 | 0.66 | No |
| 104 | COCONUT | 7.8 | 787.2821 | [M-H]- | C39H48O17 | 788.2892 | (3,4,5-Trihydroxy-6-{[7-Hydroxy-1-(4-Hydroxy-3,5-Dimethoxyphenyl)-3-(Hydroxymethyl)-6,8-Dimethoxy-1,2,3,4-Tetrahydronaphthalen-2-Yl]Methoxy}Oxan-2-Yl)Methyl 3-(4-Hydroxy-3,5-Dimethoxyphenyl)Prop-2-Enoate | Flavonoid glycosides | Level 3 | 0.56 | No |
| 105 | COCONUT | 6.8 | 867.2328 | [M-H]- | C42H44O20 | 868.2426 | (2,4-Bis(4-Hydroxyphenyl)Cyclobutane-1,3-Diyl)Bis((2,4-Dihydroxy-6-((3,4,5-Trihydroxy-6-(Hydroxymethyl)Tetrahydro-2H-Pyran-2-Yl)Oxy)Phenyl)Methanone) | Others | Level 3 | 0.60 | No |
| 106 | MONA | 10.1 | 466.2633 | [M+NH4]+ | C21H36O10 | 448.2308 | Sacranoside B | Terpene glycosides | Level 2 | 0.60 | Yes |
| 107 | Authentic standards of MS-DIAL | 8.2 | 507.2421 | [M+CH3COO]- | C21H36O10 | 448.2308 | Bornyl 6-O-Beta-D-Xylopyranosyl-Beta-D-Glucopyranoside | Terpene glycosides | Level 2 | 0.86 | No |
| 108 | COCONUT | 5.0 | 349.1862 | [M-H]- | C16H30O8 | 350.1941 | 2-[2-Hydroxy-2-(3-Hydroxy-4-Methylcyclohexyl)Propoxy]-6-(Hydroxymethyl)Oxane-3,4,5-Triol | Terpene glycosides | Level 3 | 0.65 | No |
| 109 | Authentic standards of MS-DIAL | 7.4 | 533.1667 | [M-H2O+H]+ | C26H30O13 | 550.1686 | Jasminoside | Terpene glycosides | Level 2 | 0.71 | No |
| 110 | COCONUT | 13.4 | 491.2246 | [M+H]+ | C26H34O9 | 490.2203 | Beta-D-Glucosyl Crocetin | Terpene glycosides | Level 3 | 0.59 | No |
| 111 | COCONUT | 9.0 | 537.2316 | [M-H]- | C27H38O11 | 538.2414 | 15-Methoxy-2,6,14,17-Tetramethyl-4-{[3,4,5-Trihydroxy-6-(Hydroxymethyl)Oxan-2-Yl]Oxy}-10-Oxatetracyclo[7.7.1.0]heptadec-14-Ene-3,11,16-Trione | Terpene glycosides | Level 3 | 0.61 | No |
| 112 | GNPS | 8.1 | 505.2655 | [M-H2O+H]+ | C24H42O12 | 522.2676 | 2-[[3,4-Dihydroxy-4-(Hydroxymethyl)Oxolan-2-Yl]Oxymethyl]-6-[(6-Hydroxy-2,6,10,10-Tetramethyl-1-Oxaspiro[4.5]Decan-8-Yl)Oxy]Oxane-3,4,5-Triol | Terpene glycosides | Level 2 | 0.78 | No |
| 113 | COCONUT | 4.5 | 447.1856 | [M-H]- | C20H32O11 | 448.1945 | 3-[6-[[3,4-Dihydroxy-4-(Hydroxymethyl)Oxolan-2-Yl]Oxymethyl]-3,4,5-Trihydroxyoxan-2-Yl]Oxy-6,6-Dimethylbicyclo[3.1.1]Heptan-2-One | Terpene glycosides | Level 3 | 0.53 | No |
| 114 | COCONUT | 6.8 | 813.3145 | [M-H]- | C38H54O19 | 814.3259 | Crocin II | Terpene glycosides | Level 3 | 0.50 | Yes |
| 115 | Vaniya- Fiehn_Natural_Products_Library | 8.9 | 975.3677 | [M-H]- | C44H64O24 | 976.3788 | Crocin | Terpene glycosides | Level 2 | 0.90 | No |
| 116 | MONA | 6.5 | 373.2200 | [M+H]+ | C19H32O7 | 372.2148 | 3,5,5-Trimethyl-4-[3-[3,4,5-Trihydroxy-6-(Hydroxymethyl)Oxan-2-Yl]Oxybutyl]Cyclohex-2-En-1-One | Terpene glycosides | Level 2 | 0.64 | Yes |
| 117 | GNPS | 7.8 | 387.2011 | [M+H]+ | C19H30O8 | 386.1941 | Corchoionoside C | Terpene glycosides | Level 2 | 0.72 | No |
| 118 | Authentic standards of MS-DIAL | 6.0 | 389.2172 | [M+H]+ | C19H32O8 | 388.2097 | 4-Hydroxy-3,5,5-Trimethyl-4-[3-[3,4,5-Trihydroxy-6-(Hydroxymethyl)Oxan-2-Yl]Oxybutyl]Cyclohex-2-En-1-One | Terpene glycosides | Level 2 | 0.86 | No |
| 119 | COCONUT | 6.1 | 431.2277 | [M-H]- | C21H36O9 | 432.2359 | 2-{[4,5-Dihydroxy-2-(2-Hydroxypropan-2-Yl)-4A,8-Dimethyl-1,2,3,4,4A,5,6,8A-Octahydronaphthalen-1-Yl]Oxy}-6-(Hydroxymethyl)Oxane-3,4,5-Triol | Terpene glycosides | Level 3 | 0.61 | No |
| 120 | GNPS | 7.8 | 503.2475 | [M+H]+ | C24H38O11 | 502.2414 | 4-[3-[3-[3,4-Dihydroxy-4-(Hydroxymethyl)Oxolan-2-Yl]Oxy-4,5-Dihydroxy-6-(Hydroxymethyl)Oxan-2-Yl]Oxybut-1-Enyl]-3,5,5-Trimethylcyclohex-2-En-1-One | Terpene glycosides | Level 2 | 0.77 | No |
| 121 | Vaniya- Fiehn_Natural_  Products_Library | 6.8 | 350.2153 | [M+NH4]+ | C16H28O7 | 332.1835 | 2-(4-Hydroxy-3,7-Dimethylocta-2,6-Dienoxy)-6-(Hydroxymethyl)Oxane-3,4,5-Triol | Terpene glycosides | Level 2 | 0.72 | No |
| 122 | Authentic standards of MS-DIAL | 5.1 | 350.2170 | [M+NH4]+ | C16H28O7 | 332.1835 | 2-(Hydroxymethyl)-6-[4-(Hydroxymethyl)-1-Propan-2-Ylcyclohex-3-En-1-Yl]Oxyoxane-3,4,5-Triol | Terpene glycosides | Level 2 | 0.79 | No |
| 123 | Authentic standards of MS-DIAL | 4.6 | 391.1969 | [M+CH3COO]- | C16H28O7 | 332.1835 | 2-(Hydroxymethyl)-6-(6-Hydroxy-6-Methyl-3-Propan-2-Ylcyclohex-3-En-1-Yl)Oxyoxane-3,4,5-Triol | Terpene glycosides | Level 2 | 0.79 | Yes |
| 124 | Authentic standards of MS-DIAL | 6.0 | 405.1762 | [M+CH3COO]- | C16H26O8 | 346.1628 | Jasminoside D | Terpene glycosides | Level 2 | 0.87 | No |
| 125 | MONA | 3.1 | 385.1469 | [M+Na]+ | C16H26O9 | 362.1577 | 3-Hydroxy-2-[3-Methyl-2-[[3,4,5-Trihydroxy-6-(Hydroxymethyl)Oxan-2-Yl]Oxymethyl]Cyclopent-2-En-1-Yl]Propanoic Acid | Terpene glycosides | Level 2 | 0.65 | No |
| 126 | Authentic standards of MS-DIAL | 6.6 | 389.2190 | [M+H]+ | C19H32O8 | 388.2097 | 4-[4-Hydroxy-2,6,6-Trimethyl-3-[3,4,5-Trihydroxy-6-(Hydroxymethyl)Oxan-2-Yl]Oxycyclohexen-1-Yl]Butan-2-One | Terpene glycosides | Level 2 | 0.85 | Yes |
| 127 | GNPS | 7.4 | 431.2264 | [M+H]+ | C21H34O9 | 430.2203 | 5-Hydroxy-5-(2-Hydroxypropan-2-Yl)-3,8-Dimethyl-6-[3,4,5-Trihydroxy-6-(Hydroxymethyl)Oxan-2-Yl]Oxy-1,4,6,7,8,8A-Hexahydroazulen-2-One | Terpene glycosides | Level 2 | 0.82 | No |
| 128 | MONA | 7.0 | 433.2413 | [M-H2O+H]+ | C21H38O10 | 450.2465 | 2-[[5,8-Dihydroxy-4A-(Hydroxymethyl)-2-(2-Hydroxypropan-2-Yl)-8-Methyl-1,2,3,4,5,6,7,8A-Octahydronaphthalen-1-Yl]Oxy]-6-(Hydroxymethyl)Oxane-3,4,5-Triol | Terpene glycosides | Level 2 | 0.63 | No |
| 129 | Vaniya- Fiehn_Natural_Products_Library | 3.3 | 463.1455 | [M-H]- | C19H28O13 | 464.1530 | 2-[3-(1-Acetyloxyethyl)-5-Methoxycarbonyl-2-[3,4,5-Trihydroxy-6-(Hydroxymethyl)Oxan-2-Yl]Oxy-3,4-Dihydro-2H-Pyran-4-Yl]Acetic Acid | Terpene glycosides | Level 2 | 0.80 | No |
| 130 | COCONUT | 7.0 | 503.2472 | [M-H]- | C24H40O11 | 504.2571 | 4-{2,6,6-Trimethyl-4-[(3,4,5-Trihydroxy-6-{[(3,4,5-Trihydroxyoxan-2-Yl)Oxy]Methyl}Oxan-2-Yl)Oxy]Cyclohex-1-En-1-Yl}Butan-2-One | Terpene glycosides | Level 3 | 0.64 | No |
| 131 | COCONUT | 5.3 | 509.2216 | [M-H]- | C22H38O13 | 510.2312 | [3,4,5-Trihydroxy-6-(Hydroxymethyl)Oxan-2-Yl] 2,6-Dimethyl-8-[3,4,5-Trihydroxy-6-(Hydroxymethyl)Oxan-2-Yl]Oxyoct-2-Enoate | Terpene glycosides | Level 3 | 0.59 | Yes |
| 132 | MONA | 5.8 | 519.2412 | [M+H]+ | C24H38O12 | 518.2363 | 4-[(E)-3-[6-[[3,4-Dihydroxy-4-(Hydroxymethyl)Oxolan-2-Yl]Oxymethyl]-3,4,5-Trihydroxyoxan-2-Yl]Oxybut-1-Enyl]-4-Hydroxy-3,5,5-Trimethylcyclohex-2-En-1-One | Terpene glycosides | Level 2 | 0.62 | No |
| 133 | COCONUT | 6.7 | 533.2591 | [M-H]- | C25H42O12 | 534.2676 | 2-{[4-(3-Hydroxybut-1-En-1-Yl)-3,5,5-Trimethylcyclohex-3-En-1-Yl]Oxy}-6-({[3,4,5-Trihydroxy-6-(Hydroxymethyl)Oxan-2-Yl]Oxy}Methyl)Oxane-3,4,5-Triol | Terpene glycosides | Level 3 | 0.51 | No |
| 134 | COCONUT | 7.2 | 559.2740 | [M-H]- | C27H44O12 | 560.2833 | 2-(Hydroxymethyl)-6-{[6-Methyl-4-(1-{[3,4,5-Trihydroxy-6-(Hydroxymethyl)Oxan-2-Yl]Oxy}Propan-2-Yl)-2,3,4,4A,7,8-Hexahydronaphthalen-1-Yl]Methoxy}Oxane-3,4,5-Triol | Terpene glycosides | Level 3 | 0.61 | No |
| 135 | COCONUT | 6.8 | 595.2948 | [M-H]- | C27H48O14 | 596.3044 | 2-[[6-[[5,8-Dihydroxy-2-(2-Hydroxypropan-2-Yl)-4A,8-Dimethyl-1,2,3,4,5,6,7,8A-Octahydronaphthalen-1-Yl]Oxy]-3,4,5-Trihydroxyoxan-2-Yl]Methoxy]-6-(Hydroxymethyl)Oxane-3,4,5-Triol | Terpene glycosides | Level 3 | 0.62 | No |
| 136 | COCONUT | 9.8 | 649.3913 | [M-H]- | C36H58O10 | 650.4030 | 3,4,5-Trihydroxy-6-(Hydroxymethyl)Oxan-2-Yl 10,11-Dihydroxy-6A-(Hydroxymethyl)-2,2,6B,9,9,12A-Hexamethyl-1,2,3,4,4A,5,6,6A,6B,7,8,8A,9,10,11,12,12A,12B,13,14B-Icosahydropicene-4A-Carboxylate | Terpene glycosides | Level 3 | 0.64 | No |
| 137 | Massbank | 7.1 | 679.3624 | [M-H]- | C36H56O12 | 680.3772 | 2,3,12-Trihydroxy-4,6A,6B,11,12,14B-Hexamethyl-8A-[3,4,5-Trihydroxy-6-(Hydroxymethyl)Oxan-2-Yl]Oxycarbonyl-1,2,3,4A,5,6,7,8,9,10,11,12A,14,14A-Tetradecahydropicene-4-Carboxylic Acid | Terpene glycosides | Level 2 | 0.65 | No |
| 138 | Vaniya- Fiehn_Natural_Products_Library | 6.5 | 825.4271 | [M-H]- | C42H66O16 | 826.4351 | 3,12-Dihydroxy-4,6A,6B,11,12,14B-Hexamethyl-8A-[3,4,5-Trihydroxy-6-[[3,4,5-Trihydroxy-6-(Hydroxymethyl)Oxan-2-Yl]Oxymethyl]Oxan-2-Yl]Oxycarbonyl-1,2,3,4A,5,6,7,8,9,10,11,12A,14,14A-Tetradecahydropicene-4-Carboxylic Acid | Terpene glycosides | Level 2 | 0.72 | No |
| 139 | COCONUT | 23.6 | 935.5670 | [M+H]+ | C51H82O15 | 934.5654 | Methyl 6-{[4,4,6A,6B,11,11,14B-Heptamethyl-8A-({[3,4,5-Trihydroxy-6-(Hydroxymethyl)Oxan-2-Yl]Oxy}Carbonyl)-1,2,3,4,4A,5,6,6A,6B,7,8,8A,9,10,11,12,12A,14,14A,14B-Icosahydropicen-3-Yl]Oxy}-3,4-Dihydroxy-5-[(3,4,5-Trimethyloxan-2-Yl)Oxy]Oxane-2-Carboxylate | Terpene glycosides | Level 3 | 0.51 | No |
| 140 | COCONUT | 7.7 | 415.2319 | [M+H]+ | C21H34O8 | 414.2254 | 3,7-Dimethyl-6-[3-[3,4,5-Trihydroxy-6-(Hydroxymethyl)Oxan-2-Yl]Oxybutyl]-3,3A,4,7,8,8A-Hexahydrocyclohepta[B]Furan-2-One | Terpene glycosides | Level 3 | 0.56 | No |
| 141 | COCONUT | 6.9 | 687.2278 | [M-H]- | C34H40O15 | 688.2367 | [7-Hydroxy-7-Methyl-1-[3,4,5-Trihydroxy-6-(Hydroxymethyl)Oxan-2-Yl]Oxy-4A,5,6,7A-Tetrahydro-1H-Cyclopenta[C]Pyran-5-Yl] 3-[7-Hydroxy-2-(4-Hydroxy-3-Methoxyphenyl)-3-(Hydroxymethyl)-2,3-Dihydro-1-Benzofuran-5-Yl]Prop-2-Enoate | Terpene glycosides | Level 3 | 0.60 | No |
| 142 | COCONUT | 1.1 | 361.1498 | [M-H]- | C16H26O9 | 362.1577 | 2-({4A,5-Dihydroxy-4,7-Dimethyl-1H,4Ah,5H,6H,7H,7Ah-Cyclopenta[C]Pyran-1-Yl}Oxy)-6-(Hydroxymethyl)Oxane-3,4,5-Triol | Terpene glycosides | Level 3 | 0.62 | No |
| 143 | COCONUT | 6.7 | 699.2130 | [M-H]- | C31H40O18 | 700.2215 | [2-(Hydroxymethyl)-10-[3,4,5-Trihydroxy-6-(Hydroxymethyl)Oxan-2-Yl]Oxy-3,9-Dioxatricyclo[4.4.0.02,4]Dec-7-En-5-Yl] 3-[3-Methoxy-4-[3,4,5-Trihydroxy-6-(Hydroxymethyl)Oxan-2-Yl]Oxyphenyl]Prop-2-Enoate | Terpene glycosides | Level 3 | 0.65 | No |
| 144 | MONA | 4.3 | 531.1405 | [M+Na]+ | C24H28O12 | 508.1581 | [5-Hydroxy-10-[3,4,5-Trihydroxy-6-(Hydroxymethyl)Oxan-2-Yl]Oxy-3,9-Dioxatricyclo[4.4.0.02,4]Dec-7-En-2-Yl]Methyl 3-(4-Hydroxyphenyl)Prop-2-Enoate | Terpene glycosides | Level 2 | 0.76 | Yes |
| 145 | GNPS | 3.8 | 519.1495 | [M-H]- | C25H28O12 | 520.1581 | 2'-O-Cis-Coumaroylgardoside | Terpene glycosides | Level 2 | 0.88 | No |
| 146 | Authentic standards of MS-DIAL | 7.7 | 521.1636 | [M+H]+ | C25H28O12 | 520.1581 | 6-Hydroxy-7-Methylidene-1-[3,4,5-Trihydroxy-6-[3-(4-Hydroxyphenyl)Prop-2-Enoyloxymethyl]Oxan-2-Yl]Oxy-4A,5,6,7A-Tetrahydro-1H-Cyclopenta[C]Pyran-4-Carboxylic Acid | Terpene glycosides | Level 2 | 0.83 | No |
| 147 | Authentic standards of MS-DIAL | 4.7 | 487.1444 | [M-H]- | C21H28O13 | 488.1530 | 10-O-Succinoylgeniposide | Terpene glycosides | Level 2 | 0.88 | No |
| 148 | MONA | 6.1 | 477.1381 | [M-H2O+H]+ | C23H26O12 | 494.1424 | 6-Hydroxy-7-Methylidene-1-[3,4,5-Trihydroxy-6-[(4-Hydroxybenzoyl)Oxymethyl]Oxan-2-Yl]Oxy-4A,5,6,7A-Tetrahydro-1H-Cyclopenta[C]Pyran-4-Carboxylic Acid | Terpene glycosides | Level 2 | 0.71 | No |
| 149 | Vaniya- Fiehn_Natural_  Products_Library | 4.5 | 450.1949 | [M+NH4]+ | C19H28O11 | 432.1632 | 6-Ethoxygeniposide | Terpene glycosides | Level 2 | 0.74 | No |
| 150 | COCONUT | 5.0 | 357.1162 | [M+H]+ | C16H20O9 | 356.1107 | 5-Ethenyl-6-{[3,4,5-Trihydroxy-6-(Hydroxymethyl)Oxan-2-Yl]Oxy}-1H,3H,4H,6H-Pyrano[3,4-C]Pyran-1-One | Terpene glycosides | Level 3 | 0.67 | Yes |
| 151 | Authentic standards of MS-DIAL | 2.9 | 375.1266 | [M+H]+ | C16H22O10 | 374.1213 | Gardoside | Terpene glycosides | Level 2 | 0.75 | No |
| 152 | Vaniya- Fiehn_Natural_Products_Library | 3.8 | 359.1345 | [M-H]- | C16H24O9 | 360.1420 | 7-Methyl-1-[3,4,5-Trihydroxy-6-(Hydroxymethyl)Oxan-2-Yl]Oxy-1,4A,5,6,7,7A-Hexahydrocyclopenta[C]Pyran-4-Carboxylic Acid | Terpene glycosides | Level 2 | 0.81 | Yes |
| 153 | Authentic standards of MS-DIAL | 2.6 | 392.1527 | [M+NH4]+ | C16H22O10 | 374.1213 | Geniposidic Acid | Terpene glycosides | Level 2 | 0.73 | Yes |
| 154 | Authentic standards of MS-DIAL | 2.5 | 375.1285 | [M-H]- | C16H24O10 | 376.1369 | 8-Dehydroxyshanzhiside | Terpene glycosides | Level 2 | 0.93 | No |
| 155 | COCONUT | 3.1 | 389.1087 | [M+H]+ | C16H20O11 | 388.1006 | Ixoside | Terpene glycosides | Level 3 | 0.63 | Yes |
| 156 | FiehnHILIC | 5.0 | 387.1290 | [M-H]- | C17H24O10 | 388.1369 | Geniposide | Terpene glycosides | Level 1 | 0.95 | No |
| 157 | Authentic standards of MS-DIAL | 5.3 | 408.1825 | [M+NH4]+ | C17H26O10 | 390.1526 | Methyl 7-Hydroxy-7-Methyl-1-[3,4,5-Trihydroxy-6-(Hydroxymethyl)Oxan-2-Yl]Oxy-4A,5,6,7A-Tetrahydro-1H-Cyclopenta[C]Pyran-4-Carboxylate | Terpene glycosides | Level 2 | 0.70 | Yes |
| 158 | GNPS | 3.3 | 449.1286 | [M+FA-H]- | C17H24O11 | 404.1319 | Scandoside Methyl Ester | Terpene glycosides | Level 2 | 0.70 | Yes |
| 159 | Authentic standards of MS-DIAL | 1.7 | 463.1459 | [M+CH3COO]- | C17H24O11 | 404.1319 | Gardenoside | Terpene glycosides | Level 2 | 0.87 | Yes |
| 160 | Authentic standards of MS-DIAL | 4.2 | 407.1537 | [M+H]+ | C17H26O11 | 406.1475 | Shanzhiside Methyl Ester | Terpene glycosides | Level 2 | 0.71 | No |
| 161 | MONA | 6.1 | 469.1325 | [M+Na]+ | C19H26O12 | 446.1424 | Methyl 7-(Acetyloxymethyl)-5-Hydroxy-1-[3,4,5-Trihydroxy-6-(Hydroxymethyl)Oxan-2-Yl]Oxy-1,4A,5,7A-Tetrahydrocyclopenta[C]Pyran-4-Carboxylate | Terpene glycosides | Level 2 | 0.65 | No |
| 162 | MONA | 4.7 | 517.1652 | [M+Na]+ | C24H30O11 | 494.1788 | [4A,5-Dihydroxy-7-Methyl-1-[3,4,5-Trihydroxy-6-(Hydroxymethyl)Oxan-2-Yl]Oxy-1,5,6,7A-Tetrahydrocyclopenta[C]Pyran-7-Yl] 3-Phenylprop-2-Enoate | Terpene glycosides | Level 2 | 0.60 | No |
| 163 | MONA | 9.2 | 545.1645 | [M+Na]+ | C25H30O12 | 522.1737 | 6-[3-(4-Hydroxyphenyl)Prop-2-Enoyloxy]-7-Methyl-1-[3,4,5-Trihydroxy-6-(Hydroxymethyl)Oxan-2-Yl]Oxy-1,4A,5,6,7,7A-Hexahydrocyclopenta[C]Pyran-4-Carboxylic Acid | Terpene glycosides | Level 2 | 0.63 | No |
| 164 | COCONUT | 3.9 | 523.2003 | [M+H]+ | C22H34O14 | 522.1949 | [3,4,5-Trihydroxy-6-(Hydroxymethyl)Oxan-2-Yl] 7-Methyl-1-[3,4,5-Trihydroxy-6-(Hydroxymethyl)Oxan-2-Yl]Oxy-1,4A,5,6,7,7A-Hexahydrocyclopenta[C]Pyran-4-Carboxylate | Terpene glycosides | Level 3 | 0.66 | No |
| 165 | Authentic standards of MS-DIAL | 8.4 | 547.1789 | [M+Na]+ | C25H32O12 | 524.1894 | [4A,7-Dihydroxy-7-Methyl-1-[3,4,5-Trihydroxy-6-(Hydroxymethyl)Oxan-2-Yl]Oxy-1,5,6,7A-Tetrahydrocyclopenta[C]Pyran-5-Yl] 3-(4-Methoxyphenyl)Prop-2-Enoate | Terpene glycosides | Level 2 | 0.72 | No |
| 166 | GNPS | 7.4 | 533.1650 | [M-H]- | C26H30O12 | 534.1737 | Methyl 7-(Hydroxymethyl)-1-[3,4,5-Trihydroxy-6-[3-(4-Hydroxyphenyl)Prop-2-Enoyloxymethyl]Oxan-2-Yl]Oxy-1,4A,5,7A-Tetrahydrocyclopenta[C]Pyran-4-Carboxylate | Terpene glycosides | Level 2 | 0.84 | No |
| 167 | GNPS | 3.6 | 537.1597 | [M-H]- | C25H30O13 | 538.1686 | 1-[4,5-Dihydroxy-6-(Hydroxymethyl)-3-[3-(4-Hydroxyphenyl)Prop-2-Enoyloxy]Oxan-2-Yl]Oxy-5,7-Dihydroxy-7-Methyl-4A,5,6,7A-Tetrahydro-1H-Cyclopenta[C]Pyran-4-Carboxylic Acid | Terpene glycosides | Level 2 | 0.83 | No |
| 168 | Massbank | 4.0 | 537.1623 | [M-H]- | C25H30O13 | 538.1686 | 1-[6-[3-(3,4-Dihydroxyphenyl)Prop-2-Enoyloxymethyl]-3,4,5-Trihydroxyoxan-2-Yl]Oxy-7-Hydroxy-7-Methyl-4A,5,6,7A-Tetrahydro-1H-Cyclopenta[C]Pyran-4-Carboxylic Acid | Terpene glycosides | Level 2 | 0.63 | No |
| 169 | Authentic standards of MS-DIAL | 4.9 | 551.1966 | [M+H]+ | C23H34O15 | 550.1898 | Genipin 1-Gentiobioside | Terpene glycosides | Level 1 | 0.85 | No |
| 170 | Massbank | 5.1 | 567.1680 | [M-H]- | C26H32O14 | 568.1792 | Methyl 4A,7-Dihydroxy-6-[3-(4-Hydroxyphenyl)Prop-2-Enoyloxy]-7-Methyl-1-[3,4,5-Trihydroxy-6-(Hydroxymethyl)Oxan-2-Yl]Oxy-1,5,6,7A-Tetrahydrocyclopenta[C]Pyran-4-Carboxylate | Terpene glycosides | Level 2 | 0.60 | Yes |
| 171 | Authentic standards of MS-DIAL | 9.2 | 595.2018 | [M+H]+ | C28H34O14 | 594.1949 | Methyl 7-(Hydroxymethyl)-1-[3,4,5-Trihydroxy-6-[3-(4-Hydroxy-3,5-Dimethoxyphenyl)Prop-2-Enoyloxymethyl]Oxan-2-Yl]Oxy-1,4A,5,7A-Tetrahydrocyclopenta[C]Pyran-4-Carboxylate | Terpene glycosides | Level 2 | 0.85 | No |
| 172 | Authentic standards of MS-DIAL | 7.4 | 599.1952 | [M+H]+ | C27H34O15 | 598.1898 | 5,7-Dihydroxy-7-Methyl-1-[3,4,5-Trihydroxy-6-[3-(4-Hydroxy-3,5-Dimethoxyphenyl)Prop-2-Enoyloxymethyl]Oxan-2-Yl]Oxy-4A,5,6,7A-Tetrahydro-1H-Cyclopenta[C]Pyran-4-Carboxylic Acid | Terpene glycosides | Level 2 | 0.83 | No |
| 173 | MONA | 8.4 | 617.1839 | [M+H]+ | C30H32O14 | 616.1792 | Methyl 4'-[1-[3-(4-Hydroxyphenyl)Prop-2-Enoyloxy]Ethyl]-5'-Oxo-1-[3,4,5-Trihydroxy-6-(Hydroxymethyl)Oxan-2-Yl]Oxyspiro[4A,7A-Dihydro-1H-Cyclopenta[C]Pyran-7,2'-Furan]-4-Carboxylate | Terpene glycosides | Level 2 | 0.68 | No |
| 174 | MONA | 8.5 | 691.2206 | [M+Na]+ | C31H40O16 | 668.2316 | [4,5-Dihydroxy-6-[[7-(Hydroxymethyl)-1-[3,4,5-Trihydroxy-6-(Hydroxymethyl)Oxan-2-Yl]Oxy-1,4A,5,7A-Tetrahydrocyclopenta[C]Pyran-5-Yl]Oxy]-2-Methyloxan-3-Yl] 3-(3-Hydroxy-4-Methoxyphenyl)Prop-2-Enoate | Terpene glycosides | Level 2 | 0.61 | Yes |
| 175 | Vaniya-Fiehn_Natural  _Products _Library | 8.7 | 714.2585 | [M+NH4]+ | C32H40O17 | 696.2265 | 6'-O-P-Coumaroylgenipin Gentiobioside | Terpene glycosides | Level 2 | 0.91 | No |
| 176 | Authentic standards of MS-DIAL | 8.4 | 757.2550 | [M+H]+ | C34H44O19 | 756.2477 | Methyl 7-(Hydroxymethyl)-1-[3,4,5-Trihydroxy-6-[[3,4,5-Trihydroxy-6-[3-(4-Hydroxy-3,5-Dimethoxyphenyl)Prop-2-Enoyloxymethyl]Oxan-2-Yl]Oxymethyl]Oxan-2-Yl]Oxy-1,4A,5,7A-Tetrahydrocyclopenta[C]Pyran-4-Carboxylate | Terpene glycosides | Level 2 | 0.79 | No |
| 177 | COCONUT | 3.1 | 359.1338 | [M-H]- | C16H24O9 | 360.1420 | 5-Ethenyl-6-{[3,4,5-Trihydroxy-6-(Hydroxymethyl)Oxan-2-Yl]Oxy}-Octahydropyrano[3,4-C]Pyran-1-One | Terpene glycosides | Level 3 | 0.53 | No |
| 178 | COCONUT | 1.1 | 391.1246 | [M-H]- | C16H24O11 | 392.1319 | Shanzhiside | Terpene glycosides | Level 3 | 0.63 | No |
| 179 | Vaniya- Fiehn_Natural_Products_Library | 3.4 | 403.1244 | [M-H]- | C17H24O11 | 404.1319 | 6Α-Methoxygeniposide | Terpene glycosides | Level 2 | 0.73 | No |
| 180 | Authentic standards of MS-DIAL | 6.8 | 579.2070 | [M-H]- | C28H36O13 | 580.2156 | Tortoside A | Lignan glycosides | Level 1 | 0.91 | Yes |
| 181 | COCONUT | 6.3 | 595.2001 | [M-H]- | C28H36O14 | 596.2105 | 2-[[3,6-Bis(4-Hydroxy-3,5-Dimethoxyphenyl)-3,4,6,6A-Tetrahydro-1H-Furo[3,4-C]Furan-3A-Yl]Oxy]-6-(Hydroxymethyl)Oxane-3,4,5-Triol | Lignan glycosides | Level 3 | 0.61 | No |
| 182 | COCONUT | 8.5 | 681.2379 | [M-H]- | C32H42O16 | 682.2473 | 3-[(4-Hydroxy-3-Methoxyphenyl)Methyl]-4-[[3-Methoxy-4-[3,4,5-Trihydroxy-6-[[3,4,5-Trihydroxy-6-(Hydroxymethyl)Oxan-2-Yl]Oxymethyl]Oxan-2-Yl]Oxyphenyl]Methyl]Oxolan-2-One | Lignan glycosides | Level 3 | 0.61 | No |
| 183 | COCONUT | 12.1 | 697.2473 | [M+H]+ | C36H40O14 | 696.2418 | [3,4,5-Trihydroxy-6-[4-[3-(4-Hydroxy-3-Methoxyphenyl)-1,3,3A,4,6,6A-Hexahydrofuro[3,4-C]Furan-6-Yl]-2-Methoxyphenoxy]Oxan-2-Yl]Methyl 3-(4-Hydroxy-3-Methoxyphenyl)Prop-2-Enoate | Lignan glycosides | Level 3 | 0.61 | No |
| 184 | COCONUT | 8.5 | 697.2506 | [M-H]- | C36H42O14 | 698.2575 | (3,4,5-Trihydroxy-6-{[2-(4-Hydroxy-3-Methoxyphenyl)-4-[(4-Hydroxy-3-Methoxyphenyl)Methyl]Oxolan-3-Yl]Methoxy}Oxan-2-Yl)Methyl 3-(4-Hydroxy-3-Methoxyphenyl)Prop-2-Enoate | Lignan glycosides | Level 3 | 0.53 | No |
| 185 | Authentic standards of MS-DIAL | 6.6 | 760.2997 | [M+NH4]+ | C34H46O18 | 742.2684 | Eleutheroside E | Lignan glycosides | Level 2 | 0.76 | No |
| 186 | COCONUT | 6.6 | 581.2224 | [M-H]- | C28H38O13 | 582.2312 | Alangilignoside C | Lignan glycosides | Level 3 | 0.59 | No |
| 187 | COCONUT | 6.1 | 743.2725 | [M-H]- | C34H48O18 | 744.2841 | 2-{[7-Hydroxy-1-(4-Hydroxy-3,5-Dimethoxyphenyl)-6,8-Dimethoxy-3-({[3,4,5-Trihydroxy-6-(Hydroxymethyl)Oxan-2-Yl]Oxy}Methyl)-1,2,3,4-Tetrahydronaphthalen-2-Yl]Methoxy}-6-(Hydroxymethyl)Oxane-3,4,5-Triol | Lignan glycosides | Level 3 | 0.51 | No |
| 188 | COCONUT | 6.1 | 583.2372 | [M-H]- | C28H40O13 | 584.2469 | 2-({4-[4-(4-Hydroxy-3,5-Dimethoxycyclohexa-1,5-Dien-1-Yl)-Hexahydrofuro[3,4-C]Furan-1-Yl]-2,6-Dimethoxycyclohexa-2,4-Dien-1-Yl}Oxy)-6-(Hydroxymethyl)Oxane-3,4,5-Triol | Lignan glycosides | Level 3 | 0.57 | No |
| 189 | COCONUT | 6.9 | 907.3323 | [M-H]- | C43H56O21 | 908.3314 | 2-[4-(2-{2,6-Dimethoxy-4-[4-(3-Methoxy-4-{[3,4,5-Trihydroxy-6-(Hydroxymethyl)Oxan-2-Yl]Oxy}Phenyl)-Hexahydrofuro[3,4-C]Furan-1-Yl]Phenoxy}-1,3-Dihydroxypropyl)-2-Methoxyphenoxy]-6-(Hydroxymethyl)Oxane-3,4,5-Triol | Lignan glycosides | Level 3 | 0.60 | No |
| 190 | MONA | 13.3 | 513.3023 | [M+H]+ | C27H44O9 | 512.2985 | [2-Hydroxy-3-[3,4,5-Trihydroxy-6-(Hydroxymethyl)Oxan-2-Yl]Oxypropyl] Octadeca-6,9,12,15-Tetraenoate | Glycosylglycerols | Level 2 | 0.66 | No |
| 191 | Authentic standards of MS-DIAL | 18.4 | 532.3465 | [M+NH4]+ | C27H46O9 | 514.3142 | [2-Hydroxy-3-[3,4,5-Trihydroxy-6-(Hydroxymethyl)Oxan-2-Yl]Oxypropyl] Octadeca-9,12,15-Trienoate | Glycosylglycerols | Level 2 | 0.76 | No |
| 192 | COCONUT | 17.7 | 517.3329 | [M+H]+ | C27H48O9 | 516.3298 | 2-Hydroxy-3-{[3,4,5-Trihydroxy-6-(Hydroxymethyl)Oxan-2-Yl]Oxy}Propyl Octadeca-2,9-Dienoate | Glycosylglycerols | Level 3 | 0.67 | No |
| 193 | COCONUT | 26.8 | 751.5388 | [M+H]+ | C43H74O10 | 750.5282 | [2-Hexadec-9-Enoyloxy-3-[3,4,5-Trihydroxy-6-(Hydroxymethyl)Oxan-2-Yl]Oxypropyl] Octadeca-9,12,15-Trienoate | Glycosylglycerols | Level 3 | 0.50 | No |
| 194 | COCONUT | 21.9 | 771.4997 | [M+H]+ | C45H70O10 | 770.4969 | 2-(Hexadeca-6,9,12-Trienoyloxy)-3-{[3,4,5-Trihydroxy-6-(Hydroxymethyl)Oxan-2-Yl]Oxy}Propyl Icosa-5,8,11,14,17-Pentaenoate | Glycosylglycerols | Level 3 | 0.57 | No |
| 195 | COCONUT | 23.6 | 773.5142 | [M+H]+ | C45H72O10 | 772.5125 | [2-Octadeca-6,9,12,15-Tetraenoyloxy-3-[3,4,5-Trihydroxy-6-(Hydroxymethyl)Oxan-2-Yl]Oxypropyl] Octadeca-9,12,15-Trienoate | Glycosylglycerols | Level 3 | 0.53 | No |
| 196 | COCONUT | 23.3 | 775.5298 | [M+H]+ | C45H74O10 | 774.5282 | 3-(Octadeca-9,12,15-Trienoyloxy)-2-{[3,4,5-Trihydroxy-6-(Hydroxymethyl)Oxan-2-Yl]Oxy}Propyl Octadeca-9,12,15-Trienoate | Glycosylglycerols | Level 3 | 0.91 | No |
| 197 | COCONUT | 28.4 | 777.5466 | [M+H]+ | C45H76O10 | 776.5438 | 2-(Octadeca-9,12-Dienoyloxy)-3-{[3,4,5-Trihydroxy-6-(Hydroxymethyl)Oxan-2-Yl]Oxy}Propyl Octadeca-9,12,15-Trienoate | Glycosylglycerols | Level 3 | 0.70 | No |
| 198 | COCONUT | 26.8 | 781.5749 | [M+H]+ | C41H80O13 | 780.5599 | 2-(Octadecanoyloxy)-3-{[3,4,5-Trihydroxy-6-(Hydroxymethyl)Oxan-2-Yl]Oxy}Propyl Octadeca-9,12,15-Trienoate | Glycosylglycerols | Level 3 | 0.57 | No |
| 199 | COCONUT | 25.1 | 913.5829 | [M+H]+ | C49H84O15 | 912.5810 | [2-Hexadeca-7,10-Dienoyloxy-3-[3,4,5-Trihydroxy-6-[[3,4,5-Trihydroxy-6-(Hydroxymethyl)Oxan-2-Yl]Oxymethyl]Oxan-2-Yl]Oxypropyl] Octadeca-9,12-Dienoate | Glycosylglycerols | Level 3 | 0.58 | No |
| 200 | COCONUT | 17.7 | 931.5362 | [M+H]+ | C51H78O15 | 930.5341 | [1-Octadeca-3,6,9,12,15-Pentaenoyloxy-3-[3,4,5-Trihydroxy-6-[[3,4,5-Trihydroxy-6-(Hydroxymethyl)Oxan-2-Yl]Oxymethyl]Oxan-2-Yl]Oxypropan-2-Yl] Octadeca-6,9,12,15-Tetraenoate | Glycosylglycerols | Level 3 | 0.81 | No |
| 201 | COCONUT | 19.1 | 728.5277 | [M-H]- | C40H75NO10 | 729.5391 | N-((5E,12E)-3,4-Dihydroxy-1-((3,4,5-Trihydroxy-6-(Hydroxymethyl)Tetrahydro-2H-Pyran-2-Yl)Oxy)Octadeca-5,12-Dien-2-Yl)-2-Hydroxyhexadecanamide | Glycosphingolipids | Level 3 | 0.66 | No |
| 202 | COCONUT | 28.6 | 698.5532 | [M+H]+ | C40H75NO8 | 697.5493 | N-(3-Hydroxy-1-{[3,4,5-Trihydroxy-6-(Hydroxymethyl)Oxan-2-Yl]Oxy}Octadeca-4,8-Dien-2-Yl)Hexadecanamide | Glycosphingolipids | Level 3 | 0.57 | No |
| 203 | COCONUT | 29.1 | 710.5532 | [M+H]+ | C41H75NO8 | 709.5493 | N-[3-Hydroxy-9-Methyl-1-[3,4,5-Trihydroxy-6-(Hydroxymethyl)Oxan-2-Yl]Oxyoctadeca-4,8,10-Trien-2-Yl]Hexadecanamide | Glycosphingolipids | Level 3 | 0.68 | No |
| 204 | COCONUT | 24.6 | 712.5295 | [M+H]+ | C40H73NO9 | 711.5285 | Chrysogeside A | Glycosphingolipids | Level 3 | 0.64 | No |
| 205 | COCONUT | 22.4 | 712.5317 | [M-H]- | C40H75NO9 | 713.5442 | 2-Hydroxy-N-(3-Hydroxy-9-Methyl-1-{[3,4,5-Trihydroxy-6-(Hydroxymethyl)Oxan-2-Yl]Oxy}Octadeca-4,8-Dien-2-Yl)Pentadecanamide | Glycosphingolipids | Level 3 | 0.64 | No |
| 206 | COCONUT | 28.9 | 760.5873 | [M+H]+ | C42H81NO10 | 759.5860 | N-[3,4-Dihydroxy-1-[3,4,5-Trihydroxy-6-(Hydroxymethyl)Oxan-2-Yl]Oxynonadec-6-En-2-Yl]-2-Hydroxyheptadecanamide | Glycosphingolipids | Level 3 | 0.50 | No |
| 207 | COCONUT | 28.1 | 768.6005 | [M+H]+ | C44H81NO9 | 767.5911 | 2-Hydroxy-N-[3-Hydroxy-9-Methyl-1-[3,4,5-Trihydroxy-6-(Hydroxymethyl)Oxan-2-Yl]Oxyoctadeca-4,8,10-Trien-2-Yl]Nonadecanamide | Glycosphingolipids | Level 3 | 0.56 | No |
| 208 | COCONUT | 19.3 | 856.6816 | [M-H]- | C49H95NO10 | 857.6956 | N-(3,4-Dihydroxy-16-Methyl-1-{[3,4,5-Trihydroxy-6-(Hydroxymethyl)Oxan-2-Yl]Oxy}Heptadecan-2-Yl)-2-Hydroxypentacos-16-Enamide | Glycosphingolipids | Level 3 | 0.54 | No |
| 209 | COCONUT | 27.2 | 716.5639 | [M+H]+ | C40H77NO9 | 715.5598 | N-(3,4-Dihydroxy-1-{[3,4,5-Trihydroxy-6-(Hydroxymethyl)Oxan-2-Yl]Oxy}Octadec-8-En-2-Yl)Hexadecanamide | 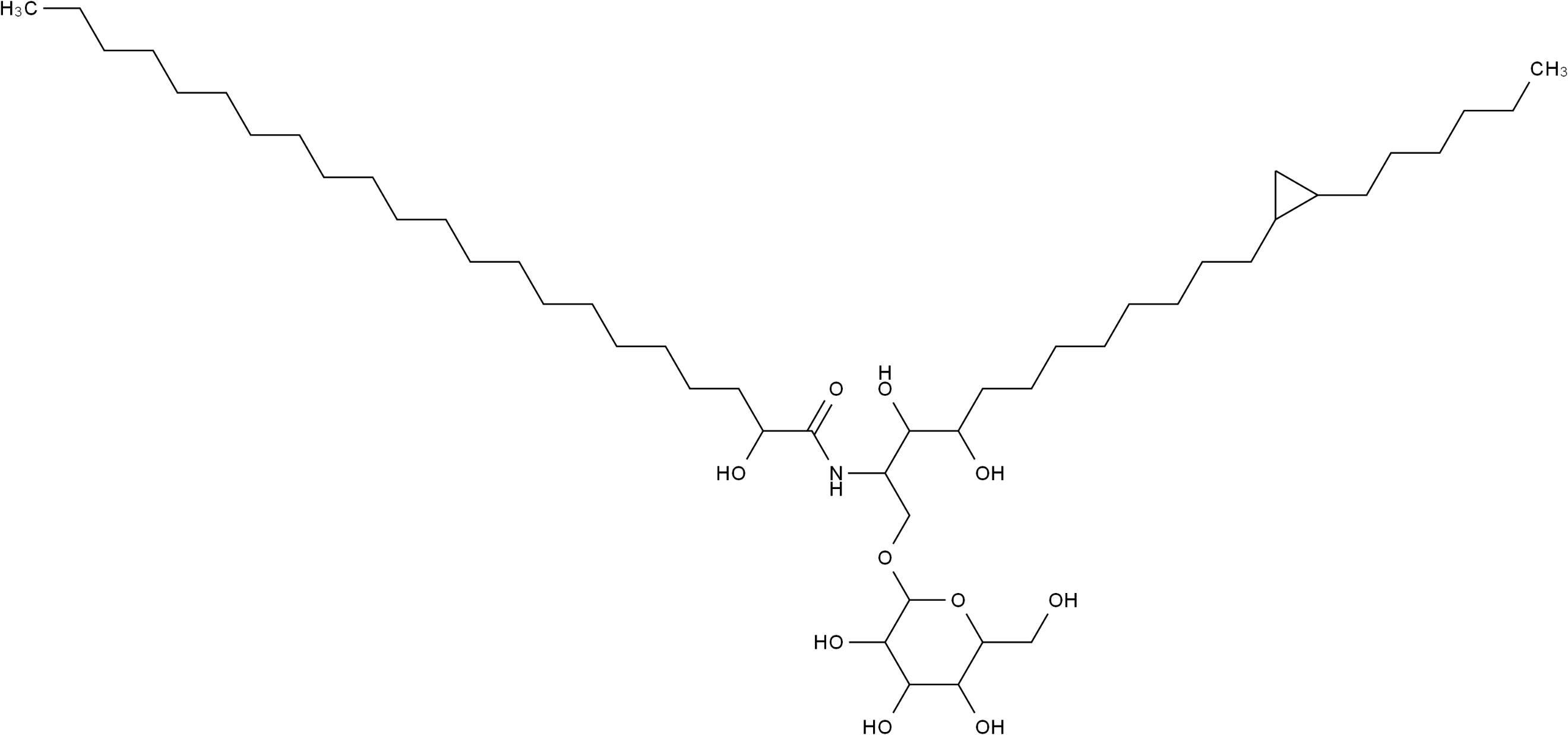   \| Glycosphingolipids \| \| --- \| | Level 3 | 0.56 | No |
| 210 | COCONUT | 28.4 | 740.5651 | [M+H]+ | C42H77NO9 | 739.5598 | Alternaroside C | Glycosphingolipids | Level 3 | 0.68 | No |
| 211 | Authentic standards of MS-DIAL | 12.9 | 638.3248 | [M-H]- | C29H54NO12P | 639.3384 | [1-Hydroxy-3-[Hydroxy-[2-[[3,4,5-Trihydroxy-6-(Hydroxymethyl)Oxan-2-Yl]Amino]Ethoxy]Phosphoryl]Oxypropan-2-Yl] Octadeca-9,12-Dienoate | Glycerophospholipids | Level 2 | 0.81 | No |
| 212 | Authentic standards of MS-DIAL | 18.9 | 833.5120 | [M-H]- | C43H79O13P | 834.5258 | [1-Hexadecanoyloxy-3-[Hydroxy-(2,3,4,5,6-Pentahydroxycyclohexyl)Oxyphosphoryl]Oxypropan-2-Yl] Octadeca-9,12-Dienoate | Glycerophospholipids | Level 2 | 0.91 | No |
| 213 | Authentic standards of MS-DIAL | 19.2 | 847.5250 | [M-H]- | C44H81O13P | 848.5415 | [1-Heptadecanoyloxy-3-[Hydroxy-(2,3,4,5,6-Pentahydroxycyclohexyl)Oxyphosphoryl]Oxypropan-2-Yl] Octadeca-9,12-Dienoate | Glycerophospholipids | Level 2 | 0.75 | No |
| 214 | Authentic standards of MS-DIAL | 17.1 | 849.5111 | [M-H]- | C43H79O14P | 850.5207 | [1-Hexadecanoyloxy-3-[Hydroxy-(2,3,4,5,6-Pentahydroxycyclohexyl)Oxyphosphoryl]Oxypropan-2-Yl] 9-Hydroxyoctadeca-10,12-Dienoate | Glycerophospholipids | Level 2 | 0.88 | No |
| 215 | Authentic standards of MS-DIAL | 19.5 | 861.5428 | [M-H]- | C45H83O13P | 862.5571 | [3-[Hydroxy-(2,3,4,5,6-Pentahydroxycyclohexyl)Oxyphosphoryl]Oxy-2-Octadeca-9,12-Dienoyloxypropyl] Octadecanoate | Glycerophospholipids | Level 2 | 0.87 | No |
| 216 | Authentic standards of MS-DIAL | 19.1 | 859.5258 | [M-H]- | C45H81O13P | 860.5415 | [3-[Hydroxy-(2,3,4,5,6-Pentahydroxycyclohexyl)Oxyphosphoryl]Oxy-2-Octadeca-9,12-Dienoyloxypropyl] Octadec-9-Enoate | Glycerophospholipids | Level 2 | 0.83 | No |
| 217 | COCONUT | 20.4 | 933.5511 | [M+H]+ | C51H80O15 | 932.5497 | 2-[1-(7-{[4,5-Dihydroxy-6-(Hydroxymethyl)-3-{[3,4,5-Trihydroxy-6-(Hydroxymethyl)Oxan-2-Yl]Oxy}Oxan-2-Yl]Oxy}-9A-(Hydroxymethyl)-3A,3B,6,6-Tetramethyl-Hexadecahydro-1H-Cyclopenta[A]Phenanthren-1-Yl)-1-Hydroxy-5-Methylhex-4-En-1-Yl]-6-(Hydroxymethyl)Oxane-3,4,5-Triol | Steroids glycosides | Level 3 | 0.80 | No |
| 218 | MONA | 13.6 | 657.3450 | [M+K]+ | C35H54O9 | 618.3768 | 2-[[2-Hydroxy-22-(2-Hydroxypropan-2-Yl)-3,8,8,17,19-Pentamethyl-23,24-Dioxaheptacyclo[19.2.1.01,18.03,17.04,14.07,12.012,14]Tetracos-4-En-9-Yl]Oxy]Oxane-3,4,5-Triol | Steroids glycosides | Level 2 | 0.70 | No |
| 219 | COCONUT | 15.1 | 599.3165 | [M-H]- | C34H48O9 | 600.3298 | 20-Hydroxy-1-Oxowitha-2,5,24-Trienolide | Steroids glycosides | Level 3 | 0.65 | No |
| 220 | COCONUT | 13.3 | 603.3115 | [M+H]+ | C33H46O10 | 602.3091 | 2-[1-(1,6-Dihydroxy-13-Methyl-6,7,8,9,11,12,14,15,16,17-Decahydrocyclopenta[A]Phenanthren-17-Yl)Ethyl]-5-Methyl-4-[[3,4,5-Trihydroxy-6-(Hydroxymethyl)Oxan-2-Yl]Oxymethyl]-2,3-Dihydropyran-6-One | Steroids glycosides | Level 3 | 0.52 | No |
| 221 | COCONUT | 18.0 | 619.2796 | [M+H]+ | C32H42O12 | 618.2676 | Urginin B | Steroids glycosides | Level 3 | 0.74 | No |
| 222 | COCONUT | 9.5 | 641.3141 | [M-H]- | C32H50O13 | 642.3251 | 2-((3-Hydroxy-1-(Hydroxymethyl)-1,4A,7-Trimethyl-7-Vinyl-1,2,3,4,4A,4B,5,6,7,10A-Decahydrophenanthren-2-Yl)Oxy)-6-(((3,4,5-Trihydroxy-6-(Hydroxymethyl)Tetrahydro-2H-Pyran-2-Yl)Oxy)Methyl)Tetrahydro-2H-Pyran-3,4,5-Triol | Steroids glycosides | Level 3 | 0.52 | No |
| 223 | COCONUT | 13.4 | 657.3450 | [M+H]+ | C33H52O13 | 656.3408 | 1-(3A-Hydroxy-9A,11A-Dimethyl-7-{[3,4,5-Trihydroxy-6-({[3,4,5-Trihydroxy-6-(Hydroxymethyl)Oxan-2-Yl]Oxy}Methyl)Oxan-2-Yl]Oxy}-1H,2H,3H,3Ah,3Bh,4H,6H,7H,8H,9H,9Ah,9Bh,10H,11H,11Ah-Cyclopenta[A]Phenanthren-1-Yl)Ethan-1-One | Steroids glycosides | Level 3 | 0.56 | No |
| 224 | COCONUT | 14.9 | 671.3619 | [M+H]+ | C34H54O13 | 670.3564 | 7-{[4,5-Dihydroxy-6-(Hydroxymethyl)-3-{[3,4,5-Trihydroxy-6-(Hydroxymethyl)Oxan-2-Yl]Oxy}Oxan-2-Yl]Oxy}-4-Hydroxy-3A,3B,6,6,9A-Pentamethyl-3H,3Ah,3Bh,4H,5H,5Ah,6H,7H,8H,9H,9Ah,9Bh,10H,11H,11Ah-Cyclopenta[A]Phenanthren-8-One | Steroids glycosides | Level 3 | 0.53 | No |
| 225 | COCONUT | 19.8 | 717.4495 | [M+H]+ | C37H64O13 | 716.4347 | 1-{5-[(3,4-Dihydroxy-5-{[(3,4,5-Trihydroxyoxan-2-Yl)Oxy]Methyl}Oxolan-2-Yl)Oxy]-6-Methylheptan-2-Yl}-9A,11A-Dimethyl-Hexadecahydro-1H-Cyclopenta[A]Phenanthrene-3,3B,5,7-Tetrol | Steroids glycosides | Level 3 | 0.61 | No |
| 226 | COCONUT | 20.4 | 771.4955 | [M+H]+ | C41H70O13 | 770.4816 | 2-[4,5-Dihydroxy-2-[[12-Hydroxy-17-(2-Hydroxy-6-Methylhept-5-En-2-Yl)-4,4,8,14-Tetramethyl-1,2,3,5,6,7,9,10,11,12,13,15,16,17-Tetradecahydrocyclopenta[A]Phenanthren-3-Yl]Oxy]-6-(Hydroxymethyl)Oxan-3-Yl]Oxy-6-(Hydroxymethyl)Oxane-3,4,5-Triol | Steroids glycosides | Level 3 | 0.57 | No |
| 227 | COCONUT | 17.6 | 661.3761 | [M+H]+ | C33H56O13 | 660.3721 | 6-Methoxy-7,9,13-Trimethyl-6-(4-{[3,4,5-Trihydroxy-6-(Hydroxymethyl)Oxan-2-Yl]Oxy}Butyl)-5-Oxapentacyclo Icosane-14,15,16,17,18-Pentol | Steroids glycosides | Level 3 | 0.63 | No |
| 228 | COCONUT | 23.2 | 803.4821 | [M+H]+ | C41H70O15 | 802.4715 | 2-(Hydroxymethyl)-6-[(4,5,6-Trihydroxy-2-{14-Hydroxy-7,7,12,16-Tetramethyl-6-[(3,4,5-Trihydroxyoxan-2-Yl)Oxy]Pentacyclo[9.7.0.0]octadecan-15-Yl}-6-Methylheptan-3-Yl)Oxy]Oxane-3,4,5-Triol | Steroids glycosides | Level 3 | 0.71 | Yes |
| 229 | COCONUT | 9.3 | 987.3466 | [M-H]- | C48H60O22 | 988.3576 | 4-[3-(3,4-Dihydroxyphenyl)Prop-2-Enoyloxy]-1,3-Dihydroxy-5-[2,6,11,15-Tetramethyl-16-Oxo-16-[3,4,5-Trihydroxy-6-[[3,4,5-Trihydroxy-6-(Hydroxymethyl)Oxan-2-Yl]Oxymethyl]Oxan-2-Yl]Oxyhexadeca-2,4,6,8,10,12,14-Heptaenoyl]Oxycyclohexane-1-Carboxylic Acid | Others | Level 3 | 0.60 | No |
| 230 | MONA | 11.8 | 389.0846 | [M+Na]+ | C17H18O9 | 366.0951 | 3-[4-[3,4,5-Trihydroxy-6-(Hydroxymethyl)Oxan-2-Yl]Oxy-1-Benzofuran-5-Yl]Prop-2-Enoic Acid | Others | Level 2 | 0.63 | No |
| 231 | COCONUT | 6.2 | 454.1701 | [M-H]- | C21H29NO10 | 455.1791 | 2-[[3,4-Dihydroxy-4-(Hydroxymethyl)Oxolan-2-Yl]Oxymethyl]-6-[2-(1H-Indol-3-Yl)Ethoxy]Oxane-3,4,5-Triol | Others | Level 3 | 0.52 | No |
| 232 | Authentic standards of MS-DIAL | 6.8 | 535.1988 | [M+FA-H]- | C22H34O12 | 490.2050 | Jasminoside R | Others | Level 2 | 0.80 | Yes |
| 233 | Authentic standards of MS-DIAL | 8.8 | 510.2533 | [M+NH4]+ | C22H36O12 | 492.2207 | Jasminoside S | Others | Level 2 | 0.87 | No |
| 234 | Vaniya- Fiehn_Natural_Products_Library | 6.2 | 331.1743 | [M-H]- | C16H28O7 | 332.1835 | 2-(6-Hydroxy-2,6-Dimethylocta-2,7-Dienoxy)-6-(Hydroxymethyl)Oxane-3,4,5-Triol | Others | Level 2 | 0.89 | No |
| 235 | Authentic standards of MS-DIAL | 4.6 | 425.1649 | [M+FA-H]- | C16H28O10 | 380.1682 | 2-(3-Methylbut-2-Enoxy)-6-[(3,4,5-Trihydroxyoxan-2-Yl)Oxymethyl]Oxane-3,4,5-Triol | Others | Level 2 | 0.71 | No |
| 236 | COCONUT | 7.8 | 433.2055 | [M-H]- | C20H34O10 | 434.2152 | 2-(2,6-Dimethylhepta-1,5-Dienoxy)-6-[(3,4,5-Trihydroxyoxan-2-Yl)Oxymethyl]Oxane-3,4,5-Triol | Others | Level 3 | 0.50 | No |
| 237 | COCONUT | 5.7 | 343.1226 | [M+H]+ | C12H22O11 | 342.1162 | 2,4-Dihydroxy-2-(Hydroxymethyl)-5-[3,4,5-Trihydroxy-6-(Hydroxymethyl)Oxan-2-Yl]Oxypentanoic Acid | Others | Level 3 |  | No |
| 238 | COCONUT | 11.6 | 431.2634 | [M-H]- | C22H40O8 | 432.2723 | (3,4,5-Trihydroxy-6-Octanoyloxyoxan-2-Yl)Methyl Octanoate | Others | Level 3 | 0.60 | No |
| 239 | MONA | 7.7 | 527.2006 | [M+Na]+ | C23H36O12 | 504.2207 | 3-Hydroxy-3-Methyl-5-Oxo-5-[[3,4,5-Trihydroxy-6-(8-Methoxy-3,7-Dimethyl-8-Oxoocta-1,6-Dien-3-Yl)Oxyoxan-2-Yl]Methoxy]Pentanoic Acid | Others | Level 2 | 0.60 | No |
| 240 | Massbank | 11.1 | 505.3476 | [M-H]- | C26H50O9 | 506.3455 | 19-Hydroxy-18-[3,4,5-Trihydroxy-6-(Hydroxymethyl)Oxan-2-Yl]Oxyicosanoic Acid | Others | Level 2 | 0.66 | No |
| 241 | Massbank | 19.5 | 695.3110 | [M+HCOO]- | C30H50O15 | 650.3150 | [2-[4-Acetyloxy-3-Hydroxy-2,5-Bis(Hydroxymethyl)Oxolan-2-Yl]Oxy-6-(Hydroxymethyl)-4,5-Di(Pentanoyloxy)Oxan-3-Yl] Hexanoate | Others | Level 2 | 0.65 | No |
| 242 | COCONUT | 11.9 | ######## | [M+H]+ | C50H72O28 | ######## | Methyl 5-Ethylidene-4-[2-[8-[6-[[2-[3-Ethylidene-5-Methoxycarbonyl-2-[3,4,5-Trihydroxy-6-(Hydroxymethyl)Oxan-2-Yl]Oxy-4H-Pyran-4-Yl]Acetyl]Oxymethyl]-3,4,5-Trihydroxyoxan-2-Yl]Oxy-3,7-Dimethyl-8-Oxooct-6-Enoxy]-2-Oxoethyl]-6-[3,4,5-Trihydroxy-6-(Hydroxymethyl)Oxan-2-Yl]Oxy-4H-Pyran-3-Carboxylate | Others | Level 3 | 0.73 | Yes |
| 243 | COCONUT | 23.1 | 789.5141 | [M+H]+ | C45H72O11 | 788.5075 | 2-((8-(4-Oxo-5-((E)-Pent-2-En-1-Yl)Cyclopent-2-En-1-Yl)Octanoyl)Oxy)-3-((3,4,5-Trihydroxy-6-(Hydroxymethyl)Tetrahydro-2H-Pyran-2-Yl)Oxy)Propyl (9E,12E,15E)-Octadeca-9,12,15-Trienoate | Others | Level 3 | 0.88 | No |
| 244 | COCONUT | 1.0 | 343.1232 | [M-H]- | C12H24O11 | 344.1319 | 2-[[3,4,5-Trihydroxy-6-(Hydroxymethyl)Oxan-2-Yl]Oxymethyl]Pentane-1,2,3,4,5-Pentol | Others | Level 3 | 0.66 | No |
| 245 | Authentic standards of MS-DIAL | 1.6 | 294.1521 | [M+H]+ | C12H23NO7 | 293.1475 | 3-Methyl-2-[[2,3,4-Trihydroxy-5-(Hydroxymethyl)Oxolan-2-Yl]Methylamino]Pentanoic Acid | Others | Level 2 | 0.86 | No |
| 246 | Authentic standards of MS-DIAL | 5.4 | 377.1448 | [M+H]+ | C17H20N4O6 | 376.1383 | Riboflavin (B2) | Others | Level 1 | 0.94 | Yes |
| 247 | Massbank | 3.8 | 385.1249 | [M+H]+ | C14H20N6O5S | 384.1216 | 2-Amino-4-[[5-(6-Aminopurin-9-Yl)-3,4-Dihydroxyoxolan-2-Yl]Methylsulfanyl]Butanoic Acid | Others | Level 2 | 0.66 | No |
| 248 | Authentic standards of MS-DIAL | 1.2 | 204.0861 | [M-H2O+H]+ | C8H15NO6 | 221.0899 | N-[2,4,5-Trihydroxy-6-(Hydroxymethyl)Oxan-3-Yl]Acetamide | Others | Level 2 | 0.83 | No |
| 249 | Authentic standards of MS-DIAL | 9.5 | 295.1015 | [M+H]+ | C11H18O9 | 294.0951 | 4-Hydroxy-5-[[3,4,5-Trihydroxy-6-(Hydroxymethyl)Oxan-2-Yl]Oxymethyl]Oxolan-2-One | Others | Level 2 | 0.72 | No |
| 250 | COCONUT | 1.6 | 303.1438 | [M-H]- | C14H24O7 | 304.1522 | 5-[(5-Hydroxy-4-Methoxy-6-Methyloxan-2-Yl)Oxy]-4-Methoxy-6-Methyloxan-2-One | Others | Level 3 | 0.62 | Yes |
| 251 | GNPS | 6.4 | 331.1738 | [M+H]+ | C16H26O7 | 330.1679 | Epijasminoside A | Others | Level 2 | 0.90 | No |
| 252 | Authentic standards of MS-DIAL | 6.1 | 391.1960 | [M+CH3COO]- | C16H28O7 | 332.1835 | 2-(Hydroxymethyl)-6-[(1,3,3-Trimethyl-2-Oxabicyclo[2.2.2]Octan-6-Yl)Oxy]Oxane-3,4,5-Triol | Others | Level 2 | 0.84 | Yes |
| 253 | Authentic standards of MS-DIAL | 7.6 | 461.2025 | [M-H]- | C21H34O11 | 462.2101 | [3,4,5-Trihydroxy-6-[(3,4,5-Trihydroxyoxan-2-Yl)Oxymethyl]Oxan-2-Yl] 2,6,6-Trimethylcyclohexene-1-Carboxylate | Others | Level 2 | 0.89 | Yes |
| 254 | COCONUT | 5.6 | 461.2019 | [M-H]- | C21H34O11 | 462.2101 | Jasminoside T | Others | Level 3 | 0.63 | No |
| 255 | COCONUT | 2.6 | 565.1745 | [M-H]- | C23H34O16 | 566.1847 | Methyl 8-Methyl-6-Oxo-1-{[3,4,5-Trihydroxy-6-({[3,4,5-Trihydroxy-6-(Hydroxymethyl)Oxan-2-Yl]Oxy}Methyl)Oxan-2-Yl]Oxy}-1H,4Ah,5H,6H,8H,8Ah-Pyrano[3,4-C]Pyran-4-Carboxylate | Others | Level 3 | 0.77 | No |
| 256 | COCONUT | 3.8 | 458.1987 | [M+H]+ | C21H31NO10 | 457.1948 | (7-Oxo-5,6-Dihydropyrrolizin-1-Yl)Methyl 2-Hydroxy-2-Propan-2-Yl-3-[3,4,5-Trihydroxy-6-(Hydroxymethyl)Oxan-2-Yl]Oxybutanoate | Others | Level 3 | 0.64 | No |
| 257 | Authentic standards of MS-DIAL | 5.3 | 325.1375 | [M+H]+ | C15H20N2O6 | 324.1321 | 6,7-Dimethyl-4-(2,3,4,5-Tetrahydroxypentyl)-1H-Quinoxaline-2,3-Dione | Others | Level 2 | 0.84 | No |
| 258 | COCONUT | 6.1 | 614.2423 | [M+H]+ | C28H39NO14 | 613.2371 | 2-[[4-Hydroxy-5-Methoxy-15-[3,4,5-Trihydroxy-6-(Hydroxymethyl)Oxan-2-Yl]Oxy-9-Azatetracyclo[7.6.1.02,7.012,16]Hexadeca-2,4,6,12-Tetraen-14-Yl]Oxy]-6-(Hydroxymethyl)Oxane-3,4,5-Triol | Others | Level 3 | 0.50 | No |
| 259 | Authentic standards of MS-DIAL | 1.2 | 268.1035 | [M+H]+ | C10H13N5O4 | 267.0968 | 2-(6-Aminopurin-9-Yl)-5-(Hydroxymethyl)Oxolane-3,4-Diol | Others | Level 2 | 0.94 | No |
| 260 | Authentic standards of MS-DIAL | 4.4 | 413.1378 | [M+H]+ | C15H20N6O8 | 412.1343 | 2-[[9-[3,4-Dihydroxy-5-(Hydroxymethyl)Oxolan-2-Yl]Purin-6-Yl]Carbamoylamino]-3-Hydroxybutanoic Acid | Others | Level 2 | 0.78 | No |
| 261 | COCONUT | 6.9 | 430.1471 | [M+H]+ | C22H23NO8 | 429.1424 | 1-(2-Amino-1-Benzofuran-3-Yl)-2-(2-{[3,4,5-Trihydroxy-6-(Hydroxymethyl)Oxan-2-Yl]Oxy}Phenyl)Ethan-1-One | Others | Level 3 | 0.52 | No |
| 262 | COCONUT | 4.5 | 485.1289 | [M-H]- | C21H26O13 | 486.1373 | 5-[6-[[3,4-Dihydroxy-4-(Hydroxymethyl)Oxolan-2-Yl]Oxymethyl]-3,4,5-Trihydroxyoxan-2-Yl]Oxy-7-Hydroxy-2-Methylchromen-4-One | Others | Level 3 | 0.58 | No |
| 263 | MONA | 11.7 | 495.1460 | [M+H]+ | C23H26O12 | 494.1424 | [4,5-Diacetyloxy-3-Hydroxy-6-(5-Hydroxy-2,8-Dimethyl-4-Oxochromen-7-Yl)Oxyoxan-2-Yl]Methyl Acetate | Others | Level 2 | 0.68 | No |
| 264 | COCONUT | 6.1 | 591.2641 | [M-H]- | C27H44O14 | 592.2731 | 2-(4,6-Dihydroxy-2-Nonyl-3-{[3,4,5-Trihydroxy-6-(Hydroxymethyl)Oxan-2-Yl]Oxy}Phenoxy)-6-(Hydroxymethyl)Oxane-3,4,5-Triol | Others | Level 3 | 0.62 | No |
| 265 | COCONUT | 6.3 | 853.2778 | [M-H]- | C39H50O21 | 854.2845 | Pyrolaside B | Others | Level 3 | 0.54 | No |

MW* represents Molecular weight

Table S6 VIP values, FC values, and FDR-values of 21 glycosides with significant changes

| No | Database | tR/min | m/z | Adduct type | Formula | MW* | Name | Reported anti-inflammatory activity | FC | FDR | VIP |
| --- | --- | --- | --- | --- | --- | --- | --- | --- | --- | --- | --- |
| 38 | MassBank | 4.7 | 385.1132 | [M-H]- | C17H22O10 | 386.1213 | 1-O-Sinapoyl-beta-D-glucose | Yes | 3.4 | 0.0000753 | 3.6 |
| 48 | MSMS_Public_EXP_VS17 | 4.5 | 355.1015 | [M+H]+ | C16H18O9 | 354.0951 | Chlorogenic acid | Yes | 4.8 | 0.0001273 | 1.3 |
| 51 | Vaniya- Fiehn_Natural_  Products_Library | 5.2 | 515.1180 | [M-H]- | C25H24O12 | 516.1268 | Isochlorogenic acid B | Yes | 2.5 | 0.41101 | 1.7 |
| 54 | MSMS_Public_EXP_VS17 | 5.8 | 559.1454 | [M-H]- | C27H28O13 | 560.1530 | 3-O-Caffeoyl-4-O-sinapoylquinic acid | / | 7.0 | 4.85E-14 | 5.3 |
| 58 | COCONUT | 8.4 | 369.1173 | M+H | C17H20O9 | 368.1107 | 7-hydroxy-6-[2-[3,4,5-trihydroxy-6-(hydroxymethyl)oxan-2-yl]oxyethyl]chromen-2-one | / | 19.7 | 1.6E-12 | 1.9 |
| 69 | MSMS_Public_EXP_VS17 | 1.2 | 431.0952 | [M+H]+ | C21H18O10 | 430.0900 | 3,4,5-trihydroxy-6-(5-hydroxy-4-oxo-2-phenylchromen-7-yl)oxyoxane-2-carboxylic acid | / | 0.3 | 8.65E-07 | 1.2 |
| 78 | Massbank | 5.4 | 579.1720 | [M-H]- | C27H32O14 | 580.1792 | 7-[4,5-dihydroxy-6-(hydroxymethyl)-3-(3,4,5-trihydroxy-6-methyloxan-2-yl)oxyoxan-2-yl]oxy-5-hydroxy-2-(4-hydroxyphenyl)-2,3-dihydrochromen-4-one | / | 35.7 | 2.46E-16 | 4.4 |
| 79 | Vaniya- Fiehn_Natural_  Products_Library | 6.3 | 593.1494 | [M-H]- | C27H30O15 | 594.1585 | Lonicerin | Yes | 5.4 | 1.84E-10 | 1.0 |
| 81 | Massbank | 7.9 | 593.1847 | [M-H]- | C28H34O14 | 594.1949 | 1-[4-[4,5-dihydroxy-6-(hydroxymethyl)-3-(3,4,5-trihydroxy-6-methyloxan-2-yl)oxyoxan-2-yl]oxy-2,6-dihydroxyphenyl]-3-(4-methoxyphenyl)prop-2-en-1-one | / | 8.7 | 1.2E-14 | 4.0 |
| 83 | Massbank | 5.3 | 595.1657 | [M-H]- | C27H32O15 | 596.1741 | 2,6-dihydroxy-2-[(4-hydroxyphenyl)methyl]-4-[3,4,5-trihydroxy-6-[(3,4,5-trihydroxy-6-methyloxan-2-yl)oxymethyl]oxan-2-yl]oxy-1-benzofuran-3-one | / | 53.5 | 5.94E-15 | 2.2 |
| 84 | Vaniya- Fiehn_Natural_  Products_Library | 6.6 | 611.1599 | [M+H]+ | C27H30O16 | 610.1534 | Rutin | Yes | 7.6 | 7.62E-12 | 3.3 |
| 87 | Vaniya- Fiehn_Natural_  Products_Library | 6.6 | 741.2209 | [M+H]+ | C33H40O19 | 740.2164 | Mauritianin | / | 2.4 | 0.51111 | 1.1 |
| 127 | GNPS | 7.4 | 431.2264 | [M+H]+ | C21H34O9 | 430.2203 | 5-hydroxy-5-(2-hydroxypropan-2-yl)-3,8-dimethyl-6-[3,4,5-trihydroxy-6-(hydroxymethyl)oxan-2-yl]oxy-1,4,6,7,8,8a-hexahydroazulen-2-one | / | 0.3 | 2.63E-14 | 1.8 |
| 132 | MONA | 5.8 | 519.2412 | [M+H]+ | C24H38O12 | 518.2363 | 4-[(E)-3-[6-[[3,4-dihydroxy-4-(hydroxymethyl)oxolan-2-yl]oxymethyl]-3,4,5-trihydroxyoxan-2-yl]oxybut-1-enyl]-4-hydroxy-3,5,5-trimethylcyclohex-2-en-1-one | / | 0.4 | 2.55E-11 | 1.0 |
| 165 | MSMS_Public_EXP_VS17 | 8.4 | 547.1789 | [M+Na]+ | C25H32O12 | 524.1894 | [4a,7-dihydroxy-7-methyl-1-[3,4,5-trihydroxy-6-(hydroxymethyl)oxan-2-yl]oxy-1,5,6,7a-tetrahydrocyclopenta[c]pyran-5-yl] 3-(4-methoxyphenyl)prop-2-enoate | / | 4.6 | 0.0000021 | 1.6 |
| 171 | MSMS_Public_EXP_VS17 | 9.2 | 595.2018 | [M+H]+ | C28H34O14 | 594.1949 | methyl 7-(hydroxymethyl)-1-[3,4,5-trihydroxy-6-[3-(4-hydroxy-3,5-dimethoxyphenyl)prop-2-enoyloxymethyl]oxan-2-yl]oxy-1,4a,5,7a-tetrahydrocyclopenta[c]pyran-4-carboxylate | / | 4.6 | 2.54E-10 | 1.1 |
| 172 | MSMS_Public_EXP_VS17 | 7.4 | 599.1952 | [M+H]+ | C27H34O15 | 598.1898 | 5,7-dihydroxy-7-methyl-1-[3,4,5-trihydroxy-6-[3-(4-hydroxy-3,5-dimethoxyphenyl)prop-2-enoyloxymethyl]oxan-2-yl]oxy-4a,5,6,7a-tetrahydro-1H-cyclopenta[c]pyran-4-carboxylic acid | / | 9.8 | 2.12E-11 | 1.1 |
| 176 | MSMS_Public_EXP_VS17 | 8.4 | 757.2550 | [M+H]+ | C34H44O19 | 756.2477 | methyl 7-(hydroxymethyl)-1-[3,4,5-trihydroxy-6-[[3,4,5-trihydroxy-6-[3-(4-hydroxy-3,5-dimethoxyphenyl)prop-2-enoyloxymethyl]oxan-2-yl]oxymethyl]oxan-2-yl]oxy-1,4a,5,7a-tetrahydrocyclopenta[c]pyran-4-carboxylate | / | 0.4 | 2.32E-09 | 1.7 |
| 180 | MSMS_Public_EXP_VS17 | 6.8 | 579.2070 | [M-H]- | C28H36O13 | 580.2156 | Tortoside A | / | 35.9 | 3.74E-13 | 1.4 |
| 233 | MSMS_Public_EXP_VS17 | 8.8 | 510.2533 | [M+NH4]+ | C22H36O12 | 492.2207 | Jasminoside S | Yes | 0.2 | 2.46E-16 | 3.0 |
| 252 | MSMS_Public_EXP_VS17 | 6.1 | 391.1960 | [M+CH3COO]- | C16H28O7 | 332.1835 | 2-(hydroxymethyl)-6-[(1,3,3-trimethyl-2-oxabicyclo[2.2.2]octan-6-yl)oxy]oxane-3,4,5-triol | / | 7.5 | 8.56E-09 | 1.1 |

MW* represents Molecular weight

Table S7 The binding energy between compounds and the iNOS protein

| No | Database | t_R_/min | m/z | Adduct type | Formula | MW* | Name | Binding Eenergy (kcal/mol) |
| --- | --- | --- | --- | --- | --- | --- | --- | --- |
| 54 | MSMS_Public_EXP_VS17 | 5.8 | 559.1454 | [M-H]- | C27H28O13 | 560.1530 | 3-O-Caffeoyl-4-O-sinapoylquinic acid | -9.9 |
| 58 | COCONUT | 8.4 | 369.1173 | M+H | C17H20O9 | 368.1107 | 7-hydroxy-6-[2-[3,4,5-trihydroxy-6-(hydroxymethyl)oxan-2-yl]oxyethyl]chromen-2-one | -9.3 |
| 69 | MSMS_Public_EXP_VS17 | 1.2 | 431.0952 | [M+H]+ | C21H18O10 | 430.0900 | 3,4,5-trihydroxy-6-(5-hydroxy-4-oxo-2-phenylchromen-7-yl)oxyoxane-2-carboxylic acid | -11.0 |
| 78 | Massbank | 5.4 | 579.1720 | [M-H]- | C27H32O14 | 580.1792 | 7-[4,5-dihydroxy-6-(hydroxymethyl)-3-(3,4,5-trihydroxy-6-methyloxan-2-yl)oxyoxan-2-yl]oxy-5-hydroxy-2-(4-hydroxyphenyl)-2,3-dihydrochromen-4-one | -11.1 |
| 81 | Massbank | 7.9 | 593.1847 | [M-H]- | C28H34O14 | 594.1949 | 1-[4-[4,5-dihydroxy-6-(hydroxymethyl)-3-(3,4,5-trihydroxy-6-methyloxan-2-yl)oxyoxan-2-yl]oxy-2,6-dihydroxyphenyl]-3-(4-methoxyphenyl)prop-2-en-1-one | -10.3 |
| 83 | Massbank | 5.3 | 595.1657 | [M-H]- | C27H32O15 | 596.1741 | 2,6-dihydroxy-2-[(4-hydroxyphenyl)methyl]-4-[3,4,5-trihydroxy-6-[(3,4,5-trihydroxy-6-methyloxan-2-yl)oxymethyl]oxan-2-yl]oxy-1-benzofuran-3-one | -9.6 |
| 87 | Vaniya- Fiehn_Natural_  Products_Library | 6.6 | 741.2209 | [M+H]+ | C33H40O19 | 740.2164 | Mauritianin | -11.3 |
| 127 | GNPS | 7.4 | 431.2264 | [M+H]+ | C21H34O9 | 430.2203 | 5-hydroxy-5-(2-hydroxypropan-2-yl)-3,8-dimethyl-6-[3,4,5-trihydroxy-6-(hydroxymethyl)oxan-2-yl]oxy-1,4,6,7,8,8a-hexahydroazulen-2-one | -8.2 |
| 132 | MONA | 5.8 | 519.2412 | [M+H]+ | C24H38O12 | 518.2363 | 4-[(E)-3-[6-[[3,4-dihydroxy-4-(hydroxymethyl)oxolan-2-yl]oxymethyl]-3,4,5-trihydroxyoxan-2-yl]oxybut-1-enyl]-4-hydroxy-3,5,5-trimethylcyclohex-2-en-1-one | -8.0 |
| 165 | MSMS_Public_EXP_VS17 | 8.4 | 547.1789 | [M+Na]+ | C25H32O12 | 524.1894 | [4a,7-dihydroxy-7-methyl-1-[3,4,5-trihydroxy-6-(hydroxymethyl)oxan-2-yl]oxy-1,5,6,7a-tetrahydrocyclopenta[c]pyran-5-yl] 3-(4-methoxyphenyl)prop-2-enoate | -9.5 |
| 171 | MSMS_Public_EXP_VS17 | 9.2 | 595.2018 | [M+H]+ | C28H34O14 | 594.1949 | methyl 7-(hydroxymethyl)-1-[3,4,5-trihydroxy-6-[3-(4-hydroxy-3,5-dimethoxyphenyl)prop-2-enoyloxymethyl]oxan-2-yl]oxy-1,4a,5,7a-tetrahydrocyclopenta[c]pyran-4-carboxylate | -9.3 |
| 172 | MSMS_Public_EXP_VS17 | 7.4 | 599.1952 | [M+H]+ | C27H34O15 | 598.1898 | 5,7-dihydroxy-7-methyl-1-[3,4,5-trihydroxy-6-[3-(4-hydroxy-3,5-dimethoxyphenyl)prop-2-enoyloxymethyl]oxan-2-yl]oxy-4a,5,6,7a-tetrahydro-1H-cyclopenta[c]pyran-4-carboxylic acid | -9.0 |
| 176 | MSMS_Public_EXP_VS17 | 8.4 | 757.2550 | [M+H]+ | C34H44O19 | 756.2477 | methyl 7-(hydroxymethyl)-1-[3,4,5-trihydroxy-6-[[3,4,5-trihydroxy-6-[3-(4-hydroxy-3,5-dimethoxyphenyl)prop-2-enoyloxymethyl]oxan-2-yl]oxymethyl]oxan-2-yl]oxy-1,4a,5,7a-tetrahydrocyclopenta[c]pyran-4-carboxylate | -9.2 |
| 180 | MSMS_Public_EXP_VS17 | 6.8 | 579.2070 | [M-H]- | C28H36O13 | 580.2156 | Tortoside A | -8.9 |
| 252 | MSMS_Public_EXP_VS17 | 6.1 | 391.1960 | [M+CH3COO]- | C16H28O7 | 332.1835 | 2-(hydroxymethyl)-6-[(1,3,3-trimethyl-2-oxabicyclo[2.2.2]octan-6-yl)oxy]oxane-3,4,5-triol | -6.9 |

MW* represents Molecular weight
